# Heteropoly Acid-Based Catalysts for Hydrolytic Depolymerization of Cellulosic Biomass

**DOI:** 10.3389/fchem.2020.580146

**Published:** 2020-09-25

**Authors:** Xiaoxiang Luo, Hongguo Wu, Chuanhui Li, Zhengyi Li, Hu Li, Heng Zhang, Yan Li, Yaqiong Su, Song Yang

**Affiliations:** ^1^State Key Laboratory Breeding Base of Green Pesticide and Agricultural Bioengineering, Key Laboratory of Green Pesticide and Agricultural Bioengineering, Ministry of Education, State-Local Joint Laboratory for Comprehensive Utilization of Biomass, Center for Research and Development of Fine Chemicals, Guizhou University, Guiyang, China; ^2^Laboratory of Inorganic Materials and Catalysis, Schuit Institute of Catalysis, Department of Chemical Engineering and Chemistry, Eindhoven University of Technology, Eindhoven, Netherlands

**Keywords:** cellulose hydrolysis, heteropoly acid, biomass conversion, green chemistry, sustainable catalysis

## Abstract

Cellulose is the most abundant source of biomass, which can be converted into monosaccharide or other chemical platform molecules for the sustainable production of chemicals and fuels. Acid catalysts can promote hydrolytic degradation of cellulose into valuable platform molecules, which is of great significance in the development of chemicals and biofuels. However, there are still some shortcomings and limitations of the catalysts for the hydrolytic degradation of cellulosic biomass. Heteropoly acid (HPA), as a green catalyst, seems to be more conducive to the degradation of cellulosic biomass due to its extreme acidity. HPAs can be designed in homogeneous and heterogeneous systems. Moreover, they can be easily separated from the products in both systems by a simple extraction process. According to the unique properties of HPAs (e.g., good solubility, high thermal stability, and strong acidity), using heteropoly acid-based catalysts to depolymerize and convert cellulose into value-added chemicals and biofuels has become one of the most remarkable processes in chemistry for sustainability. In this review, the characteristics, advantages, and applications of HPAs in different categories for cellulose degradation, especially hydrolysis hydrolytic degradation, are summarized. Moreover, the mechanisms of HPAs catalysts in the effective degradation of cellulosic biomass are discussed. This review provides more avenues for the development of renewed and robust HPAs for cellulose degradation in the future.

## Introduction

**Scheme 1:**
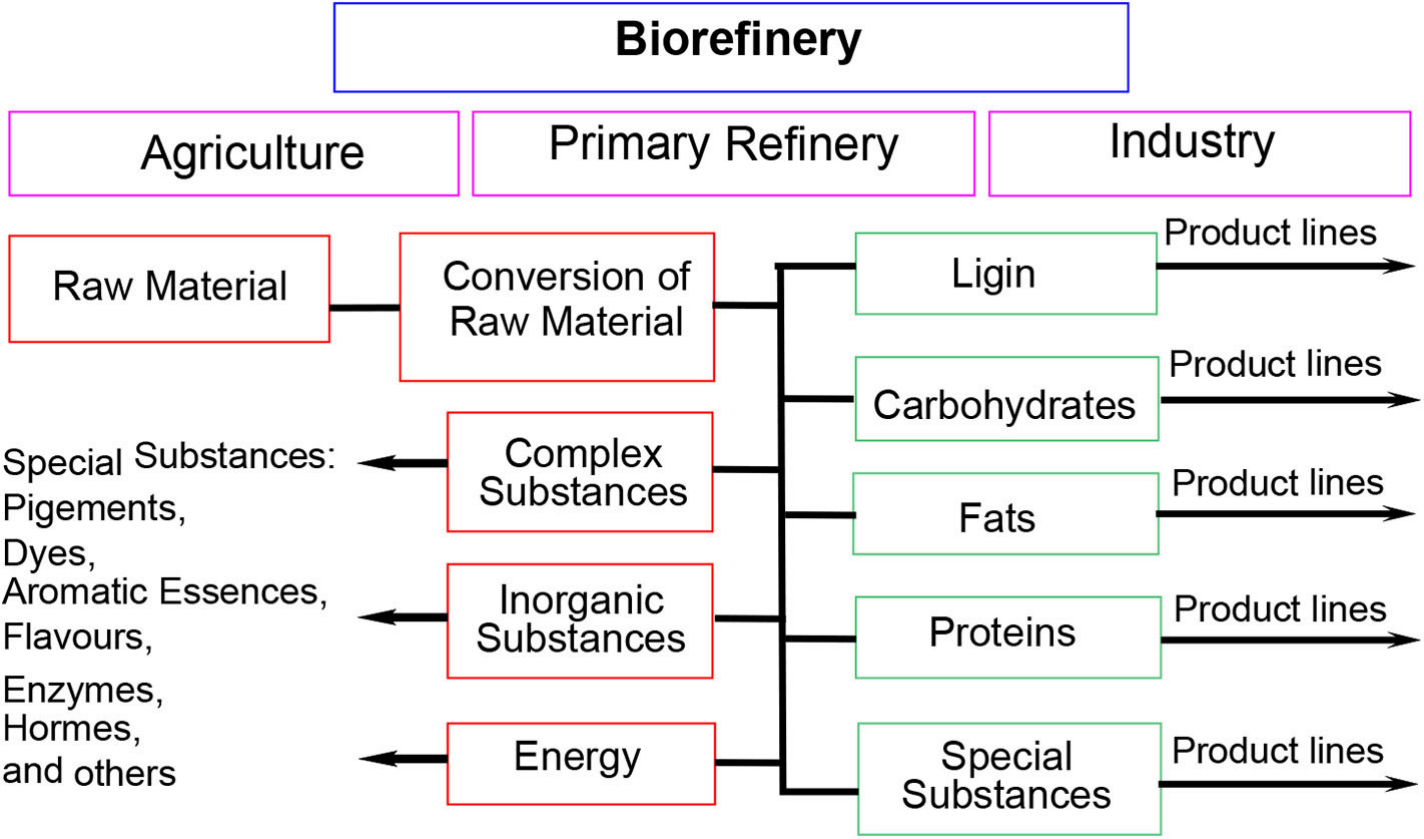
Biorefinery for the development of industrially relevant products.

With the continuous exploitation of human beings, the amount of fossil energy that is the most main energy consumed in the world is decreasing gradually. Therefore, developing a sustainable renewable energy source that can provide valuable chemicals and biofuels is of great importance (Zhang et al., 2015). To furnish the desired bioproducts, some resources are needed which can provide us with basic elements such as C, H, and O in luxuriant amounts (Dhepe and Fukuoka, 2008). Compared with other energy sources (e.g., coal, oil, and natural gas), biomass energy has incomparable advantages as a renewable energy source with little pollution. Moreover, biomass has become the focus of development because of its universality, abundance, and low pollution. In some biorefinery typically using biomass instead of oil, biomass can be converted into energy and relevant bioproducts through multifarious platforms (Chang et al., 2013; Hu et al., 2015; Li et al., 2016; Liu et al., 2016). Especially, biorefinery combines the key technologies of transforming biological raw materials into industrial intermediates and final products (Scheme 1; Kamm and Kamm, 2004; Dhepe and Fukuoka, 2008; Li et al., 2015; Liu and Zhang, 2015; Zhang et al., 2018). Therefore, the utilization of biomass is a wonderful choice to develop sustainable and renewable energy.

**Scheme 2:**
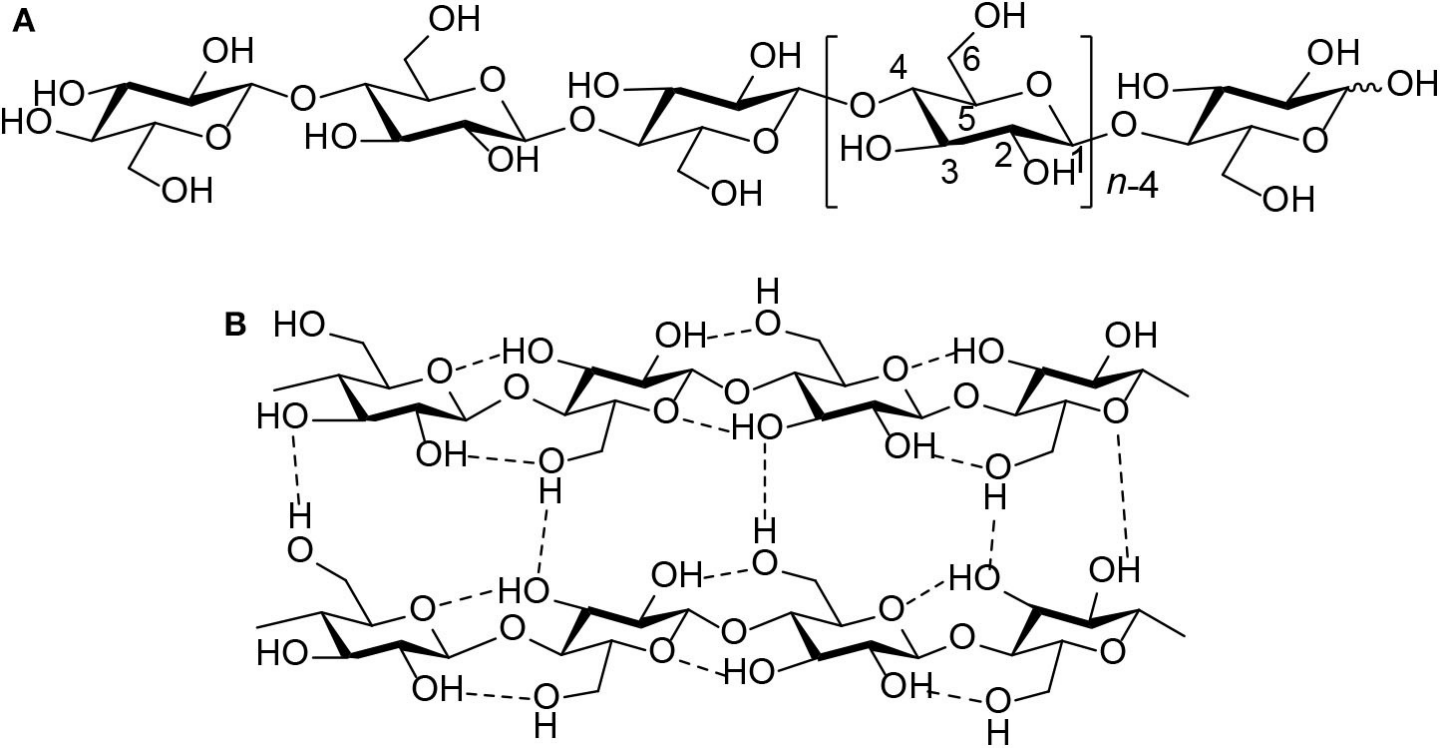
**(A)** Molecular structure of cellulose. **(B)** Intermolecular and intramolecular hydrogen bond interactions in cellulose (*n* = DP, degree of polymerization).

As the most abundant source of biomass and one of the most widely distributed and abundant polysaccharides in nature, cellulose provides more possibilities for sustainable chemicals and fuels in the future (Huber et al., 2006; Climent et al., 2014; Sheldon, 2014; To et al., 2015). Hence, the conversion of cellulose could become a significant part of biomass utilization, which plays an important role in the development of chemicals and fuels in the future. However, cellulose is a homopolymer of D-glucose made up of β-1,4 glycosidic bonds (Scheme 2A). Its intramolecular and intermolecular hydrogen bonds are formed (Scheme 2B), resulting in its water insolubility (Geboers et al., 2011; Wang et al., 2012; Harada et al., 2014; Yabushita et al., 2014). Therefore, the processes of cellulose conversion are complicated because this biopolymer is insoluble in water, and most organic solvents (Klemm et al., 2005; Albert et al., 2014).

**Scheme 3:**
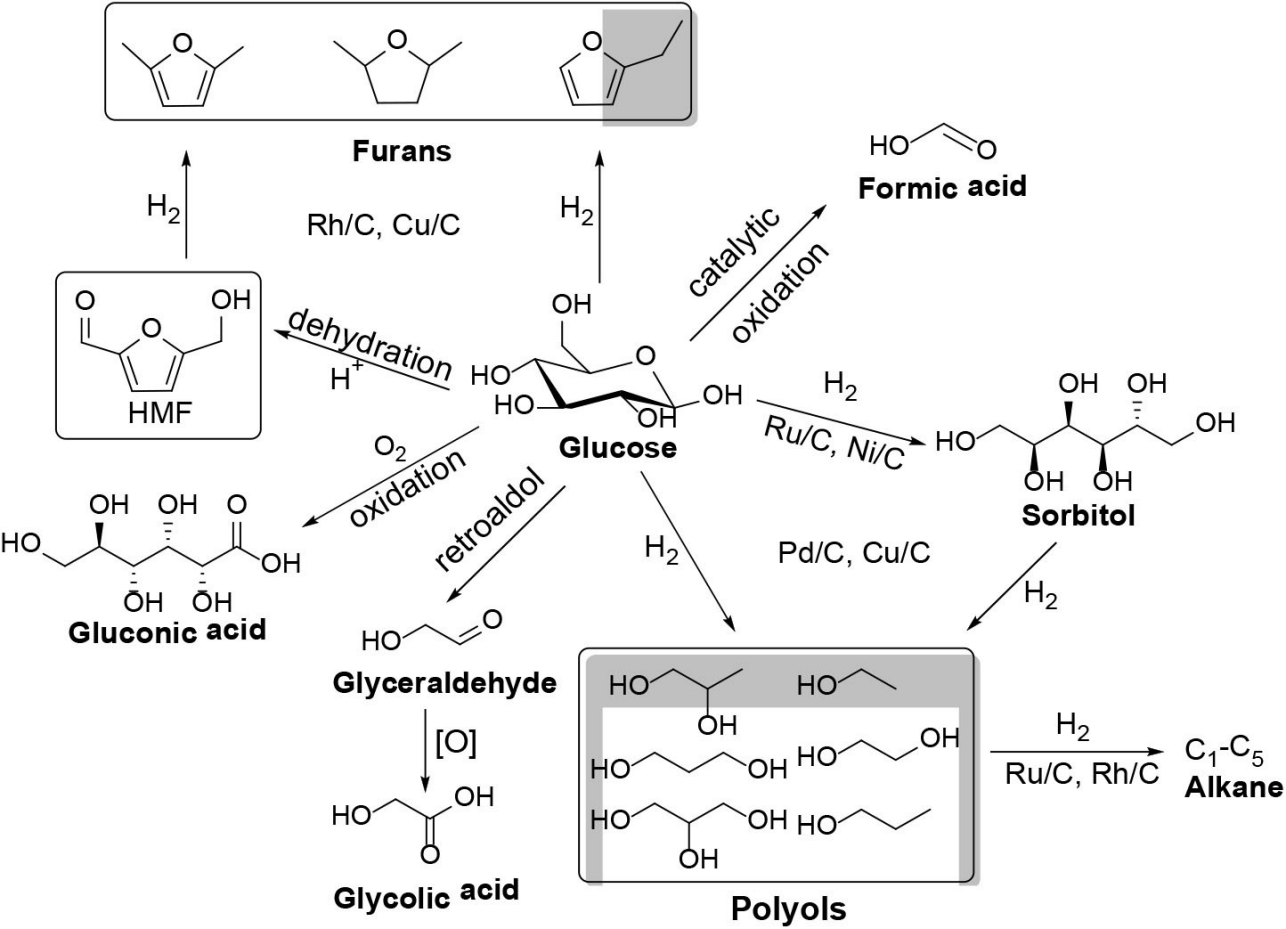
The main reaction network of glucose hydrogenation and oxidation.

Many homogeneous and heterogeneous acid catalysts were found to be capable of converting cellulose into glucose, a monosaccharide which is an important component for synthesizing different kinds of chemicals and fuels (Scheme 3), such as furans (Li et al., 2014), ethanol (Chheda et al., 2007), polyhydric alcohol (Geboers et al., 2010; de Op Beeck et al., 2013; Xie et al., 2014; He et al., 2018), furfural (Yu et al., 2009b; Feng et al., 2011; Li et al., 2013; Pan et al., 2013), organic acid (Yan et al., 2014; Zhang et al., 2014; Ren et al., 2015), and so on. Several excellent review literature on the conversion of cellulose have been reported, with focus on the discussion of different solid acid catalysts (e.g., Amberlyst-15, PCPs–SO_3_H, BC–SO_3_H, CMK-3-SO_3_H, Zn–Ca–Fe, CsH_2_PW_12_O_40_, Ru/CMK-3, Fe_3_O_4_-SBA–SO_3_H, CaFe_2_O_4_, H_3_PW_12_O_40_, H_5_BW_12_O_40_, H_5_AlW_12_O_40_, H_5_GaW_12_O_40_, and H_6_CoW_12_O_40_) for hydrolysis of cellulose (Table 1; Kobayashi et al., 2010; Pang et al., 2010; Rinaldi et al., 2010; Tian et al., 2010, 2011; Wu et al., 2010; Akiyama et al., 2011; Komanoya et al., 2011; Lai et al., 2011; Ogasawara et al., 2011; Zhang et al., 2011).

**Table 1:** Cellulose hydrolysis over different solid acid catalysts.

**Catalyst**	**Solvents**	**Temp (^**°**^C)**	**Time (h)**	**Glucose/TRS yield TRS yield/%**	**References**
Amberlyst-15	[BMIm]Cl/H_2_O	100	5	11.0	Rinaldi et al., 2010
PCPs–SO_3_H	H_2_O	120	3	5.30	Akiyama et al., 2011
BC–SO_3_H	H_2_O	90	1	19.8	Wu et al., 2010
CMK-3-SO3H	H_2_O	150	24	74.5	Pang et al., 2010
Zn–Ca–Fe	H_2_O	160	20	29	Zhang et al., 2011
CsH_2_PW_12_O_40_	H_2_O	160	6	27.0	Tian et al., 2011
Ru/CMK-3	H_2_O	230	24	34.2	Kobayashi et al., 2010
Fe_3_O_4_-SBA–SO_3_H	H_2_O	150	3	26.0	Lai et al., 2011
CaFe_2_O_4_	H_2_O	150	24	36.0	Komanoya et al., 2011
H_3_PW_12_O_40_	H_2_O	180	2	50.5	Tian et al., 2010
H_5_BW_12_O_40_	H_2_O	60	6	77.0	Ogasawara et al., 2011
H_5_AlW_12_O_40_	H_2_O	60	24	68.0	Ogasawara et al., 2011
H_5_GaW_12_O_40_	H_2_O	60	24	62.0	Ogasawara et al., 2011
H_6_CoW_12_O_40_	H_2_O	60	24	59.0	Ogasawara et al., 2011

**Figure 1:**
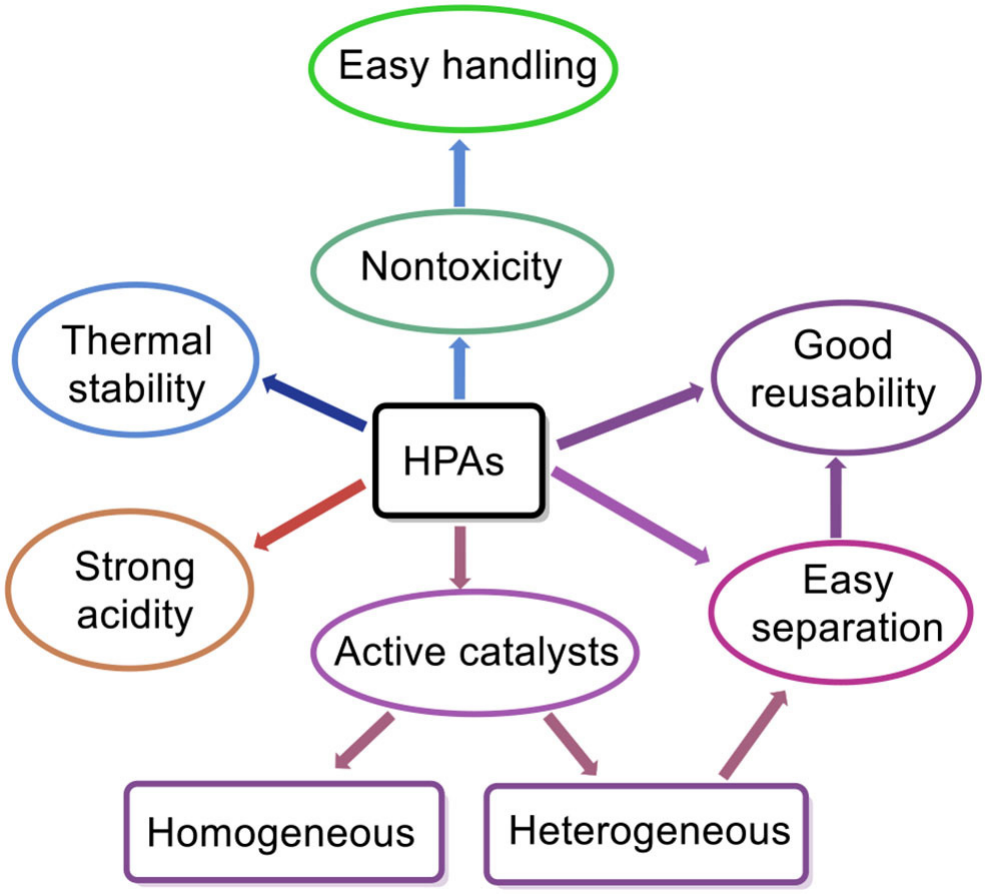
Some unique properties of heteropoly acids (HPAs).

**Figure 2:**
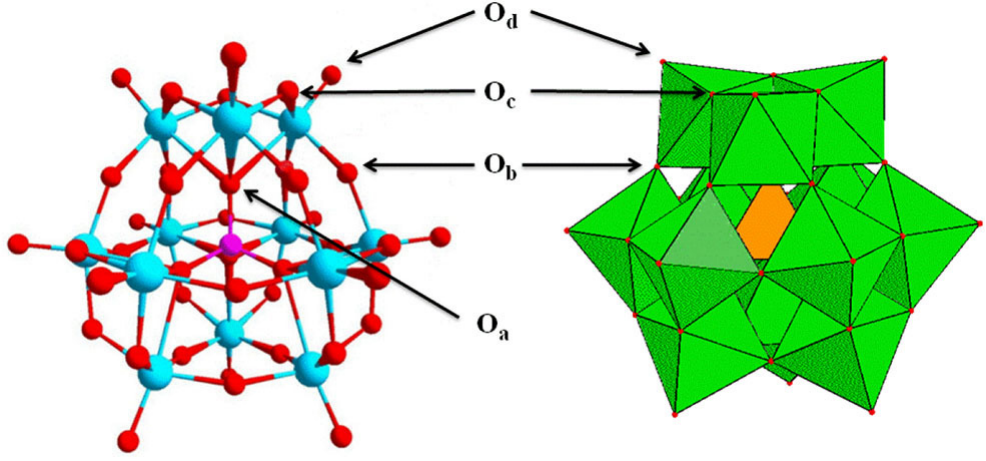
The structure of common Keggin-type HPA.

Compared with other solid acids, HPA catalysts are more favorable for hydrolysis of cellulose, considering that they have some unique properties such as stronger acidity compared to other mineral acids (e.g., H_2_SO_4_, HCl), high redox properties, good thermal stability, easy separation, good reusability, fewer side products, less waste generation, non-toxicity, and easy handling, as illustrated in Figure 1 (Kozhevnikov, 1987, 1998; Mizuno and Misono, 1994; Kaur and Kozhevnikov, 2002; Yu et al., 2009a; Yang et al., 2011; Deng et al., 2012; Reddy et al., 2012; de Op Beeck et al., 2013). Furthermore, HPA catalysts can be used in both homogeneous and heterogeneous systems. The most significant advantage of the heterogeneous system is that the HPA catalysts can be easily separated from the reaction products, thus improving the catalyst reusability. The most common HPAs are Keggin-type HPAs, which have the anions of [XM_12_O_40_]^n−^, where X is a heteroatom (e.g., P, Si) and M is a metal ion (e.g., W^6+^, Mo^6+^). Among them, H_3_PW_12_O_40_ is the most common Keggin-type HPA. Moreover, H_3_PW_12_O_40_ with strong acidity is highly soluble in water and can completely dissociate protons, which facilitates contact with the heterogeneous substrates, thus endowing enhanced reaction rates (Tian et al., 2010). Besides, the basic structural units of Keggin-type HPAs are oxygen-containing tetrahedron and octahedron anions in good symmetry and low charge density (Figure 2).

Given the unique features and rapid development of HPAs-based catalysts in the preliminary degradation of cellulosic biomass with high efficiency, this review mainly reports the design and structural characterization of different types of HPAs employed for hydrolytic depolymerization of cellulosic biomass. In addition, the properties and preparation methods of HPAs as well as their advantages in the hydrolysis of cellulose are discussed. Also, possible research trends of HPAs in the future are forecasted.

## HPAs-Based Catalysts

### HPA Properties and Classification

HPA catalysts have attracted people's attention, and are known as effective, environmentally friendly and economically viable acid catalysts (Kozhevnikov, 1998), which can be used in either homogeneous or heterogeneous state (Izumi et al., 1983; Nowinska et al., 1996; Song and Lee, 2004; Bennardi et al., 2010; Zhao et al., 2013). Compared with a homogeneous system, the heterogeneous system has the advantage that the catalyst is easy to separate from the reaction products. Due to these special features, HPA catalysts are more beneficial to the degradation of cellulosic biomass than other catalysts.

HPA catalysts can be classified into the following categories: (1) Keggin-type HPAs, the most common HAPs with the anions of [XM_12_O_40_]^n−^, where X is a heteroatom (e.g., P, Si) and M is a metal ion (e.g., W^6+^, Mo^6+^). (2) Substituted HPAs, the H^+^ ions on conventional HPAs are substituted by monovalent cations, such as Cs^+^ ions. (3) Supported HPAs, the heteropoly compounds are supported on suitable supporters, such as silica. (4) Assembled HPAs, the new catalysts with higher activity are synthesized by self-assembly of HPAs with other substances, such as ionic liquids. (5) Other types, new types of heterogeneous catalysts formed by an effective combination of HPAs with other substances.

### Catalyst Preparation Methods

**Scheme 4:**
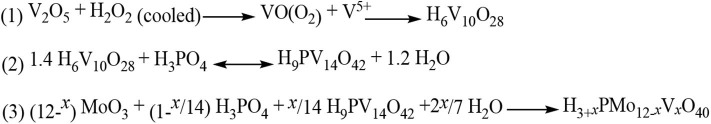
The overall scheme for the synthesis of Mo-V-P HPA catalysts.

**Scheme 5:**
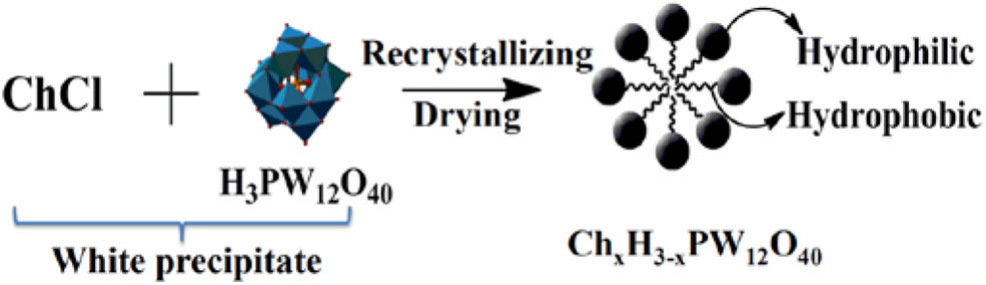
The synthetic procedure of Ch_*x*_H_3−x_PW_12_O_40_ catalyst.

**Scheme 6:**
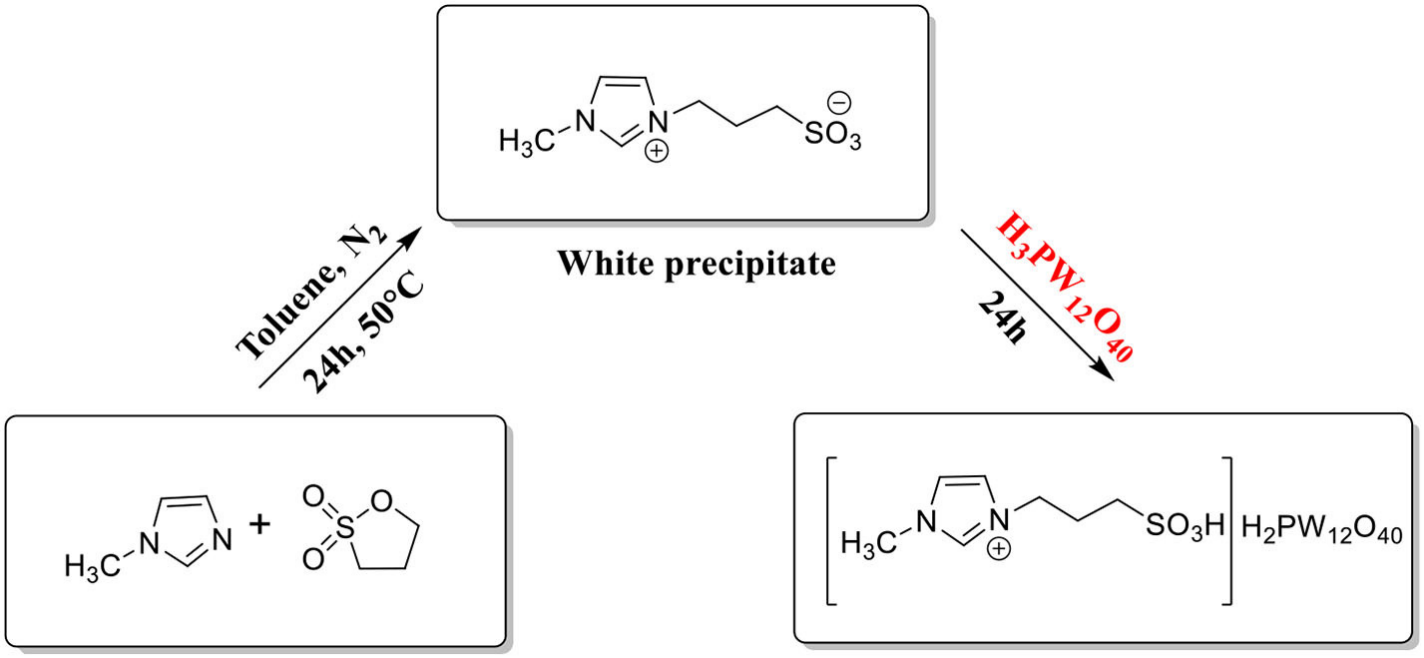
The synthetic routes to [C_4_H_6_N_2_(CH_2_)_3_SO_3_H]_3_PW_12_O_40_.

Up to the present, many patents and literature on the preparation of HPAs were published, including e.g., traditional acidification method incorporated with an ether extraction, ion exchange method, impregnation method, and sol-gel method (Bechtold and Square, 1950; Laferty and John, 1966; Chiola and Lawrence, 1969; Chiola et al., 1969; Izumi et al., 1995; Vázquez et al., 2000; Mrowiec-Białoń et al., 2002; Cardoso et al., 2004; Yang et al., 2005; Ahmed et al., 2011). The traditional synthesis method of conventional HPAs is an acidification process coupled with an ether extraction, which is commonly used for the synthesis of phosphotungstate HPA catalysts. In this method, the HPA is prepared by mixing heteroatom oxygen-containing acid and ligand oxide in a certain proportion, heating, refluxing, and then acidifying to obtain HPA. The HPA is dissolved in ether, and the solid HPA is obtained after ether volatilization (Chiola and Lawrence, 1969; Chiola et al., 1969). However, the acidification procedure coupled with an ether extraction has the disadvantages of low safety due to the use of toxic substances and low yield of some HPAs. In connection with this, the ion-exchange method has been developed, which first obtains the corresponding ammonium salt, and then obtains HPA through hydrogen ion-exchange resin. This method avoids the use of ether and improves process safety, but the production cycle is relatively long (Bechtold and Square, 1950; Laferty and John, 1966). For some supported HPAs, the impregnation method is commonly used. The method is to add the carrier into the solution of HPA with a certain concentration, stir for a long time at a certain temperature, stand still, and then remove the excess solvent to obtain the catalyst. The method is simple and safe, but there are some problems such as the loss of catalyst activity and limited recycling ability. Therefore, the support should be screened and optimized (Vázquez et al., 2000; Cardoso et al., 2004; Liu et al., 2004). The sol-gel method is to add raw materials to the HPA colloid and obtain the HPA catalyst through a series of specific processes, such as drying and heat treatment. This method can be used to prepare catalysts at a low temperature or mild condition, which is widely used in manufacturing nanomaterials catalysts. Likewise, this method has the disadvantages of low security and long cycle (Izumi et al., 1995; Mrowiec-Białoń et al., 2002; Yang et al., 2005). Some examples of catalysts for the preparation of HPAs are as follows:

1. Keggin-type Mo-V-P heteropoly acid catalysts (Scheme 4; Zhizhina and Odyakov, 2008; Odyakov and Zhizhina, 2009): Firstly, V_2_O_5_ was dissolved in cold diluted H_2_O_2_ solution to form peroxy complexes, which was gradually decomposed with oxygen to give H_6_O_10_V_28_. Then, by adding excess H_3_PO_4_, H_6_O_10_V_28_ acid produced was immediately stabilized to give H_9_PV_14_O_42_, following by addition into the boiling suspension of MoO_3_ and H_3_PO_4_ to form the catalyst.
2. Substituted heteropoly tungstic acid catalyst Cs_*x*_H_3−x_PW_12_O_40_ (*x* = 1–3): It is usually prepared by titration method (Okuhara et al., 1992, 1996, 2000; Tian et al., 2011). At room temperature, dropping 0.10 mol dm^−3^ Cs_2_CO_3_ aqueous solution into 0.8 mol dm^−3^ of H_3_PW_12_O_40_ aqueous solution at a constant rate was stirred continuously. Then the resulting solution was aged overnight at room temperature and heated at 50°C to obtain the catalyst.
3. The synthesis of catalyst (HOCH_2_CH_2_N(CH_3_)_3_)_x_ H_3−x_PW_12_O_40_ (abbreviated as Ch_x_H_3−x_PW_12_O_40_) is shown in Scheme 5 (Duan et al., 2013; Zhang et al., 2016): choline chloride (ChCl, HOCH_2_CH_2_N(CH_3_)_3_Cl) (3.34 mmol) was added to H_3_PW_12_O_40_ (3.34 mmol) solution and the white precipitate was formed after stirring in 20 mL distilled water for 8 h at room temperature. Then the white precipitate was washed with distilled water, followed by recrystallization twice with CH_3_CN, drying at 60°C, and finally getting the white product ChH_2_PW_12_O_40_ (HOCH_2_CH_2_N(CH_3_)_3_H_2_PW_12_O_40_).
4. The self-assembled HPA ionic liquid catalyst [C_4_H_6_N_2_(CH_2_)_3_SO_3_H]_3_PW_12_O_40_ (Scheme 6; Leng et al., 2010): 0.11 mol methyl imidazole and 0.10 mol 1,3-propane sulfone were dissolved in 20 mL toluene under nitrogen and stirred for 24 h at 50°C. The resulting white precipitate is filtered and washed three times with ether and then dried in a vacuum. Then it was added to the aqueous solution of H_3_PW_12_O_40_ (0.02 mol) and stirred for 24 h at room temperature. Then water was removed in vacuum to obtain solid products, washed with water and ethanol, and dried at 100°C for 3 h.

## Hydrolytic Depolymerization of Biomass With HPAS-Based Catalysts

### Keggin-Type HPAs

Keggin-type HPAs are the most common HPA catalysts, which have great application prospects for biomass degradation. The potential of HPAs for catalyzing the hydrolysis of cellulose to glucose has been explored.

#### Cellulose Conversion to Glucose

**Scheme 7:**
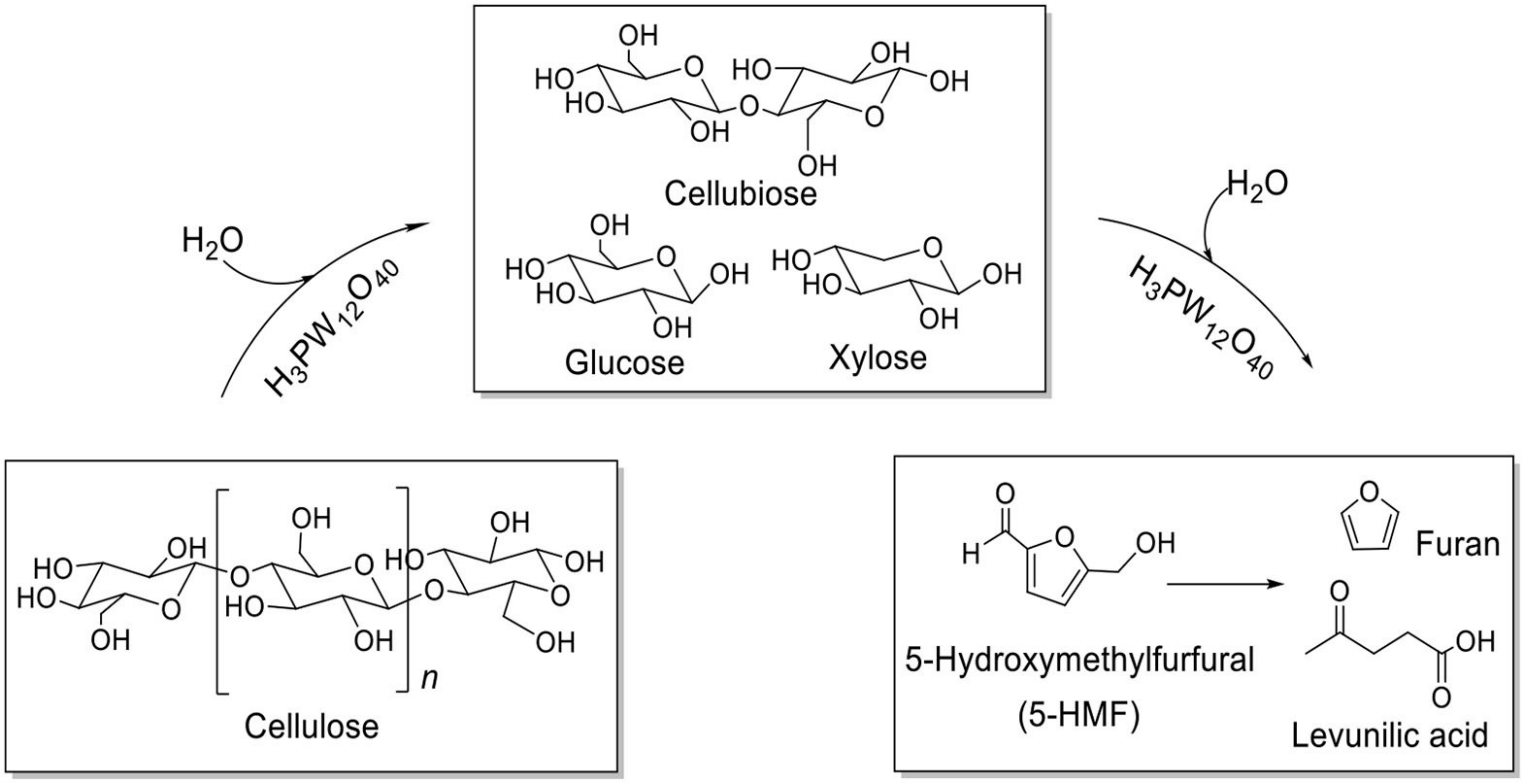
Main products and by-products of cellulose conversion by an acid catalyst H_3_PW_12_O_40_.

**Figure 3:**
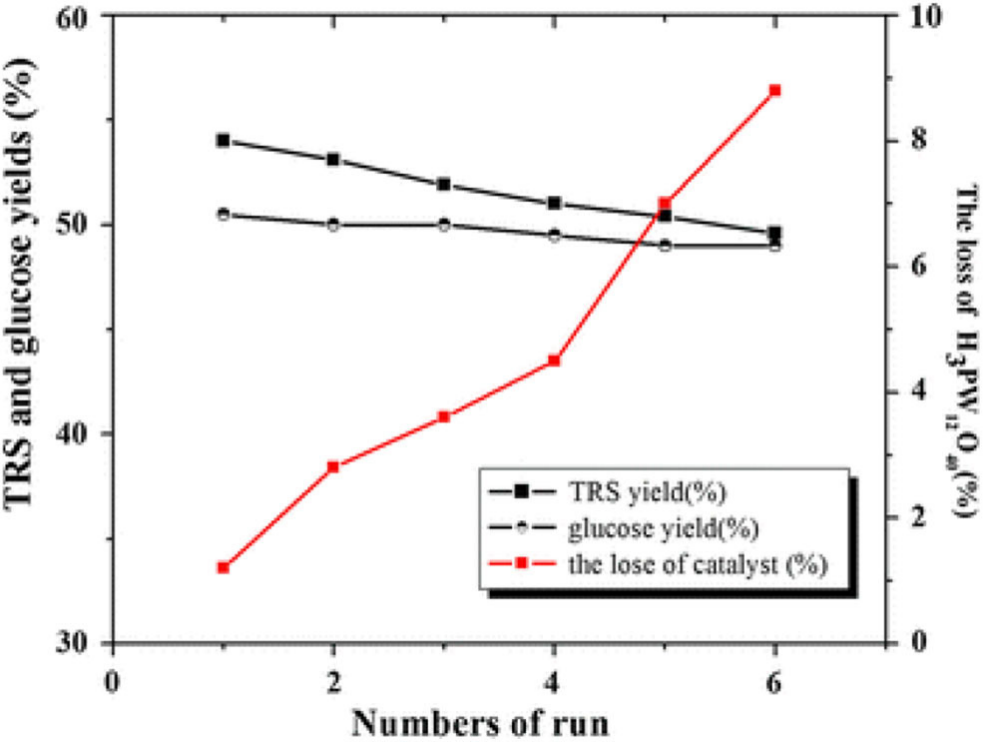
The recyclability of H_3_PW_12_O_40_ in six consecutive reaction cycles. Reaction conditions: 0.1 g cellulose, 0.08 mmol catalyst, 5 mL water at 180°C for 2 h. Reproduced with permission from Tian et al. (2010).

Tian et al. developed the heteropoly acid H_3_PW_12_O_40_ for selective conversion of cellulose (Scheme 7). The heteropoly acid catalyst can promote cellulose hydrolysis, achieving the glucose selectivity and yield of 92.3 and 50.5%, respectively at 180°C for 2 h with 0.10 g cellulose amount. The authors also studied and optimized reaction parameters influencing the hydrolysis of cellulose and determined the most optimizing reaction conditions. Compared with HCl, H_3_PW_12_O_40_ had greater catalytic activity and glucose selectivity under identical reaction conditions with the same acid concentration (Table 2: 0.2 mmol HCl is equivalent to 0.06 mmol H_3_PW_12_O_40_, and 0.06 mmol H_3_PW_12_O_40_ had higher catalytic activity). After testing the catalytic activity of H_3_PW_12_O_40_ in six consecutive reaction cycles, the total loss of H_3_PW_12_O_40_ was 8.8% of its initial loss (Figure 3; Tian et al., 2010). It was found that the acid catalyst H_3_PW_12_O_40_ was generally stable and could be recycled by extraction using diethyl ether. However, there was still a little loss of catalyst activity, which led to a slight decrease in the yield of TRS and glucose.

**Table 2:** Hydrolysis of cellulose with different acid catalysts (Tian et al., 2010).

**Catalyst (mmol)**	**Conversion (%)**	**TRS yield (%)**	**Glucose yield (%)**	**Selectivity (%)**
None	0	0	0	0
HCl (0.20 mmol)	25.6	25.3	13.8	53.9
H_3_PW_12_O_40_ (0.01 mmol)	10.4	10.4	8.2	78.8
H_3_PW_12_O_40_ (0.02 mmol)	17.1	16.7	13.8	80.7
H_3_PW_12_O_40_ (0.03 mmol)	23.0	22.1	20.9	90.7
H_3_PW_12_O_40_ (0.05 mmol)	29.7	28.4	26.5	89.2
H_3_PW_12_O_40_ (0.05 mmol)	33.2	32.8	30.7	92.5
H_3_PW_12_O_40_ (0.06 mmol)	38.5	38.5	36.6	95.1
H_3_PW_12_O_40_ (0.07 mmol)	44.0	43.8	41.8	95.0
H_3_PW_12_O_40_ (0.08 mmol)	54.7	54	50.5	92.3
H_3_PW_12_O_40_ (0.09 mmol)	57.4	56.3	51.9	90.4

*TRS, total reducing sugars. Reaction conditions: 0.1 g cellulose, 5 mL water, at 180°C for 2 h*.

**Figure 4:**
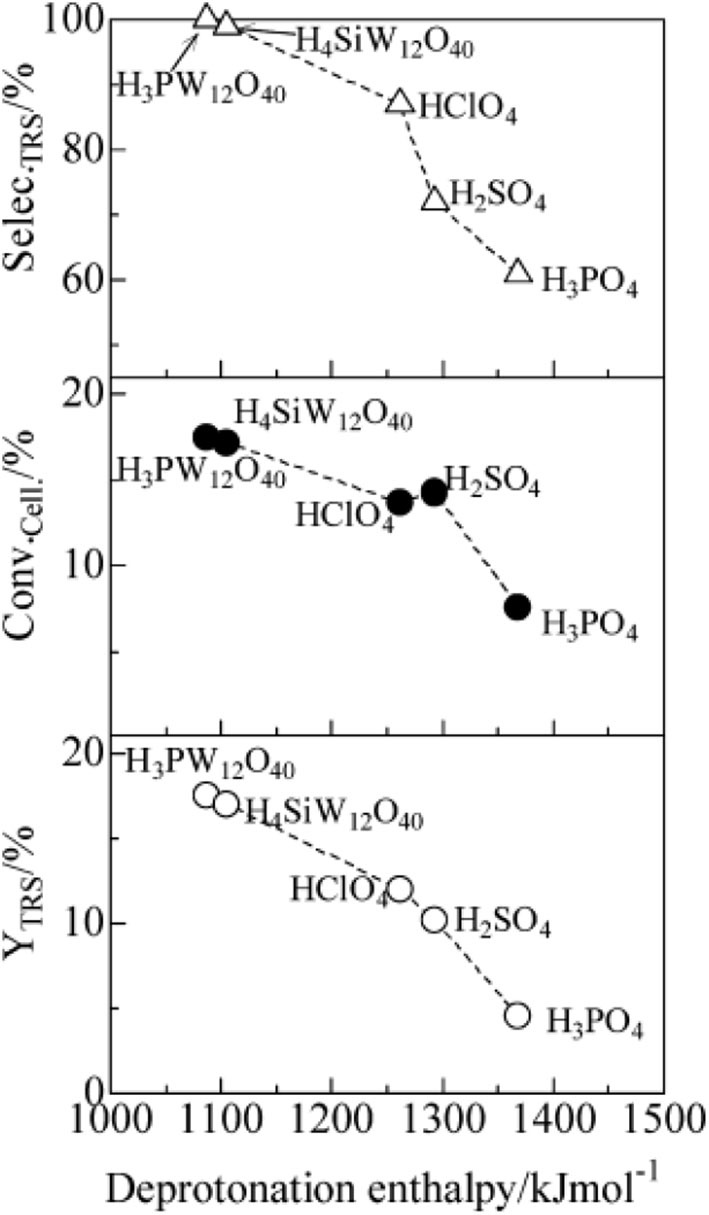
The effect of deprotonation enthalpy (DPE) of acid catalysts on catalysts activity (∘, TRS yield; •, cellulose conversion; Δ, TRS selectivity of cellulose hydrolysis). Reaction conditions: 0.58 mmol cellulose, 6 mL H_2_O, 6 mol catalyst (M_n/3_PW_12_O_40_) at 150°C for 2 h. Reproduced with permission from Macht et al. (2008) and Shimizu and Satsuma (2011).

**Figure 5:**
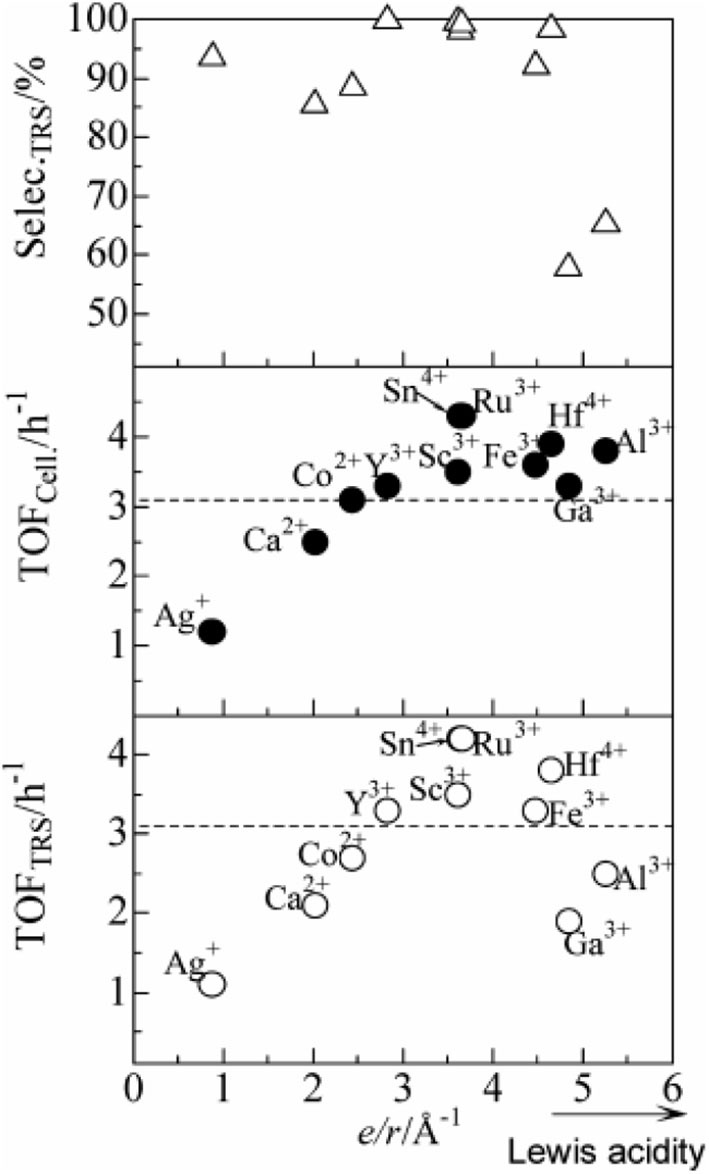
The effect of Lewis acid on the catalytic rate of catalysts (∘, TRS yield; •, cellulose conversion; Δ, TRS selectivity of cellulose hydrolysis). Reaction conditions: 0.58 mmol cellulose, 6 mL H_2_O, 6 mol catalyst (M_n/3_PW_12_O_40_) at 150°C for 2 h. Reproduced with permission from Shimizu and Satsuma (2011).

In 2009, Shimizu et al. studied the effects of Brønsted and Lewis acidities on the activity and selectivity of HPA catalysts for hydrolysis of cellulose (Furukawa et al., 2009). The authors also reported that HPAs (i.e., H_3_PW_12_O_40_ and H_4_SiW_12_O_40_) could catalyze the hydrolysis of cellulose with high selectivity to glucose. It was concluded that for Brønsted acid catalysts, the activity increases with the decrease in the deprotonation enthalpies (DPE, for the description of the acid strength of HPAs) (Macht et al., 2008; Shimizu and Satsuma, 2011), indicating that stronger Brønsted acidity is more beneficial to the hydrolysis of cellulose (The effect of DPE on catalysts activity shown in Figure 4). In other words, stronger Brønsted acid is more beneficial to the hydrolysis of β-1,4 glucosidic bonds of cellulose. More interestingly, the obtained catalyst rates are higher for moderate Lewis acidity, while the catalysts with moderate Lewis acidity have higher TOFs for glucose production than H_3_PW_12_O_40_ (Figure 5). Therefore, the Brønsted and Lewis acidities of HPA catalysts can influence the hydrolysis of cellulose. Moreover, with the increase of the catalyst dosage, the yield of glucose increased, which is due to the increase in the number of catalytic active sites. As shown in Figure 4, the lower DPE corresponds to stronger acidity of the catalysts, which is responsible for higher catalytic activity, with the TRS yield in the order of H_4_SiW_12_O_40_ > H_3_PW_12_O_40_ > HClO_4_ > H_2_SO_4_ > H_3_PO_4_.

**Scheme 8:**
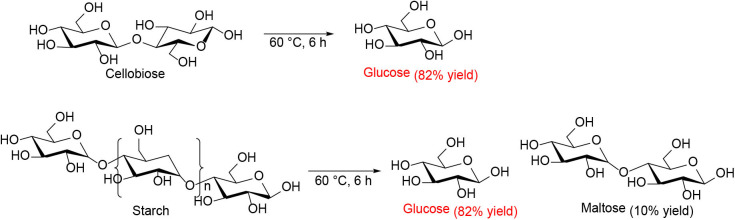
Transformation of cellobiose and starch in concentrated H_5_BW_12_O_40_ aqueous solutions (0.70 mol/L, 2 mL).

**Figure 6:**
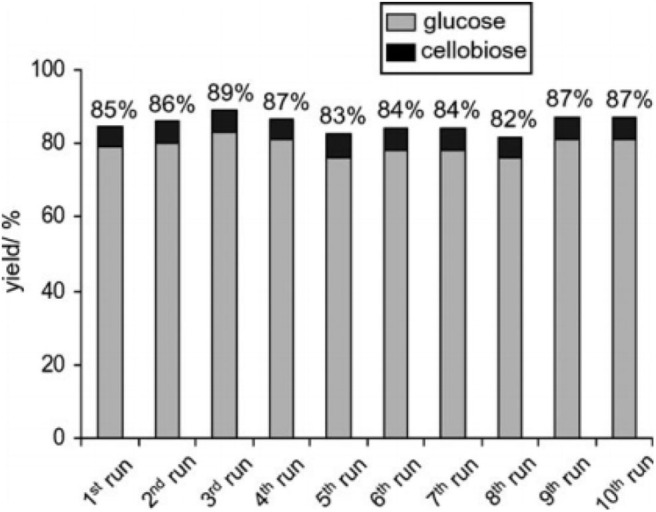
Recyclability of H_5_BW_12_O_40_ for the conversion of pretreated cellulose. Reaction conditions: 100 mg pretreated cellulose (the cellulose was mercerized and then ball-milled), 0.70 mol/L, 2 mL H_5_BW_12_O_40_ solution, 60°C for 24 h. Reproduced with permission from Ogasawara et al. (2011).

Ogasawara et al. found that the highly negatively charged HPAs (e.g., H_5_BW_12_O_40_, H_5_AlW_12_O_40_, and H_5_GaW_12_O_40_) could efficiently promote the hydrolysis of crystalline cellulose into glucose in concentrated aqueous solutions (Ogasawara et al., 2011). In particular, H_5_BW_12_O_40_ showed a superior yield of glucose (77%) for 48 h in 0.7 mol/L cellulose solution at a lower reaction temperature (60°C), which avoided the formation of undesirable byproducts such as humic substances and dehydration products. And its performance is much better than the commonly utilized mineral acids and HPAs, such as H_2_SO_4_, HCl, H_3_PW_12_O_40_, and H_4_SiW_12_O_40_ (Table 3). Procedures for saccharification contains two important points: turning the acidity of catalysts and decreasing the crystallinity of cellulose by breaking intermolecular hydrogen bonding that can be achieved by concentrated H_5_BW_12_O_40_ aqueous solutions (0.70 mol/L). Besides crystalline cellulose, the present system was suitable for the selective transformation of cellobiose and starch into glucose with a good yield of 82 and 85%, respectively (Scheme 8). Also, H_5_BW_12_O_40_ and saccharide can be completely separated from aqueous reaction solutions by extraction using alcoholic solvents due to their different solubility. And the retrieved highly negatively charged HPA H_5_BW_12_O_40_ can be used repeatedly at least ten times without significant performance loss (Figure 6; Ogasawara et al., 2011).

**Table 3:** Hydrolysis of cellulose using different concentrations of aqueous acidic solutions (Ogasawara et al., 2011).

**No**.	**Acid catalysts**	**Concentration**		**Glucose yield (%)**
		**Anion (mol/L)**	**Proton (mol/L)**	
1	H_3_PW_12_O_40_	0.70	2.1	8
2^a^	H_3_PW_12_O_40_	0.60^b^	3.5	18
3	H_4_SiW_12_O_40_	0.70	2.8	37
4^a^	H_4_SiW_12_O_40_	0.70	3.5	61
5	H_5_BW_12_O_40_	0.70	3.5	77
6	H_5_BW_12_O_40_	0.40	2.0	4
7	H_5_AlW_12_O_40_	0.70	3.5	68
8	H_5_GaW_12_O_40_	0.70	3.5	62
9	H_6_CoW_12_O_40_	0.70	4.2	59
10	H_2_SO_4_	1.75	3.5	<1
11	H_2_SO_4_	4.5^c^	9.0	5
12	HCl	3.5	3.5	4
13	HCl	6.0^c^	6.0	9

a The proton concentration was adjusted using H_2_SO_4_.

b Saturated concentration.

c The same as that of the H_5_BW_12_O_40_ solution (0.7 mol/L).

*Reaction conditions: crystalline cellulose (100 mg), aqueous acidic solution (2 mL), 60°C for 48 h*.

**Scheme 9:**
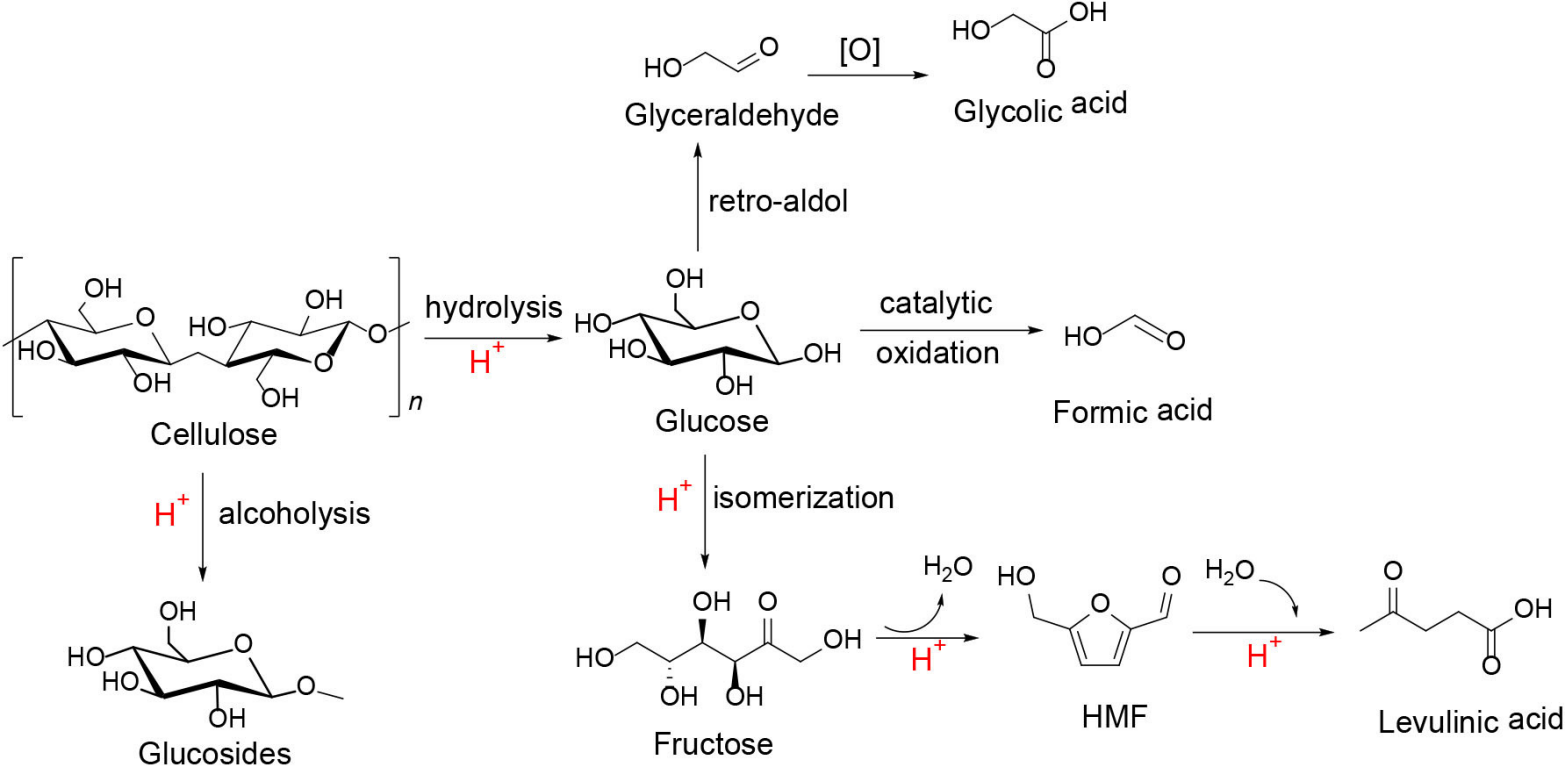
Conversion of cellulose to other substance with an HPA catalyst (H^+^, HPA catalyst).

For the conversion pathways, the HPAs can initially hydrolyze cellulose into glucose that can be further converted into other substance in the assistance of their bifunctional properties (i.e., acidity and redox property), such as catalytic oxidation of cellulose to formic acid (Wölfel et al., 2011; Albert et al., 2012, 2014; Gromov et al., 2016; Lu et al., 2016), direct conversion of cellulose to glycolic acid (Zhang et al., 2012), and cellulose transformation into methyl glucosides (Scheme 9; Zheng et al., 2018).

#### Cellulose Conversion to Formic Acid

**Scheme 10:**
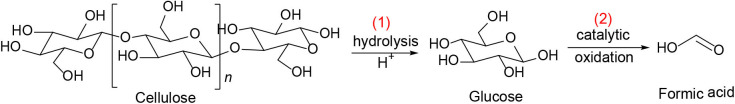
The stages of converting cellulose into formic acid.

Conversion of cellulose to formic acid using HPAs as catalysts is an efficient and environmentally friendly catalytic system (Lu et al., 2016). This conversion system involves two stages (Scheme 10): (1) acid-catalyzed the hydrolysis of cellulose into glucose, and (2) oxidation of glucose to formic acid with an oxidizer (C-C bond in glucose is oxidized and cracked by HPA catalysts) (Albert et al., 2014; Gromov et al., 2016).

**Scheme 11:**
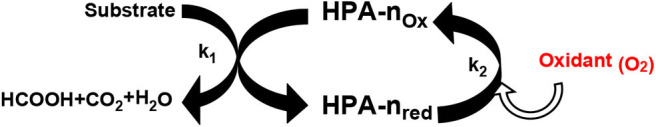
The redox cycle of the catalyst H_5_PV_2_Mo_10_O_40._

In 2014, different Keggin-type HPAs H_3+n_[PVnMo_12−n_O_40_] (*n* = 0–6) were synthesized, and the optimized HPA catalyst system H_8_[PV_5_Mo_7_O_40_] could selectively oxidize biomass to formic acid (Albert et al., 2014). The whole reaction mechanism was divided into three steps: acid-assisted hydrolysis of the insoluble substrate, oxidative cleavage of C–C bonds in the substrate, and re-oxidation of the *in situ* reduced HPA catalyst by molecular oxygen. The acid catalyst redox cycle is shown in Scheme 11. Albert et al. also confirmed that the HPA catalysts screened with glucose as substrate had general reactivity, and the higher V-substituted catalysts (*n* = 2–6) showed better activities than the lower V-substituted catalysts (*n* = 0–1) (Albert et al., 2014). The comparison of the yields of glucose to formic acid under different HPA catalysts is shown in Table 4. Moreover, when H_5_PV_2_Mo_10_O_40_ and *p*-toluenesulfonic acid (TSA) are used as a catalyst and an additive, respectively, the conversion of cellulose was explained as a two-stage process that acid-catalyzed both cellulose hydrolysis and glucose oxidation to formic acid (Wölfel et al., 2011; Albert et al., 2014). In the system, TAS was used for the hydrolysis of cellulose, while H_5_PV_2_Mo_10_O_40_ was used for the oxidant of glucose. It is worth noting that H_8_[PV_5_Mo_7_O_40_] catalyst has pronounced activity for the conversion of cellulose to formic acid, but with the disadvantage of low selectivity toward formic acid.

**Table 4:** The comparison of the yields of glucose to formic acid with different HPA catalysts (Albert et al., 2014).

**Catalyst**	**Combined yield FA + CO_**2**_ (%)**	**pH before reaction**	**pH after reaction**	**Selectivity** **FA + CO_**2**_ (%)**
HPA-0	10	3.32	1.54	40 : 60
HPA-1	12	3.34	1.52	50 : 50
HPA-2	91	3.51	1.30	52 : 48
HPA-3	100	3.39	1.27	56 : 44
HPA-4	97	3.45	1.41	54 : 46
HPA-5	94	3.45	1.41	61 : 39
HPA-6	97	3.24	1.41	58 : 42
H_9_PV_14_O_42_	88	3.68	1.41	58 : 42

*Reaction conditions: 5.0 g (27.8 mmol) glucose and 0.8 mmol catalyst, 100.0 mL H_2_O, 90°C, 8 h, 1,000 rpm. HPA-n, H_3+n_[PVnMo_12−n_O_40_]*.

**Figure 7:**
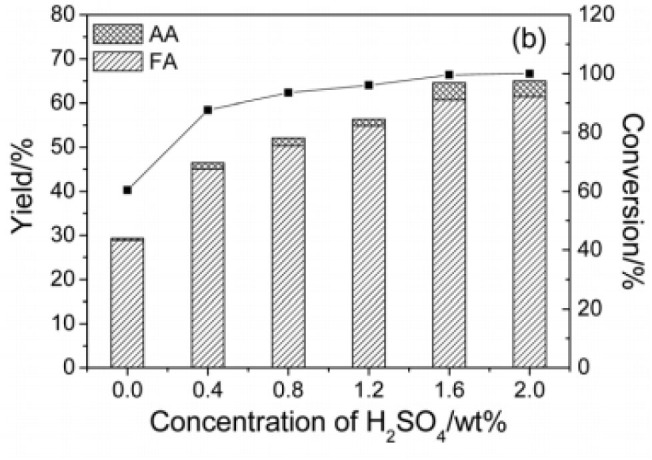
Conversion of cellulose to formic acid (FA) and acetic acid (AA) under different concentration of H_2_SO_4_. Reaction conditions: 0.16 g cellulose, 0.10 g catalyst, 6 mL H_2_O, 3 MPa O_2_, 180°C for 0.25 h. Reproduced with permission from Lu et al. (2016).

In 2016, Lu et al. found that a Keggin-type heteropoly acid catalyst H_5_PV_2_Mo_10_O_40_ with H_2_SO_4_ was efficient for catalytic oxidation of cellulose to formic acid with oxygen as an oxidant (Lu et al., 2016). The authors discussed the effects of the pH on the catalyst and reaction pathway by adding H_2_SO_4_ with variable amounts (Table 5). It was shown in Figure 7 that when 1.6 wt% H_2_SO_4_ was added (equivalent to a significant reduction in pH), the conversion of cellulose increased from 60 to 100%, and the yield of formic acid increased from 28 to 61% in a reaction time of 5 min. It can be concluded that adding H_2_SO_4_ to the solution promotes the protonation of H_5_PV_2_Mo_10_O_40_. Decreasing the pH from 1.87 to 0.56 is beneficial for the catalyst protonation and can increase the oxidation potential. In summary, the H_5_PV_2_Mo_10_O_40_ + H_2_SO_4_ system shows a stronger catalytic impact by reducing pH than the direct use of H_5_PV_2_Mo_10_O_40_ (Lu et al., 2016).

**Table 5:** Synthesis of formic acid (FA) from cellulose at different pH (Lu et al., 2016).

**Catalyst**	**Temp/^**°**^C**	**Time/h**	**pH**	**FA yield/%**
HPA	90	66	1.79	9
HPA + TSA	90	66	0.91	22
HPA	170	9	1.96	3
HPA + HCl	170	9	1.87	34
HPA	180	3	1.80	45
HPA	180	1/12	1.79	28
HPA + H_2_SO_4_	180	1/12	0.56	61

*HPA, H_5_PV_2_Mo_10_O_40_; TSA, p-toluenesulfonic acid*.

**Scheme 12:**
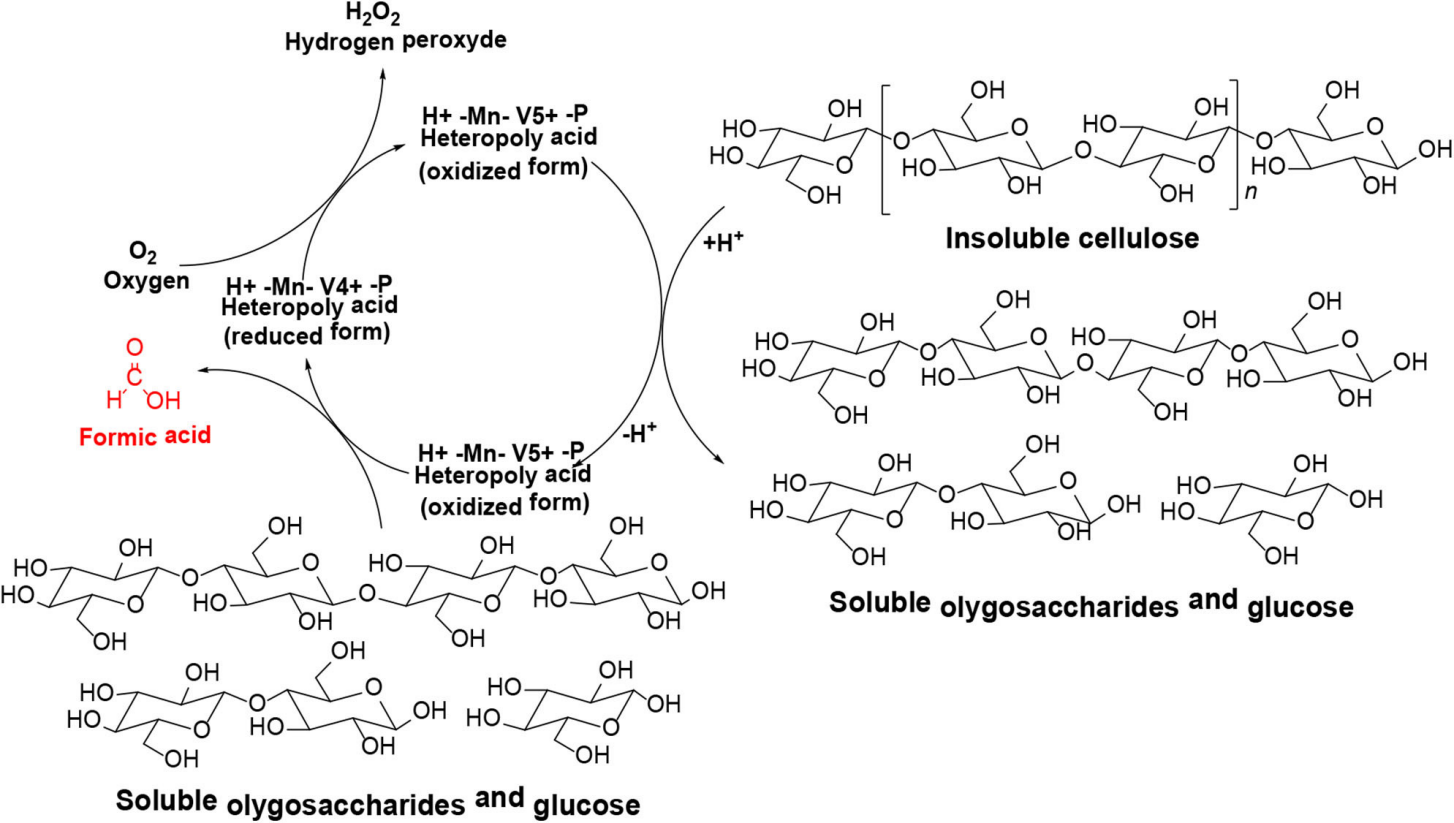
The overall scheme of the hydrolysis-oxidation of cellulose.

Likewise, Gromov et al. reported the one-pot catalytic process which was applied to hydrolytic oxidation of cellulose to formic acid (Gromov et al., 2016). In their work, Mo-V-P heteropoly acid catalysts were found to be beneficial for hydrolytic oxidation of cellulose to formic acid because of their bifunctional (i.e., oxidizing and acidic) catalytic properties. The oxidizing and acidic catalytic properties correspond to the conversion process of cellulose to formic acid which involves two stages: Step 1: hydrolysis of cellulose to glucose via acidic HPA sites; Step 2: The oxidation of glucose to formic acid taking place at the oxidation sites of Mo-V-P HPA (V^5+^) (Scheme 12). Therefore, cellulose was hydrolyzed and then oxidized to formic acid using the low-cost Mo-V-P HPA catalysts. Cascade hydrolysis and oxidation of cellulose to formic acid with high yields (65–66 mol%) were achieved by the single-step catalytic process in the presence of Mo-V-P HPA catalysts. In addition, the temperature of 150–160°C and air pressure of 10–20 Mpa (20% O_2_ and 80% N_2_) were found to be the optimal reaction conditions.

The Keggin-type (Mo-V-P)-HPAs were used to catalyze the hydrolysis of hemicellulose in a diluted aqueous solution that was reported by Shatalov (2019). HPAs of series H_(n+3)_[PMo_(12−n)_VnO_40_] used as the bifunctional catalysts were studied. For example (Mo-V-P)-HPAs-catalyzed hydrolysis of model xylan, which confirmed the established order of HPAs catalytic activity in hydrolysis reaction (H_3_PMo_12_O_40_ > H_6_[PMo_8.9_V_3.1_O_40_] > H_7_[PMo_7.9_V_4.2_O_40_] >> H_2_SO_4_). In addition (Mo-V-P)-HPAs, showed remarkable activity in both the acid-catalyzed reactions and the oxidative degradation of lignin. Therefore (Mo-V-P)-HPAs, as the green bifunctional catalysts have the potential to be an alternative to common mineral acids for selective hydrolysis and oxidative degradation of a variety of polysaccharides.

#### Cellulose Conversion to Other Substances

**Scheme 13:**
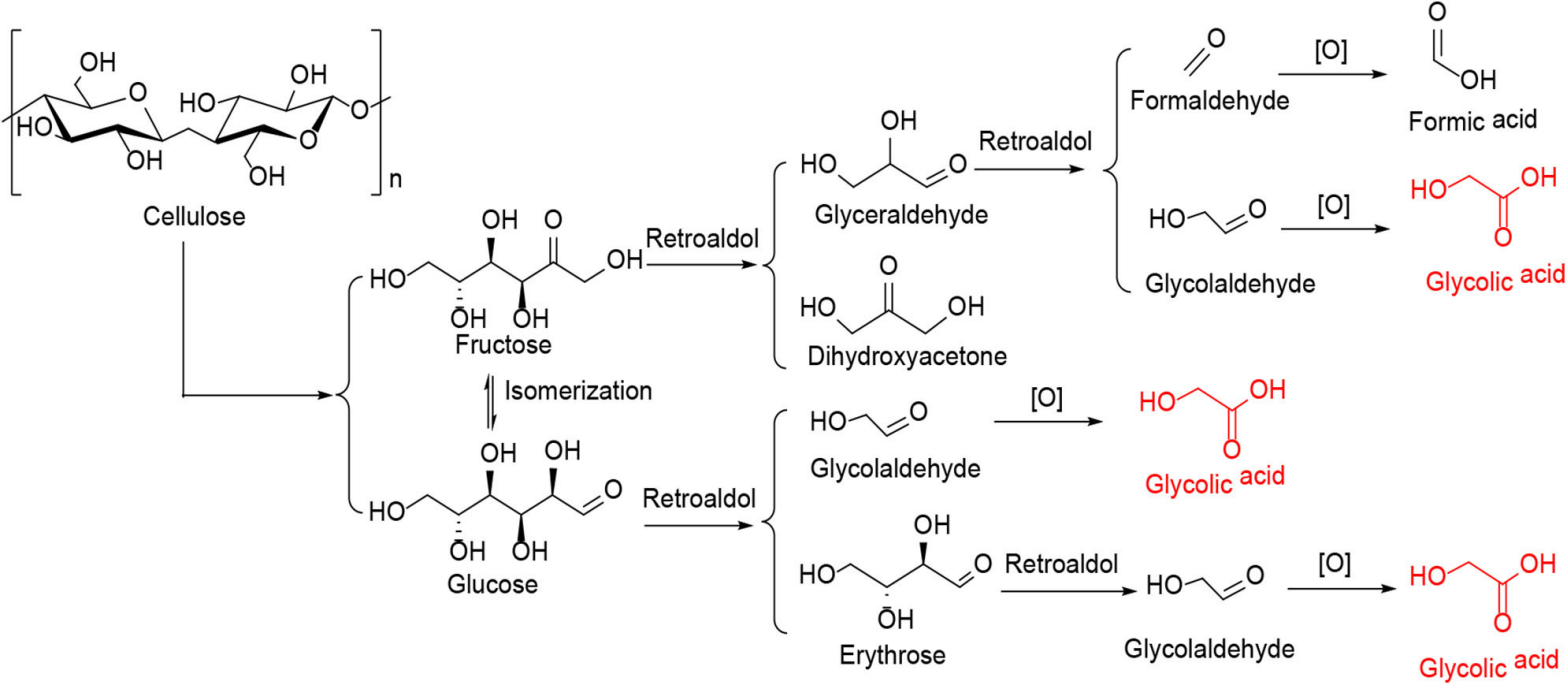
The proposed reaction pathways for the conversion of cellulose to glycolic acid.

**Figure 8:**
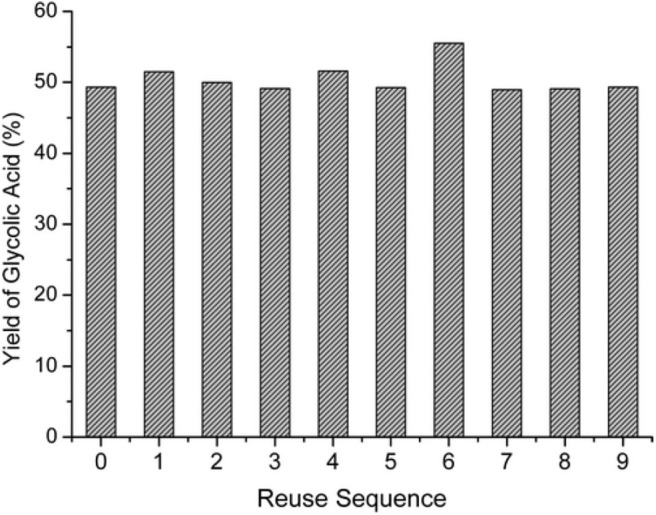
The yield of glycolic acid in the continuous reaction of cellulose conversion with HPM (H_3_PMo_12_O_40_) as the catalyst. Reproduced with permission from Zhang et al. (2012). Reaction conditions: 200 mg cellulose, 0.025 mmol catalyst, 20 mL H_2_O, 0.6 MPa O_2_, 180°C for 1 h. Reproduced with permission from Zhang et al. (2012).

**Figure 9:**
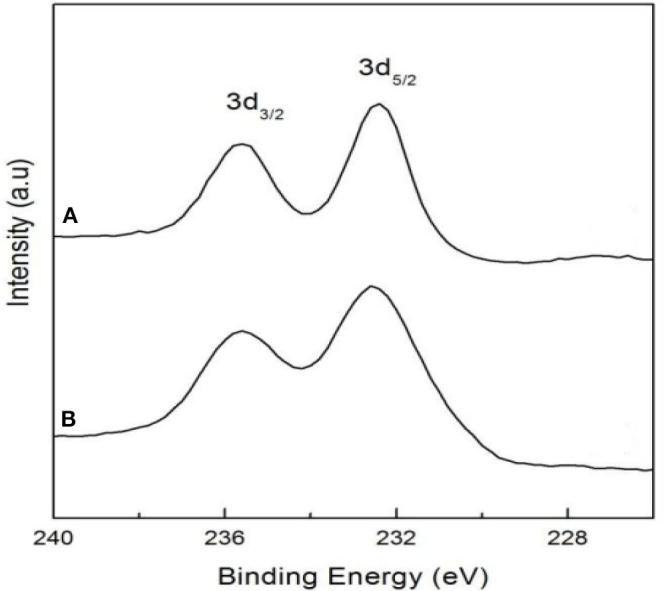
XPS spectra of Mo 3d in the fresh **(A)** and used **(B)** HPM (H_3_PMo_12_O_40_) catalyst. Reproduced with permission from Zhang et al. (2012).

In the oxygen environment, HPAs can also catalyze cellulose being converted to glycolic acid. In 2019, Zhang et al. reported the conversion of cellulose to glycolic acid by phosphomolybdic acid catalyst (Zhang et al., 2012). In this reaction system, the heteromolybdic acid acted as multifunctional catalysts that first hydrolyzed cellulose to glucose, followed by oxidation to glycolic acid in a water medium, which combines the advantages of homogeneous and heterogeneous catalysts. The proposed reaction pathways for the conversion of cellulose to glycolic acid are shown in Scheme 13. Cellulose was hydrolyzed to glucose, which was converted to glycolaldehyde by the continuous retro-aldol reaction, and then to glyoxal by oxidation. Simultaneously, the isomerization of glucose produced fructose, which was converted to glycolaldehyde and formaldehyde via the retro-aldol reaction, which was then oxidized to glycolic acid and formic acid, respectively. In addition, the authors investigated the reusability of the phosphomolybdic acid catalyst in cellulose conversion (Figure 8). The results indicated that the catalyst exhibited constant catalytic performance. X-ray photoelectron spectroscopy (XPS) showed that the oxidation state of Mo in the heteromolybdic acid catalyst was unchanged after successive reactions (Figure 9). It can be concluded that Mo-containing HPAs can effectively promote the conversion of various kinds of cellulosic biomass materials to glycolic acid.

**Figure 10:**
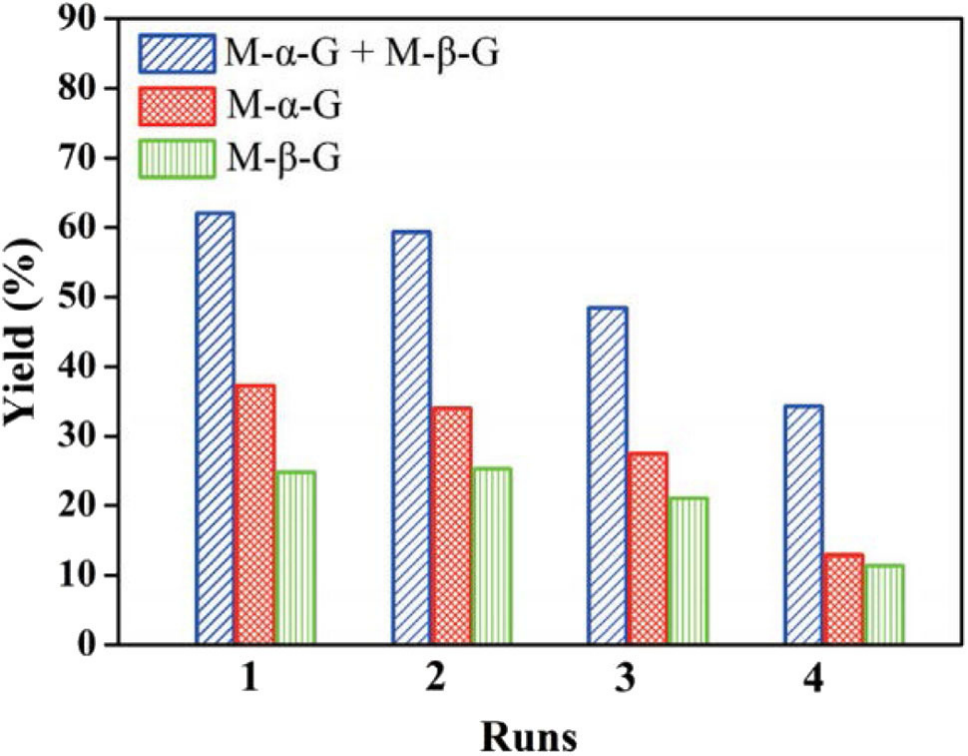
Recyclability of H_3_PW_12_O_40_ in alcoholysis of cellulose to methyl α-D-glucoside (M-α-G) and methyl β-D-glucoside (M-β-G). Reaction conditions: 0.3 g cellulose, 8.7 mmol/L catalyst H_3_PW_12_O_40_, 170°C for 2.75 h. Reproduced with permission from Zheng et al. (2018).

The effective alcoholysis of cellulose to monosaccharide is of great significance for fuel production. The H_3_PW_12_O_40_ catalyst can promote cellulose transformation into methyl glucosides after pretreatment of microcrystalline cellulose by the ionic liquid 1-allyl-3-methylimidazolium chloride, as reported by Zheng et al. (2018). In their work, cellulose was firstly obtained by pretreatment with the ionic liquid 1-allyl-3-methylimidazolium chloride, followed by cellulose alcoholysis using H_3_PW_12_O_40_ as the bifunctional catalyst. It was found that cellulose was easier to be saccharified after pretreatment by the ionic liquid. Meanwhile, prolonging the pretreatment time with the increased temperature is beneficial to the succedent alcoholysis reaction. Whereafter, the authors investigated the effects of various parameters on the alcoholysis reaction, including the alcoholizing time, the alcoholizing temperature, and different concentrations of H_3_PW_12_O_40_ catalyst. It was observed that under the optimum conditions (110°C, 60 min), the yield of methyl glucosides could be up to 70.2% with this system. Therefore, using some processes such as pretreatment of cellulose with ionic liquid can make it easier to saccharify, thus better-promoting HPA catalytic conversion of cellulose into the desired products. However, the reusability of the catalyst is not satisfactory. As shown in Figure 10, the activity of the H_3_PW_12_O_40_ catalyst decreased gradually in the repeated experiment, indicating the low catalyst stability.

In addition, the HPA is also an important catalyst for the formation of glucose from other saccharides. In 2015, Klein et al. found that HPA can catalyze the hydrolysis of glycogen to glucose (Klein et al., 2015). Through a series of experiments, it can be concluded that glycogen could be completely converted to glucose by using H_3_PW_12_O_40_·*n*H_2_O and H_4_SiW_12_O_40_·*n*H_2_O as the catalyst, respectively, and the optimized hydrothermal conditions are a mass fraction of catalyst 2.4%, 100°C temperature, and 2 h reaction time. In addition to hydrolysis in an autoclave, the authors also investigated microwave irradiation and sonication heating mode for glucose synthesis (Table 6). The results showed that using H_4_SiW_12_O_40_ as a catalyst, glycogen could be completely converted into glucose by the autoclave method and sonication method without other by-products, so relatively high selectivity toward glucose (>99%) was obtained using these two methods. On the other hand, when glycogen was hydrolyzed by microwave method, glycogen was completely transformed in 0.25 h, but giving by-products like levulinic acid and formic acid, which showed lower selectivity of glucose than the other two methods. More importantly, under microwave irradiation, the formation of glucose was the result of the increase of reaction temperature in the process of microwave irradiation (Klein et al., 2012). In the process of sonochemical irradiation, the acoustic bubble collapsed at a faster speed, causing local high temperatures, thus promoting the hydrolysis of glycogen to glucose (Klein et al., 2012). Therefore, high temperature is the key factor of glycogen hydrolysis, and the HPAs are highly efficient, environmentally friendly, and reusable catalysts that can catalyze the hydrolysis of glycogen to glucose.

**Table 6:** Effect of H_4_SiW_12_O_40_ on glycogen hydrolysis using different methods (Klein et al., 2012).

**Method**	**Reactant**	**Reaction products**		
	**Glycogen**	**Glucose**	**LA^***a***^**	**FA^***b***^**
Autoclave^c^	–^f^	+^g^	–	–
Microwave^d^	–	+	+	+
Sonication^e^	–	+	–	–

a LA, levulinic acid.

b FA, formic acid.

c Glycogen: 50 mg, HSiW: 50 mg, water: 2 mL, 2 h, 100°C.

d Glycogen: 200 mg, HSiW: 1.2 g, water: 10 mL, 0.25 h.

e Glycogen: 250 mg, HSiW: 1.5 g, water: 10 mL, 3 h.

f -, absent.

g *+, present*.

### Substituted HPAs

Substituted HPAs are formed when H^+^ ions on conventional HPSs are substituted by monovalent cations, such as Cs^+^ ions. One of the typical substitutional HPAs, Cs_*x*_H_3−x_PW_12_O_40_ has the advantages of super acidity, microporous structure, shape selectivity, and hydrophobicity, which is conducive to the hydrolysis of cellulose (Okuhara, 2002).

#### Cellulose Conversion to Glucose

**Scheme 14:**
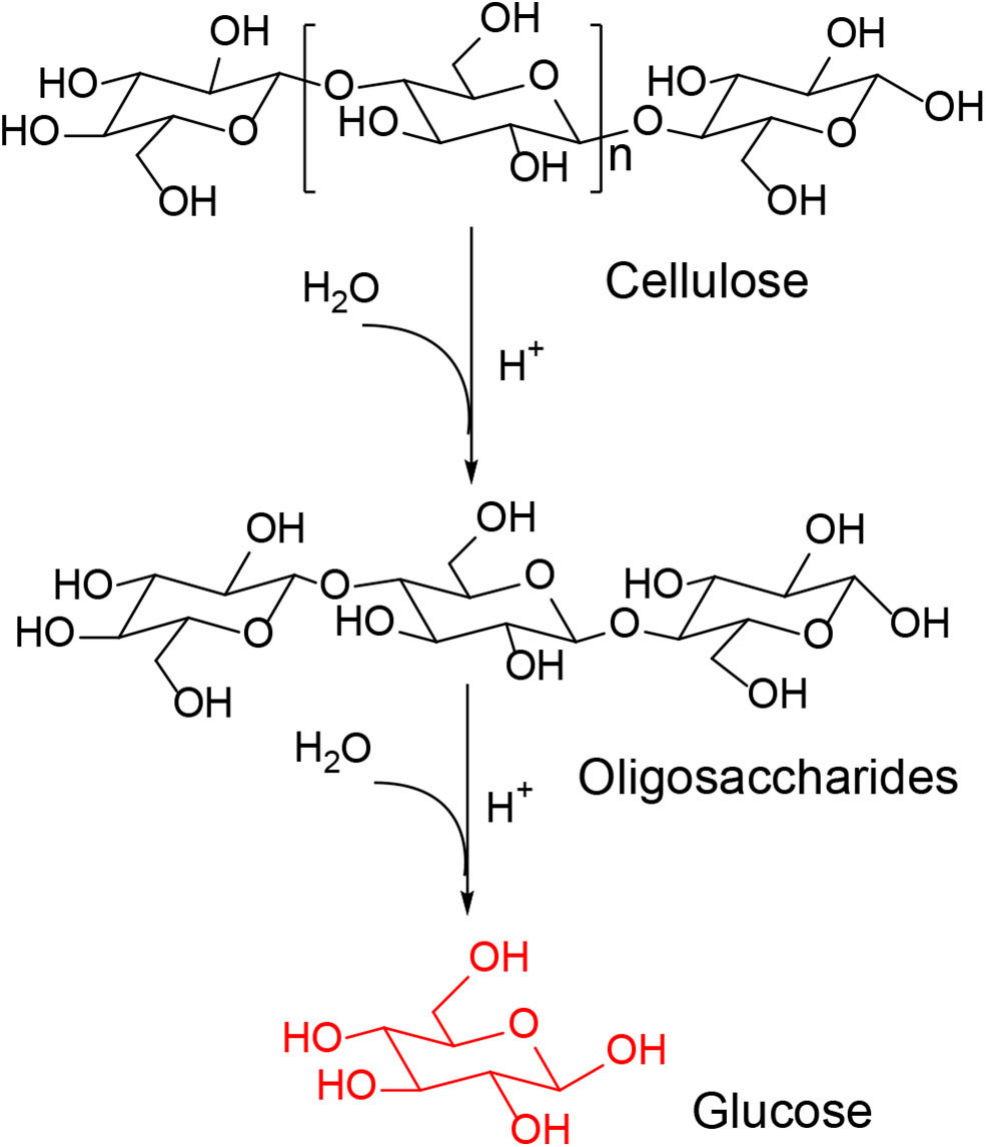
The pathways of cellulose hydrolysis into glucose over Cs_*x*_H_3−x_PW_12_O_40_.

In 2010, Tian et al. reported hydrolysis of cellulose over the Cs_*x*_H_3−x_PW_12_O_40_ (*x* = 1–3) catalysts, which were active in the hydrolysis of cellulose into glucose. Notably, cellulose can be hydrolyzed to glucose in the presence of Cs_*x*_H_3−x_PW_12_O_40_ catalysts due to the breaking of β-1,4-glycoside bond (Scheme 14).

**Figure 11:**
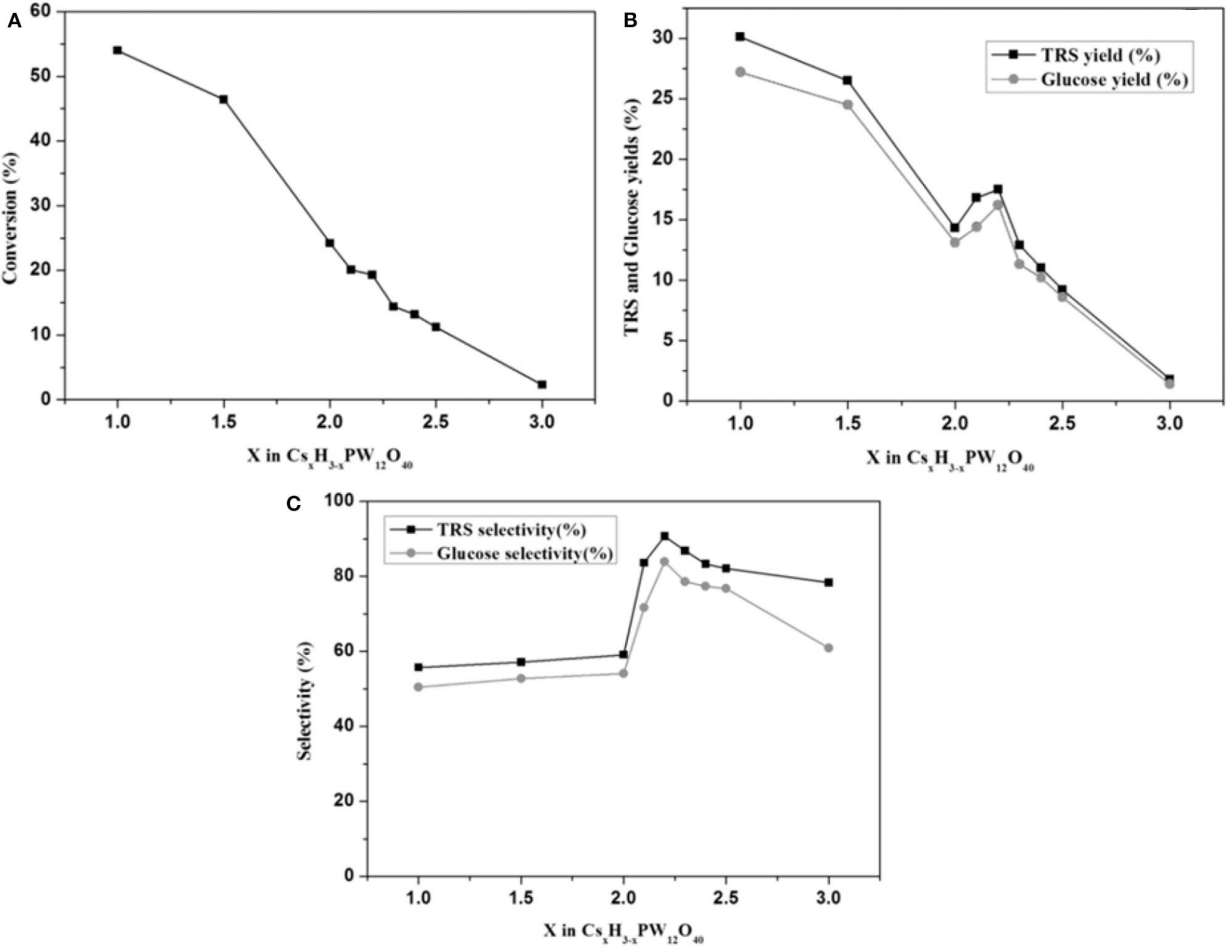
**(A)** The relationship between cellulose conversion and cesium content in Cs_*x*_H_3−x_PW_12_O_40_, **(B)** the relationship between the yield of TRS and glucose and cesium content in Cs_*x*_H_3−x_PW_12_O_40_, and **(C)** the relationship between the selectivity of TRS and glucose and cesium content in Cs_*x*_H_3−x_PW_12_O_40_. Reaction conditions: 0.1 g microcrystalline cellulose, 0.06 mmol Cs_*x*_H_3−−x_PW_12_O_40_, 5 mL distilled water, at 160°C for 6 h (stirring 30 rpm). Reproduced with permission from (Okuhara, 2002).

After a series of catalyst tests, it was found that Cs_1_H_2_PW_12_O_40_ showed the best catalytic performance in terms of cellulose conversion (54.0%) and glucose yield (27.2%), while Cs_2.2_H_0.8_PW_12_O_40_ showed the highest selectivity toward glucose (83.9%). The test results are shown in Figures 11A–C. In the meantime, they also studied the effect of Cs_*x*_H_3−x_PW_12_O_40_'s properties on cellulose hydrolysis. Cs_1_H_2_PW_12_O_40_ with the strongest protonic acid site was found to have the best catalytic performance for the hydrolysis and transformation of cellulose. However, the catalyst surface area and porous structure have little effect on the conversion of cellulose (Tian et al., 2010). Stronger Brønsted acid is more beneficial to the hydrolysis of β-1,4-glycoside bond in cellulose (Furukawa et al., 2009). It is further proved that Cs_1_H_2_PW_12_O_40_ with the strongest acidity is more conducive to the degradation of cellulose. When the catalyst and the unreacted cellulose were in the same reaction, the catalyst kept high activity during the hydrolysis of cellulose to TRS and glucose with the yields was 30.1 and 27.2%, respectively. Therefore, the tested acid catalyst is stable and can be reused.

#### Cellulose Conversion to Other Products

In 2011, Geboers et al. studied the hydrotreated cesium salts of HPAs in combination with Ru/C which can be used to degrade cellulose for the synthesis of hexitol (Geboers et al., 2011). The authors discussed the effects of calcination temperature and cesium HPAs hydrotreating conditions on cellulose conversion. The catalytic properties of several cesium HPAs were discussed and compared with conventional HPAs (Table 7). The synthesized cesium salts of HPAs were more active than the natural HPA salts, and their hydrolysis activity was further improved by increasing the calcination and hydrotreating temperature in the synthesis process.

**Table 7:** Hydrolysis and hydrogenation of cellulose with Ru/C and different HPAs at 190°C and 5 MPa H_2_ (Geboers et al., 2011).

**Entry**	**Acid catalyst**	**Reaction time/h**	**Conversion (%)**	**Hexitol yield**
1	Cs_3.5_SiW${\;}_{300}^{\text{a}}$	24	100	55
2	Cs_3.5_SiW${\;}_{600}^{\text{a}}$	13	100	56
3	Cs_2.5_PW${\;}_{300}^{\text{a}}$	8	91	45
4	Cs_2.5_PW${\;}_{600}^{\text{a}}$	8	95	59
5	Cs_2.5_${\text{PW}}_{600}^{\text{b}}$	11	93	46
6	H_4_SiW^c^	24	100	18
7	H_3_PW^c^	24	81	41

a 1 g cellulose, 0.5 g CsHPA ([H+] = 1.5 mM), 0.25 g Ru/C, 50 m water.

b 5 g cellulose, 2.5 g CsHPA ([H+] = 7.5 mM), Ru/C = 1.25 g, water 50 mL.

c *Adjust the HPA amount to make the proton concentration consistent with CsHPA*.

As compared with their fully protonated counterparts (Cs_2.5_PW and Ru/C or Cs_3.5_SiW and Ru/C), the cesium HPAs have higher proton activity due to their higher surface acidity and hydrophobicity. After a hydrotreatment of cesium HPAs catalysts in water at 190°C was performed, the activity and selectivity of the catalysts were improved obviously due to the increase of crystallinity and hydrophobicity of samples. Finally, they found that these cesium HPAs could be completely recovered under certain conditions by a simple recrystallization method (Geboers et al., 2011). In 2011, they had studied the catalysts formed by natural HPAs in combination with Ru/C for cellulose degradation to hexitols (90%). These catalysts also had extraordinary catalytic performance, but they were difficult to recovery and reuse because of the limitation of reaction temperature (Geboers et al., 2010). Therefore, the hydrotreated cesium salts of HPAs in combination with Ru/C are more ideal for cellulose degradation.

**Figure 12:**
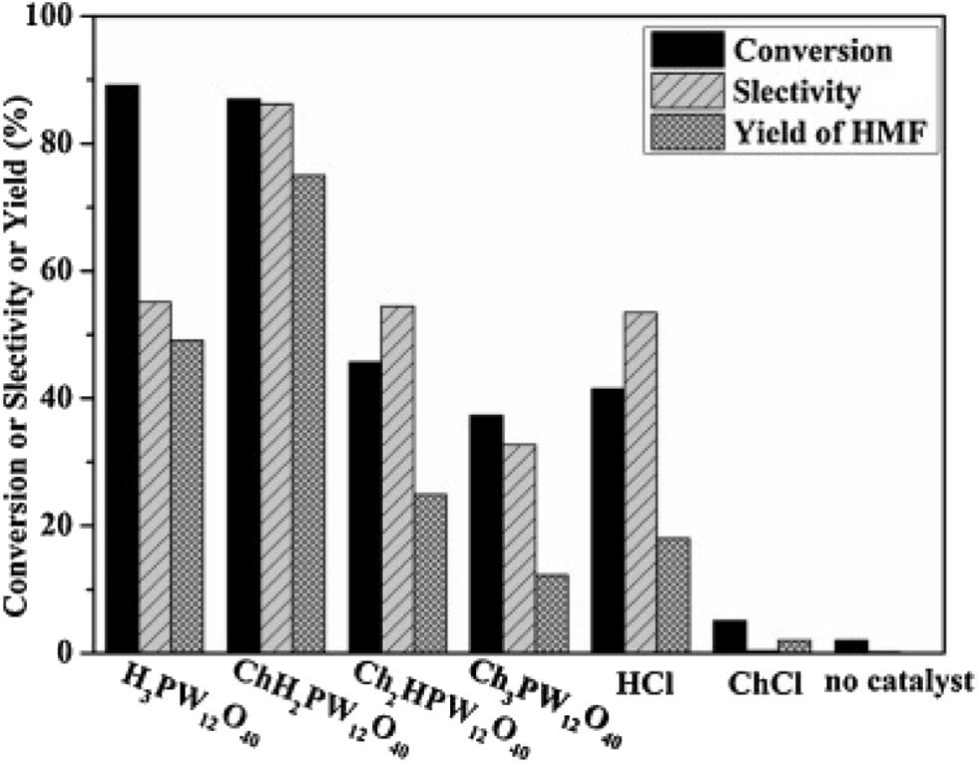
Hydrolysis of cellulose under different catalysts. Reaction conditions: 0.1 g cellulose, 0.11 mmol catalyst, 0.5 ml water, 5 mL methyl isobutyl ketone, 140°C for 8 h. Reproduced with permission from Zhang et al. (2016).

**Figure 13:**
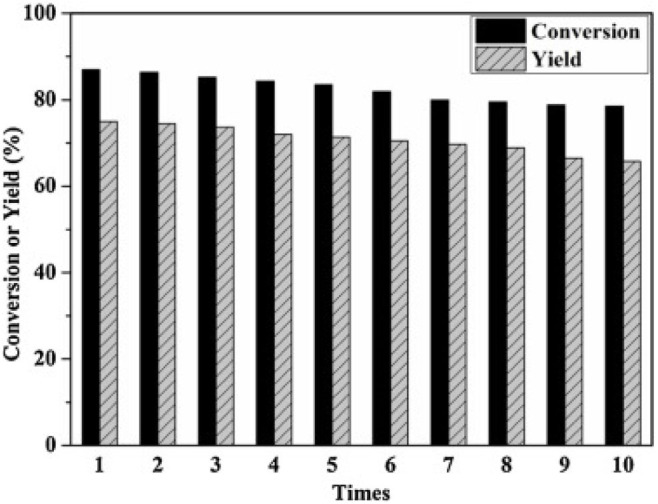
The recycle reaction test of catalyst. Reaction conditions: 0.1 g cellulose, 0.11 mmol ChH_2_PW_12_O_40_, 0.5 mL water, 5 ml methyl isobutyl ketone, 140°C for 8 h. Reproduced with permission from Zhang et al. (2016).

In 2014, Zhang et al. synthesized a series of HPA catalysts (HOCH_2_CH_2_N(CH_3_)_3_)_*x*_H_3x_PW_12_O_40_ (HOCH_2_CH_2_N(CH_3_)_3_ abbreviated as Ch), which could be used as heterogeneous catalysts for degradation of cellulose to 5-hydroxymethylfurfural (HMF) (Zhang et al., 2016). By comparing several different types of acid catalysts (Figure 12), they found that the conversion of cellulose with Brønsted acid in HCl and H_3_PW_12_O_40_ was 41.5 and 89.2%, respectively, while that of ChCl and Ch_3_PW_12_O_40_ without Brønsted acid was 2.0 and 12.2%, respectively. So the strong Brønsted acidity of the catalyst was the necessary condition for cellulose hydrolysis. And (HOCH_2_CH_2_N(CH_3_)_2_)H_2_PW_12_O_40_ had the highest catalytic activity among the previous catalysts. The yield of HMF can reach 75% when cellulose was catalyzed by (HOCH_2_CH_2_N(CH_3_)_2_)H_2_PW_12_O_40_ within 8 h at 140°C. Besides, the temperature-responsive property and high stability of the catalyst made the recovery and reuse benefit reach 10 times without obvious activity loss (Figure 13).

Overall, it can be seen that the substituted HPA catalysts showed high activity in the conversion of cellulose and they were stable and reusable. Therefore, the substituted HPA catalysts have good application prospects in cellulose degradation. However, the substituted HPA catalysts still suffer the disadvantage of low target product yield or selectivity, possibly due to the resistance of mass transfer.

### Supported HPAs

So far, more and more attention has been paid to the supported acid catalysts, especially the supported HPAs. The dispersion of these catalysts on the support material increases the specific surface area, thus improving the catalytic activity. Some supported HPAs have been used in the degradation of cellulose. In 1981, Yusuke and Urabe found that activated carbon can encapsulate a certain amount of HPA, thus producing solid acid catalysts, which provided a convenient method for liquid phase etherification and gas-phase selective esterification of alcohols (Yusuke and Urabe, 1981). In 1989, the nuclear magnetic resonance (NMR) technology had been used in the structural research of HPAs and H_3_PW_12_O_40_ supported on SiO_2_ (Mastikhin et al., 1990).

**Scheme 15:**
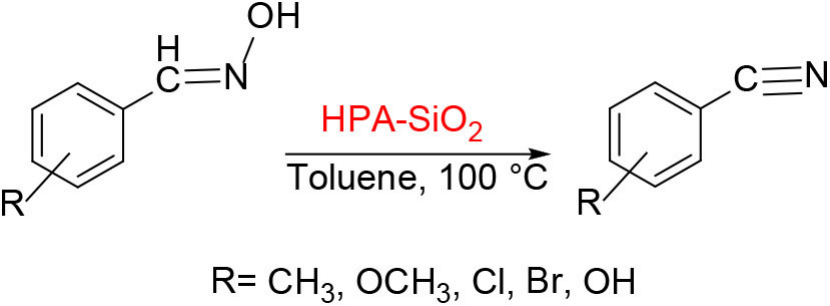
Dehydration of aldehydes to nitriles catalyzed by HPA-SiO_2_.

HPAs are usually used as soluble catalysts in the liquid phase and as supported catalysts in the gas phase. From a practical point of view, it is useful to develop those supported HPA catalysts, which can be applied to a variety of reactions through fixed catalysts without leakage of HPA in the liquid, such as acylation of anisole with acetic anhydride (Bachillerbaeza and Anderson, 2004), the electrophilic substitution of phenols and aldehydes with HPA/MCM-41 (Udayakumar et al., 2007), and dehydration of aldehydes to nitriles catalyzed by silica-supported HPAs (Scheme 15; Parghi et al., 2011), and so on.

**Figure 14:**
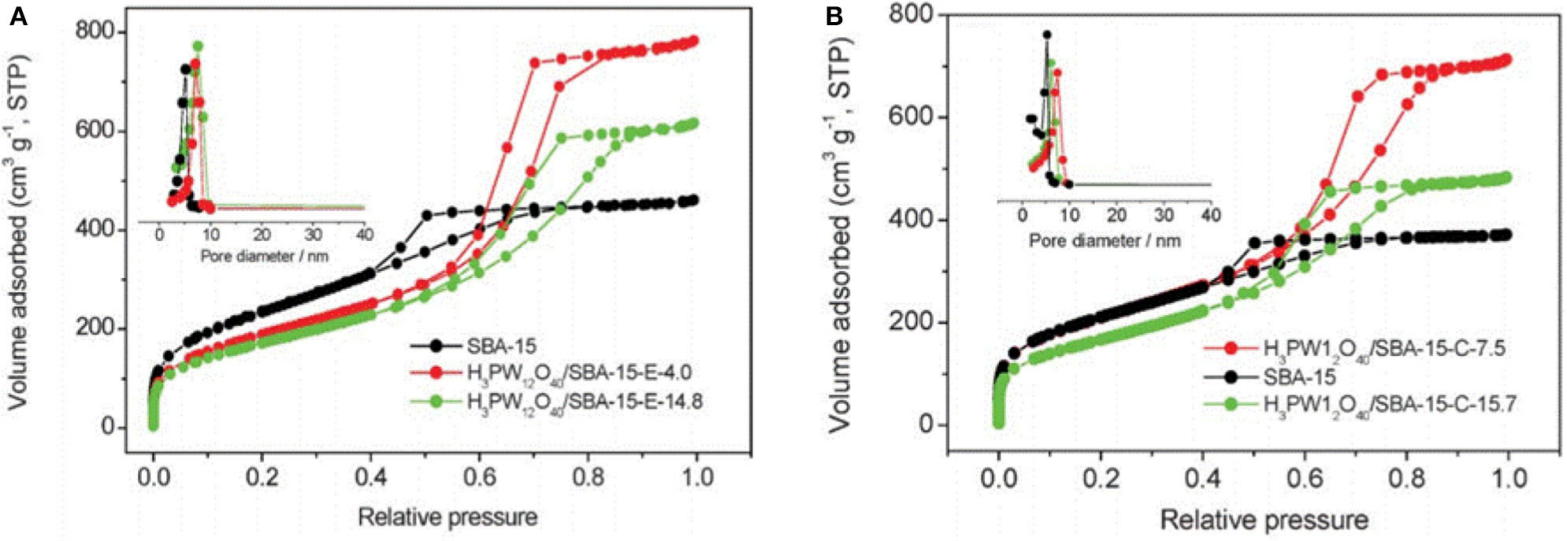
Nitrogen adsorption-desorption isotherms and pore size distribution profiles (inset) of H_3_PW_12_O_40_/SBA-15 materials. **(A)** Removal of H_3_PW_12_O_40_/SBA-15 by extraction with acidic ethanol, and **(B)** removal of H_3_PW_12_O_40_/SBA-15 by calcination at 400°C. Reproduced with permission from Guo et al. (2007).

In 2007, Guo et al. studied a sol-gel co-condensation technology that can be used to prepare the green supported heteropoly acid catalysts (H_3_PW_12_O_40_-silica), which was an efficient and reusable solid acid catalyst for the catalytic esterification of levulinic acid (LA) with phenol (Guo et al., 2007). The SBA-15 silica-supported H_3_PW_12_O_40_ catalysts exhibited unique surface physicochemical properties, such as high porosity, larger pore size, and larger surface area (Table 8) that is attractive for the study of acid-catalyzed reactions. Figure 14 shows the nitrogen adsorption-desorption isotherms of the H_3_PW_12_O_40_/SBA-15 catalyst, indicating a clear mesoporous structure and relatively narrow pore size distribution. It is shown in Figure 14 that all isotherms are type IV and the results show that the capillary condensation occurs at high relative pressure (*P*/*P*_0_ = 0.45−0.85), indicating that the mesoporous structure is clear and the pore size distribution is relatively good.

**Table 8:** Comparison of surface properties and catalytic activities of different catalysts in the esterification of levulinic acid (LA) with phenol (Guo et al., 2007).

**Catalyst**	**2θ/^***o***^**	**S_***BET***_/m^**2**^ g^**−1**^**	**D${\;}_{P}^{a}$/nm**	**Porosity^***b***^**	**LA conversion (%)**	**TOF**
H_3_PW_12_O_40_/SBA-15-E-4.0	0.95	691.6	7.1	1.20	23.6	6.9
H_3_PW_12_O_40_/SBA-15-E-14.8	0.92	683.0	7.4	0.95	74.8	46.4
H_3_PW_12_O_40_/SBA-15-E-17.5	0.90	630.4	8.6	0.91	80.1	51.0
H_3_PW_12_O_4_/SBA-15-C-7.5	0.95	753.0	6.0	1.10	19.2	3.5
H_3_PW_12_O_4_/SBA-15-C-11.3	0.91	713.2	6.4	0.85	72.3	48.5
H_3_PW_12_O_4_/SBA-15-C-15.7	0.96	604.5	6.6	0.75	80.3	53.9
H_3_PW_12_O_40_/SiO2-D-15.4	–	317.6	1.2	0.34	5.3	0.65
H_3_PW_12_O_40_	–	5.5	–	–	60.1	1.04
HCl	–	–	–	–	65.4	0.73
SBA-15-E	0.99	858.2	5.2	0.71	0	0
SBA-15-C	0.96	750.1	5.3	0.51	0	0

^a^Pore diameters are estimated from BJH desorption determination.

^b^The porosity is estimated from the pore volume determined using the adsorption branch of the N_2_ isotherm curve.

*Reaction conditions: 3.4 mmol LA, 13.6 mmol phenol, 0.05 g catalyst, 100°C for 8 h*.

In 2011, silica-supported HPA, a heterogeneous and environmentally benign catalyst was found by Parghi et al. (2011). And it was characterized by many analytical techniques. The effect of catalysts on aldosterone dehydration is shown in Table 9. The results showed that the silica-supported phosphotungstic acid catalyst was the most favorable catalyst for the aldosterone dehydration reaction they studied. The reusability of the catalyst was investigated, and it was found that the catalyst was stable in five consecutive recycles with benzonitrile yield slightly decreasing from 85 to 79%.

**Table 9:** Aldosterone dehydration with different catalysts (Parghi et al., 2011).

**Entry**	**Catalyst**	**Benzonitrile yield/%**
1	SiO_2_	10
2	HPW	51
3	HPW	40
4	HPW-SiO_2_	85
5	HPW-SiO_2_	72
6	–	–

*Reaction condition: 2 mmol benzaldehyde oxime, catalyst (20 wt%); 3 mL toluene, 100°C for 4.5 h*.

**Scheme 16:**
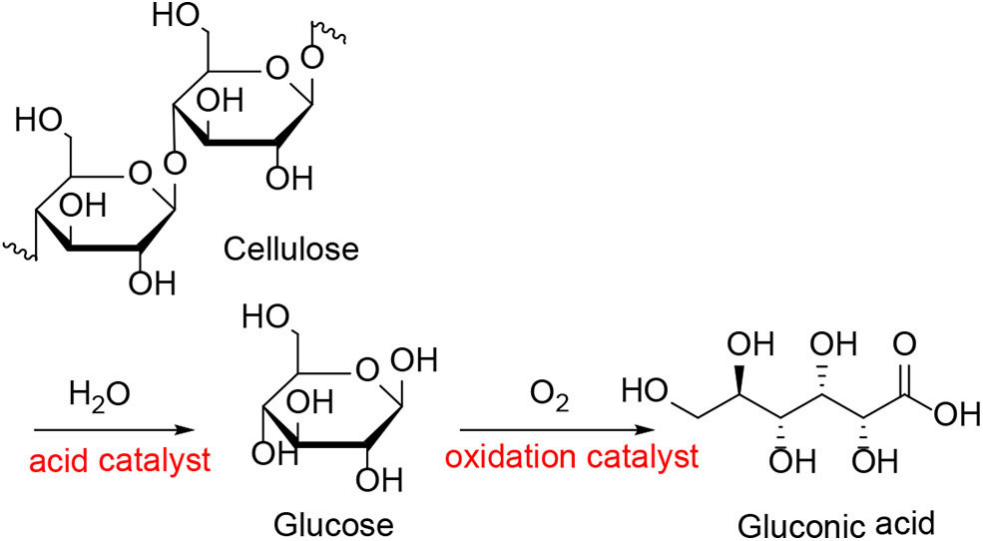
Catalytic conversion of cellulose to gluconic acid with Au/Cs_*x*_H_3x_PW_12_O_40_.

**Figure 15:**
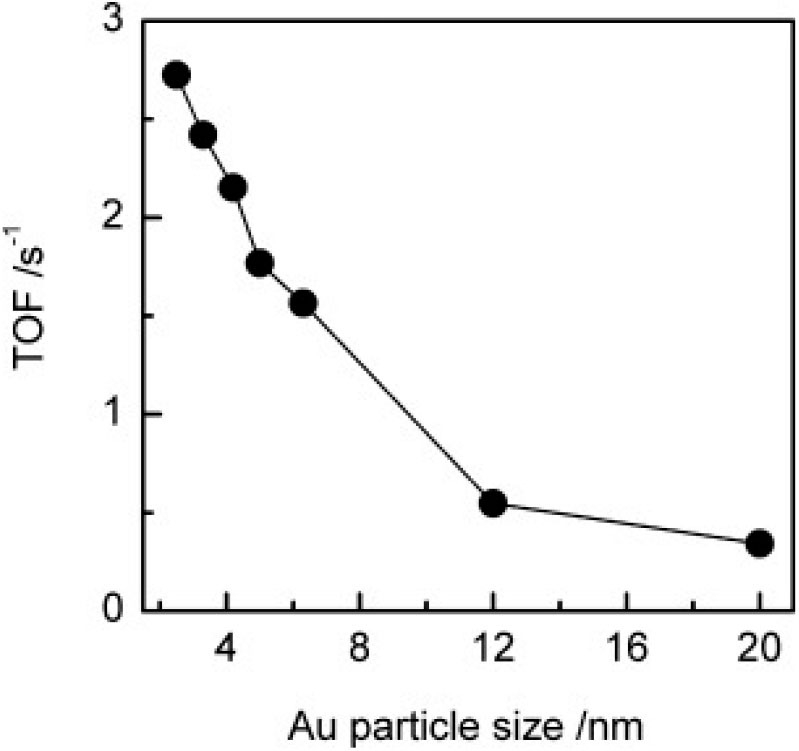
The effect of Au/Cs_2.2_H_0.8_PW_12_O_40_ catalysts with different average particle sizes of Au on TOFs of glucose oxidation to gluconic acid. Reaction conditions: 24 mmol/L glucose, 25 mL H_2_O, 0.5 MPa O_2_, 145°C for 3 h. Reproduced with permission from An et al. (2012).

In 2012, An et al. reported on the conversion of cellulose and cellobiose by polyoxometalate-supported gold nanoparticles (An et al., 2012). The catalysts, Cs_*x*_H_3x_PW_12_O_40_ supported Au nanoparticles can convert cellulose and cellobiose into gluconic acid that is widely used in the pharmaceutical and food industry. The reaction pathways of cellulose to gluconic acid are shown in Scheme 16. Cellulose was first hydrolyzed to glucose, which was then oxidized to gluconic acid by oxygen. The selectivity of Au/Cs_x_H_3x_PW_12_O_40_ to gluconic acid was significantly higher than that of Au catalysts supported on typical metal oxides, carbon nanotubes, and zeolites (Table 10; An et al., 2012). The possible reasons were the acidity of polyoxometalates and the average particle size of Au nanoparticles. The strong acidity of polyoxometalates not only benefited the conversion of cellobiose but also promoted desorption to improve the selectivity of gluconic acid. And smaller Au nanoparticles expedited the oxidation of glucose to gluconic acid, thus increasing the conversion of cellobiose (Figure 15).

**Table 10:** Transformation of cellobiose with different supported catalysts (An et al., 2012).

**Catalyst**	**Conversion (%)**	**GA^***a***^ selectivity (%)**	**GA yield (%)**
Au/Cs_1.2_H_1.8_PW_12_O_40_	97	>99	97
Au/Cs_1.7_H_1.3_PW_12_O_40_	98	96	94
Au/Cs_2.2_H_0.8_PW_12_O_40_	96	95	91
Au/Cs_2.6_H_0.4_PW_12_O_40_	97	90	87
Au/Cs_3.0_PW_12_O_40_	95	85	81
Au/SiO_2_	67	61	41
Au/Al_2_O_3_	95	31	29
Au/TiO_2_	96	63	60
Au/H-ZSM-5	45	76	34
Au/HY	64	62	40
Au/CNT	84	86	72

*^a^GA, GLUCONIC acid. Reaction conditions: 12 mmol/L cellobiose, 0.050 g catalyst (Au loading: 1.0 wt%), 25 mL H_2_O, 0.5 MPa O_2_, 145°C for 3 h. Reproduced with permission from An et al. (2012)*.

**Figure 16:**
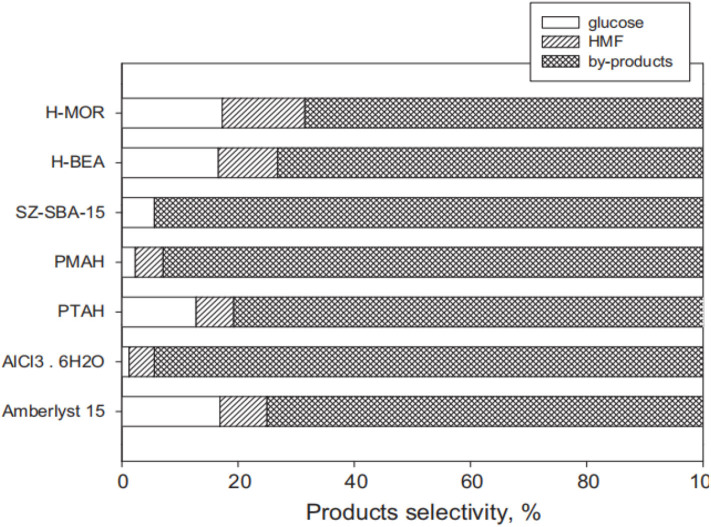
Activity comparison of different solid acid catalysts for cellulose conversion. Reaction conditions: 2 g cellulose, 0.2 g catalyst, 55 mL distilled water, 190°C for 5 h. Reproduced with permission from Lanzafame et al. (2012).

In 2012, Lanzafame et al. reported that some solid acid catalysts can convert cellulose into glucose, HMF, and other soluble by-products in 190°C aqueous solution for 5 h or <5 h (Lanzafame et al., 2012). They mainly studied supported solid acid catalysts, including sulphated zirconia supported over HPAs, which were found to effectively transform cellulose at a reaction temperature of 190°C (Figure 16). It can be seen that all the catalysts were active in the hydrolysis of cellulose to glucose. However, a significant disadvantage is that the conversion of cellulose with a solid acid catalyst produces more by-products, resulting in low glucose selectivity.

**Figure 17:**
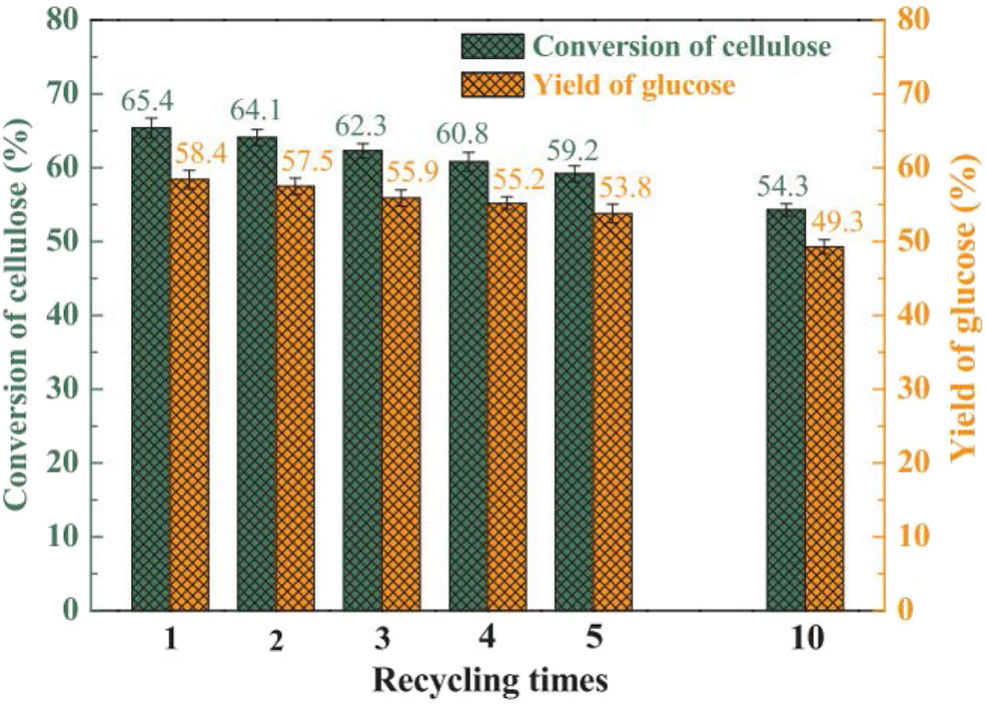
Reusability test of the HPW/CF30 catalyst for cellulose hydrolysis. Reaction conditions: 0.1 g MAMCC (mechanical activation microcrystalline cellulose), 1.0 g HPW/CF30, 10 mL H_2_O, 170°C for 9 h. Reproduced with permission from Zhang et al. (2019).

In 2014, Hafizi et al. found a unique heterogeneous silica-supported Preyssler heteropoly acid catalyst (H_14_NaP_5_W_30_O_120_), which can be used for alkylation of benzene (Hafizi et al., 2014). Preyssler HPA has the advantages of high thermal stability, high hydrolysis stability, regeneration, safety, easy separation, and so on (Bamoharram et al., 2004; Heravi et al., 2007, 2008). It combines with silica to form a supported heteropoly acid catalyst, which is active for the alkylation of benzene. In 2019, Zhang et al. developed a series of highly efficient heterogeneous catalysts with good stability and reusability for the catalytic conversion of cellulose to glucose. A typical Keggin type HPA (H_3_PW_12_O_40_) was immobilized on the surface and pore of carbon foam (CF) to prepare supported HPA catalyst (H_3_PW_12_O_40_/CF) for cellulose conversion to glucose (Zhang et al., 2019). After comparing with different CF*s* that were prepared from damaged starch and gluten protein (*s* is the percentage of gluten protein), it was found that CF30 was considered as the best porous support material for fixing the HPW catalyst because of its higher specific surface area (Table 11). In addition, H_3_PW_12_O_40_/CF30 catalyst had good reusability and was easy to separate and recover from the reaction system (Figure 17). It was shown that the supported catalyst could effectively catalyze the conversion of cellulose to glucose, and the supported technology could improve the catalytic performance and high-efficiency reusability of the catalyst.

**Table 11:** Selected properties of different catalysts (Zhang et al., 2019).

**Sample**	**Compressive strength (MPa)**	**Specific surface area (m^**2**^ g^**−1**^)**	**Pore volume (cm^**3**^ g^**−1**^)**	**Mean pore size (nm)**
CF0	–	201.2	0.208	9.72
CF10	0.096	225.6	0.221	7.95
CF20	0.126	253.9	0.232	4.36
CF30	0.147	302.5	0.259	4.03
CF40	0.146	255.2	0.242	3.65
Original HPW/CF30	0.148	200.9	0.222	3.73
Reused HPW/CF30	0.140	221.9	0.223	2.01

Based on the studies of these supported HPA catalysts, it can be seen that the supported HPAs have a higher specific surface area than conventional HPAs. Also, the supported HPAs can be used in the heterogeneous reaction, which is more convenient for product separation and can be reused. It is worth noting that there are still some shortcomings of the catalysts, such as low selectivity of the target product and gradual decline of stability of the catalysts in recycles.

### Assembled HPAs

**Figure 18:**
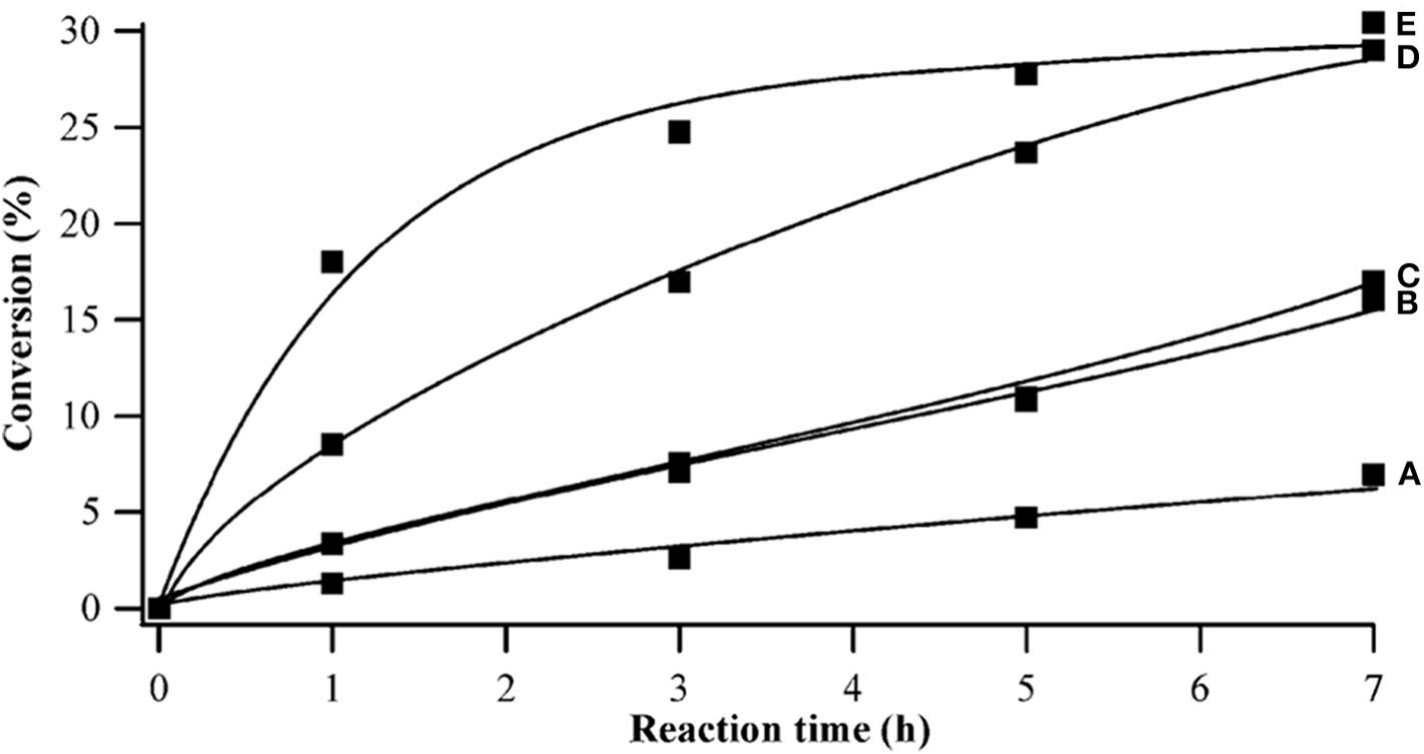
Effect of catalysts on conversion of acetic acid in esterification. Catalysts: **(A)** without catalyst, **(B)** micron-sized HPW/Cu_3_(BTC)_2_, **(C)** ultrastable Y zeolite (CBV 720), **(D)** 65 nm HPW/Cu_3_(BTC)_2_, and **(E)** 50 nm HPW/Cu_3_(BTC)_2._ Reaction conditions: 1 mol acetic acid, 40 mol 1-propanol, 2.23 wt% catalyst, 60°C. Reproduced with permission from Wee et al. (2010).

In recent years, people began to explore new efficient catalysts for cellulose conversion. The assembled HPA catalysts have also become one of the research directions. It is noteworthy that the assembled HPA catalysts were studied for the degradation of cellulose in biomass and other valuable catalytic reactions. For example, in 2010, Wee et al. proposed a simple and highly repetitive synthesis method for the assembly of nanomaterial catalyst H_3_PW_12_O_40_/Cu_3_(BTC)_2_ (BTC = benzene tricarboxylic acid). Cu_3_(BTC)_2_ was encapsulated into Keggin HPA H_3_PW_12_O_40_ for application in catalysis (Wee et al., 2010). And this assembled HPA nanomaterial catalyst can be used in acid-catalyzed esterification (Figure 18).

**Figure 19:**
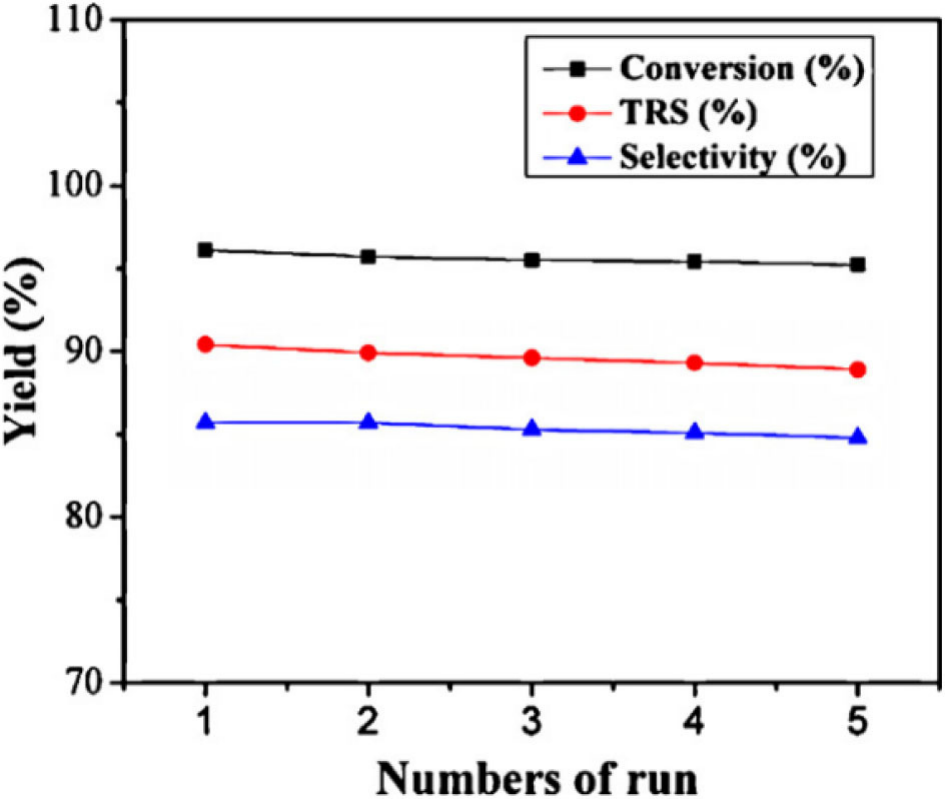
Recycling test of the [C_16_H_33_N(CH_3_)_3_]H_2_PW_12_O_40_ catalyst. Reaction conditions: 0.1 g cellulose, 0.07 mmol catalyst, 7 mL water, 170°C for 8 h. Reproduced with permission from Cheng et al. (2011).

In 2011, Cheng et al. reported that a micellar HPA catalyst [C_16_H_33_N(CH_3_)_3_]H_2_PW_12_O_40_ (C_16_H_2_PW) was designed to hydrolyze cellulose and starch into glucose. The micellar HPA catalyst can allow cellulose molecules to enter into the catalytic sites, so as to improve the reaction rate and cellulose conversion rate (Dwars et al., 2005; Cheng et al., 2011). As a heterogeneous catalyst [C_16_H_33_N(CH_3_)_3_]H_2_PW_12_O_40_ exhibited remarkable catalytic performance for cellulose degradation, and the yield and selectivity of glucose were 39.3 and 89.1% respectively (Table 12). In addition, the catalyst was easily recovered by centrifuge and had good reusability (Figure 19). Therefore, the heterogeneous micelle HPA catalyst is a clean, economical and environmentally friendly catalyst for hydrolysis of cellulose.

**Table 12:** Hydrolysis of cellulose over different catalysts (Cheng et al., 2011).

**Catalyst (mmol)**	**Reaction time (h)**	**Amount of water (mL)**	**Cellulose conversion (%)**	**TRS yield (%)**	**Glucose yield (%)**	**Glucose selectivity (%)**	**TOF (g mmol^**−1**^ h^**−1**^)**
CTAB (0.07)	8	7	0	0	0	0	0
H_3_PW_12_O_40_ (0.07)	5	5	70.4	68.1	59.9	85.1	0.201
Cs_2.5_H_0.5_PW (0.07)	6	5	23.7	22.2	21.3	89.9	0.056
C_16_ H_2_PW (0.08)	8	7	45.7	36.3	35.1	76.8	0.071
C_16_ H_2_PW (0.07)	8	8	43.7	35.7	34.2	78.3	0.078
C_16_ H_2_PW (0.07)	8	7	44.1	40.2	39.3	89.1	0.079
C_16_ H_2_PW (0.07)	8	6	42.6	40.5	37.0	86.9	0.076
C_16_ H_2_PW (0.07)	8	5	39.3	34.4	33.5	85.2	0.070
C_16_ H_2_PW (0.07)	8	4	34.7	30.7	29.3	84.4	0.062
C_16_ H_2_PW (0.06)	8	7	42.8	38.5	37.6	87.9	0.089
C_16_ H_2_PW (0.05)	8	7	37.4	31.3	30.2	80.7	0.093

*Reaction conditions: 0.1 g cellulose, 170°C. Reproduced with permission from Cheng et al. (2011)*.

**Scheme 17:**
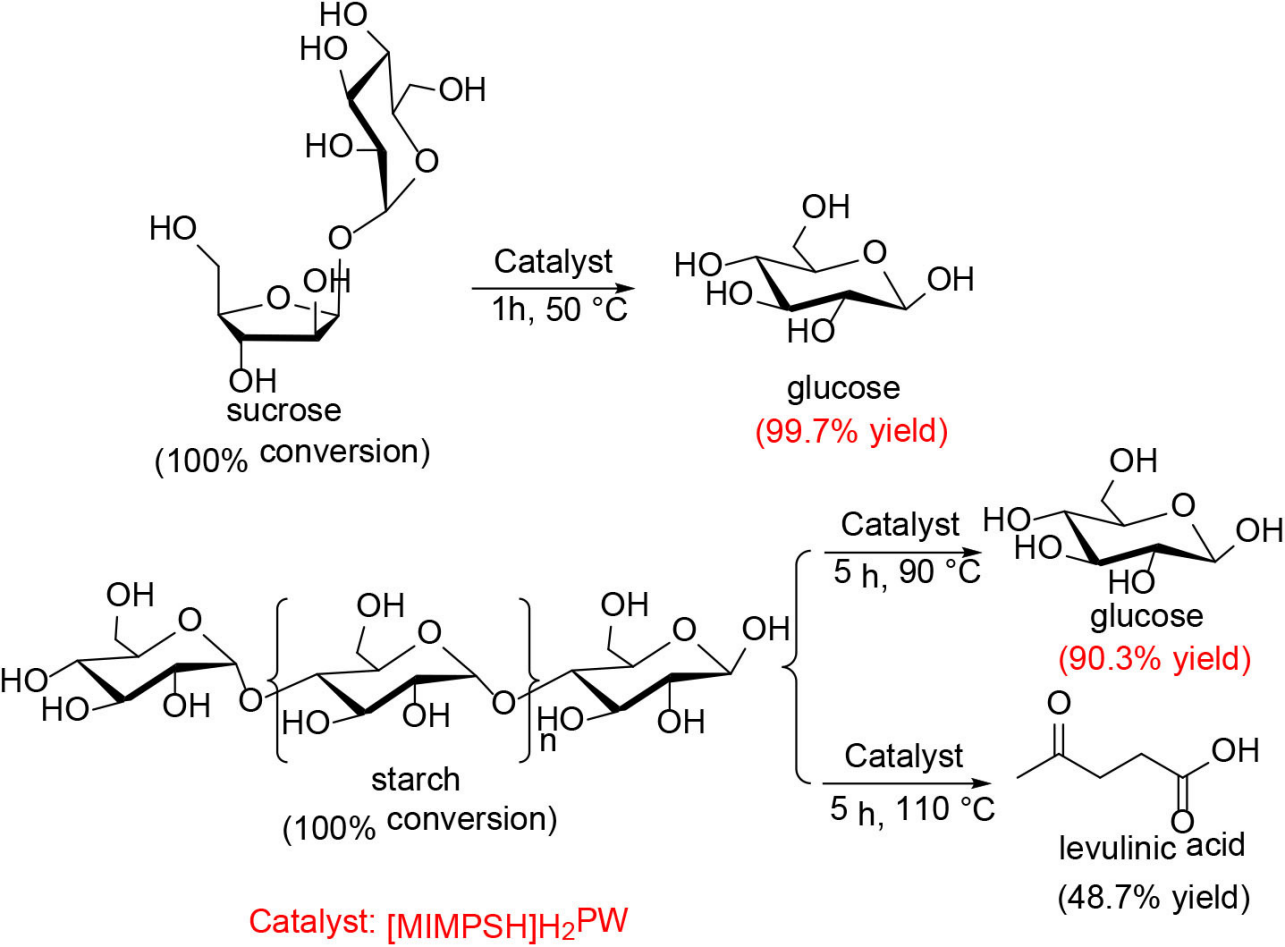
Conversion of other polysaccharides over the [MIMPSH]H_2_PW catalyst. Reaction conditions: 0.077 mmol catalyst, 0.5 mL water, and 5.0 mL MIBK.

**Figure 20:**
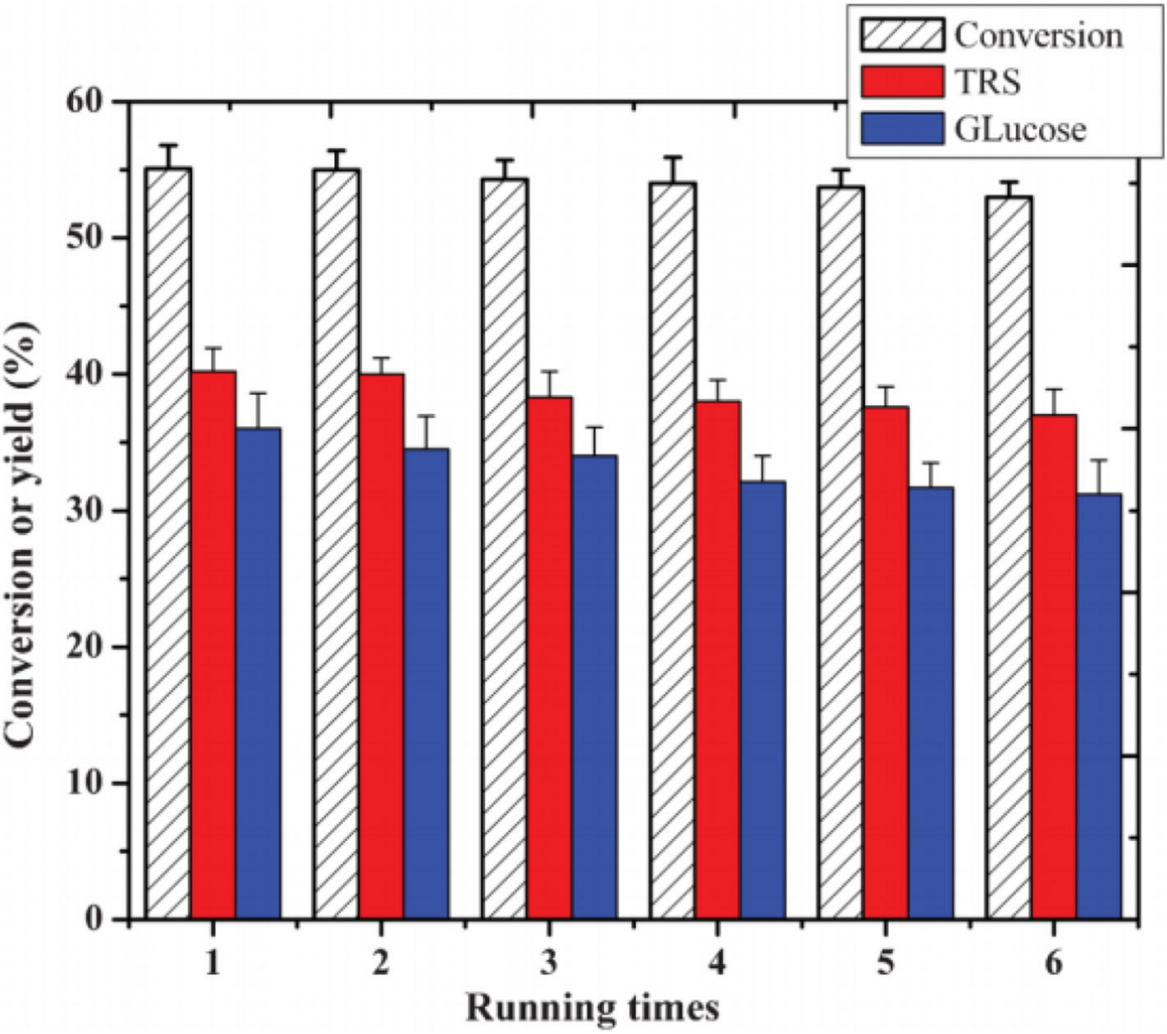
Reusability test of the [MIMPSH]H_2_PW catalyst. Reaction conditions: 0.1 g cellulose, 0.07 mmol catalyst, 0.5 mL H_2_O, 5 mL MIBK, 140°C for 5 h. Reproduced with permission from Sun et al. (2012).

In 2012, Sun et al. investigated a kind of HPA-ionic liquid catalysts [C_4_H_6_N_2_(CH_2_)_3_SO_3_H]_3−n_H_*n*_PW_12_O_40_ (*n* = 1, 2, 3) (abbreviated as [MIMPSH]_*n*_H_32n_PW), which can be used to degrade cellulose into water-soluble products such as glucose and levulinic acid (Sun et al., 2012). Glucose was the main product of hydrolysis of cellulose by the HPA-based on the ionic liquid catalyst, and the conversion of cellulose and the yield of glucose was 55.1 and 36.0%, respectively at 140°C for 5 h in a biphasic system (water–MIBK). Compared with the previously reported HPA catalysts, such as H_3_PW_12_O_40_ and Cs_2.5_H_0.5_PW_112_O_40_, the assembled catalyst showed much better catalytic performance because of its better solubility in water (Table 13). Moreover, the HPA-ionic liquids catalyst can also depolymerize other polysaccharides, from which high yields of glucose can be obtained (Scheme 17). More importantly, the HPA-ionic liquids catalyst can be completely recovered and reused six times without significant performance loss (Figure 20).

**Table 13:** Hydrolysis of cellulose with different catalysts^a^ (Sun et al., 2012).

**Catalyst**	**Conversion/%**	**TRS yield/%**	**Glucose yield (%)**	**TOF/g mmol^**−1**^**
MIMPSH	0	0	0	0
H_3_PW_12_O_40_	54.1	31.6	27.0	0.12
H_3_PW_12_O_40_ + IL^b^	57.3	33.8	27.1	0.12
[MIMPSH]H_2_PW	55.1 + 1.7	40.2 + 1.7	36.0 + 2.6	0.17
[MIMPSH]H_2_PW^c^	13.1 + 1.6	9.6 + 2.0	8.4 + 2.3	0.14
[MIMPSH]_2_HPW	42.7 + 1.3	35.9 + 1.9	27.3 + 1.8	0.13
[MIMPSH]_3_PW	28.4 + 1.4	25.2 + 1.5	23.2 + 2.5	0.11
Cs_2.5_H_0.5_PW_12_O_40_	12.3	10.0	8.1	

a Reaction conditions: 0.1 g cellulose, 0.07 mmol catalyst, 0.5 mL water and 5 mL MIBK (methyl isobutyl ketone) at 140°C for 5 h.

b 0.07 mmol H_3_PW_12_O_40_ and 0.07 mmol IL as the catalyst.

c *No MIBK, just 5.5 mL water. Reproduced with permission from Sun et al. (2012)*.

**Scheme 18:**
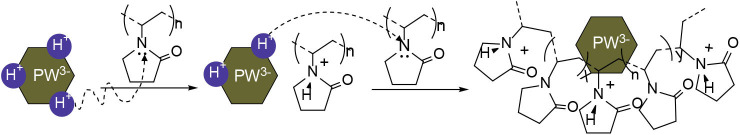
Synthetic routes to PVP-HPAs model.

**Scheme 19:**
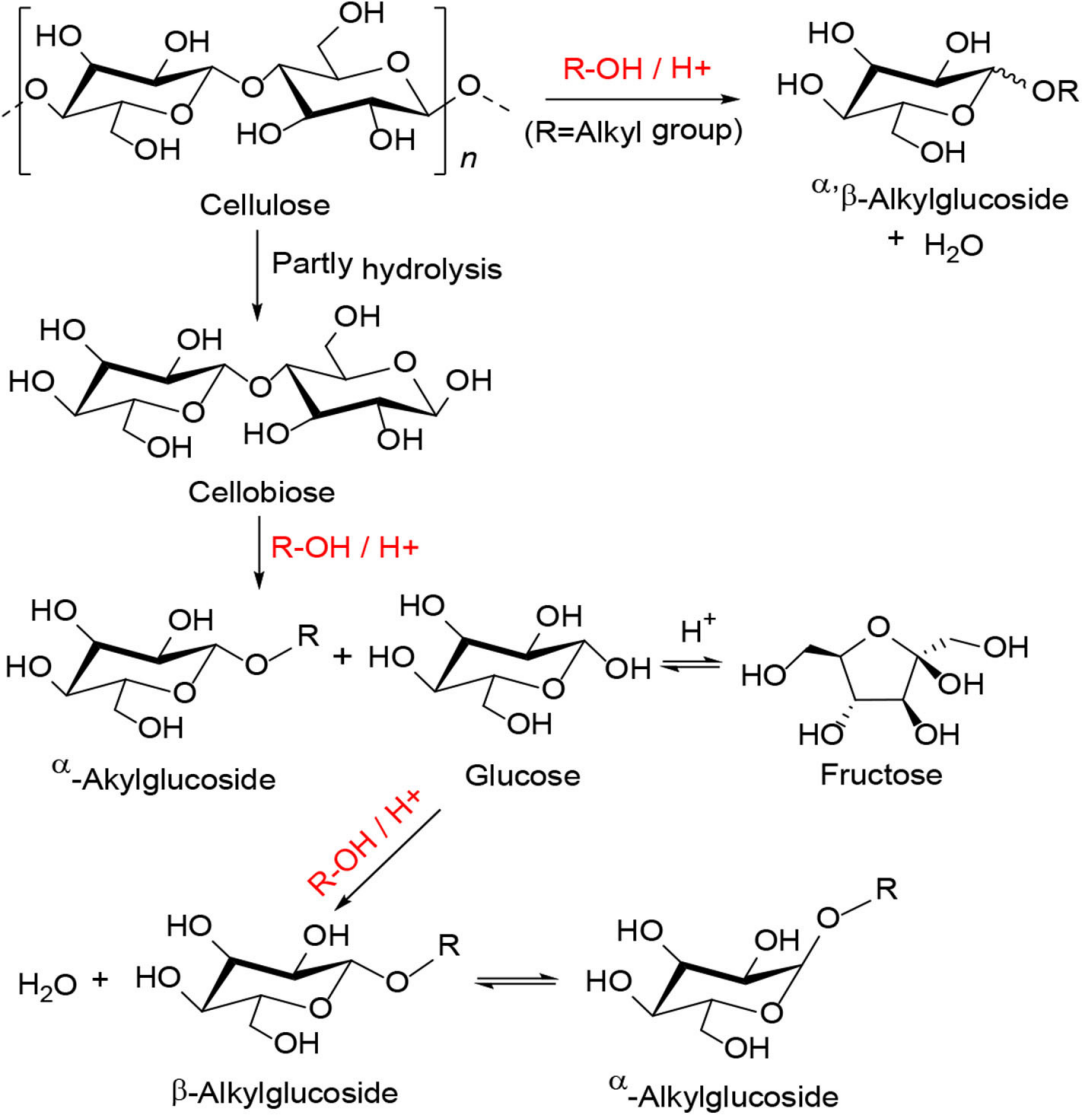
The conversion pathways and mechanism for the alcoholysis of cellulose.

**Figure 21:**
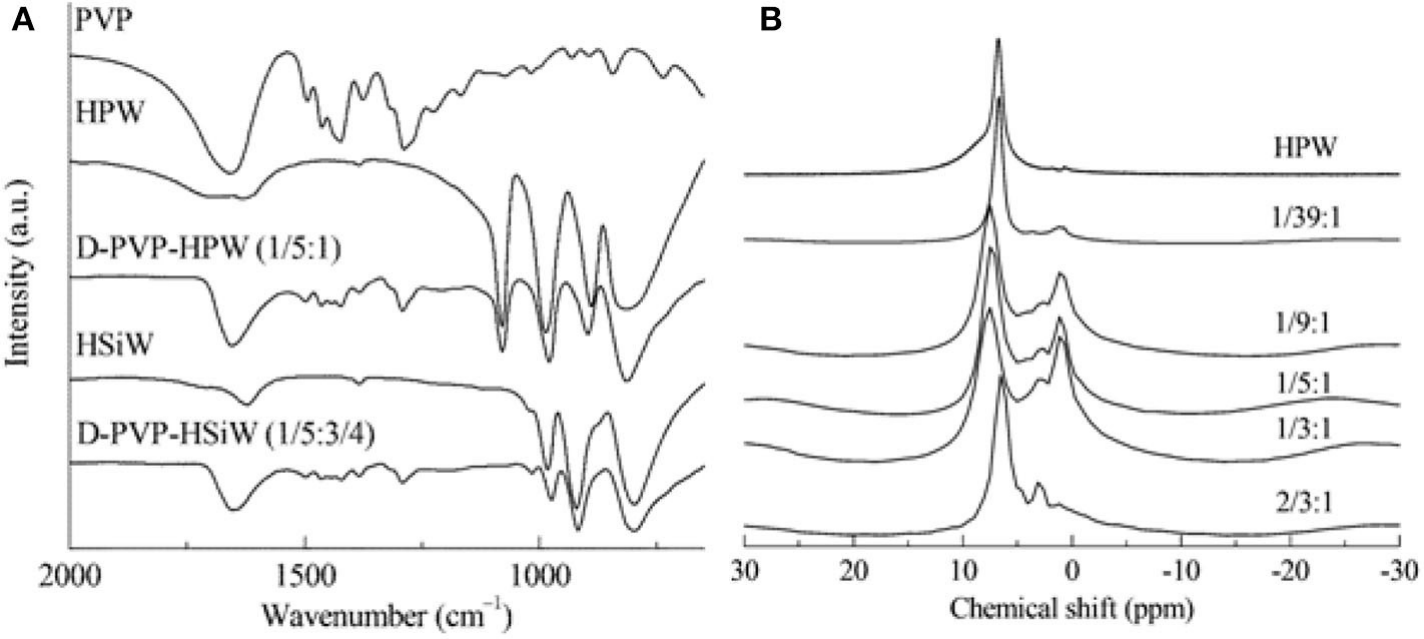
**(A)** The FT-IR spectra of PVP, HPAs, and PVP–HPAs, and **(B)** solid MAS ^1^H-NMR spectra of PVP–HPW catalysts. Reproduced with permission from Chen et al. (2013).

In 2013, Chen et al. synthesized the polyvinylpyrrolidone-stabilized heteropolyacids (PVP–HPAs), a kind of reusable self-assembling catalysts for conversion of cellulose (Chen et al., 2013). A model for the synthesis of PVP-HPAs is shown in Scheme 18 and the alcoholysis diagram of cellulose is shown in Scheme 19. First, cellobiose alcoholysis, then dehydration between glucose and alcohol molecules, and final interconversion between β-alkylglucoside and α-alkylglucoside gives the product. The authors used a variety of characteristic techniques (e.g., FT-IR and Solid MAS ^1^H-NMR) to study the structure of the self-assembling catalysts (Figures 21A,B), and they found that HPAs can protonate PVP and the protonated PVP can form the structure similar to the ionic liquid and interact with heteropolyanion (Chen et al., 2013). Moreover, the structure of PVP assembled with HPA contributes to the easy separation and reuse of catalysts. After optimizing the content of PVP and HPA, the results showed that the self-assembling catalyst PVP–H_4_SiW_12_O_4_0.5H_2_O (1/5:3/4) had excellent catalytic performance for the alcoholization of cellulose, and the conversion of cellulose was more than 60% (Chen et al., 2013). The application of the self-assembled catalyst is expected to expand to other reactions.

**Scheme 20:**
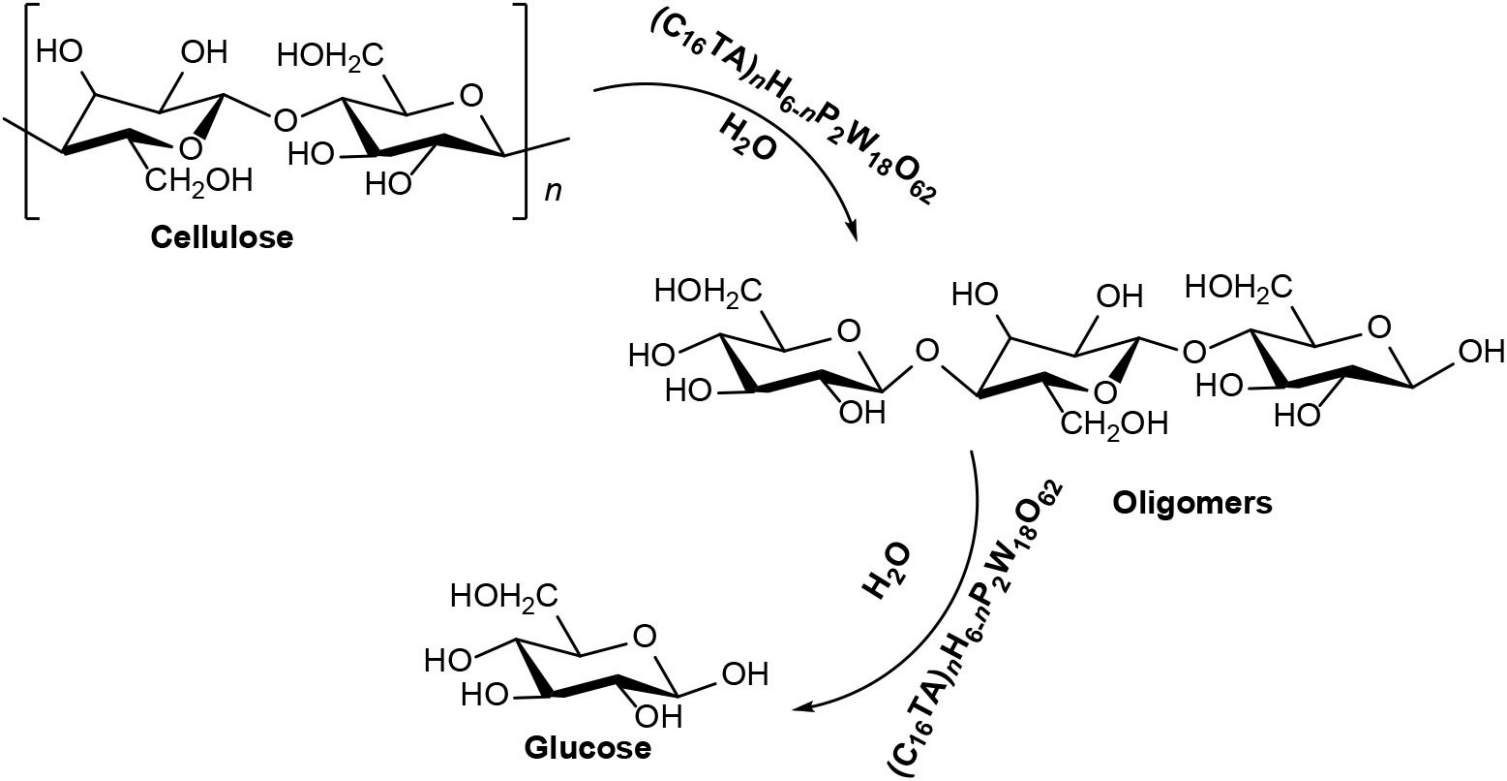
The mechanism of cellulose conversion by (C_16_TA)_*n*_H_6−n_P_2_W_18_O_62_ catalyst.

In 2015, Sun et al. synthesized an assembled HPA catalyst [C_16_H_33_N(CH_3_)_3_]_*x*_H_6−x_P_2_W_18_O_62_ (abbreviated as (C_16_TA)_*n*_H_6−n_P_2_W_18_O_62_). Wells–Dawson heteropolyacid H_6_P_2_W_18_O_62_ was used to synthesize the micellar assembly catalyst, which can catalyze the hydrolysis of cellulose to glucose (Sun et al., 2015). The mechanism of cellulose conversion by (C_16_TA)_*n*_H_6−n_P_2_W_18_O_62_ catalysts is shown in Scheme 20. The micelle catalyst first partially hydrolyzes cellulose to the oligomer, and then further degrades cellulose to glucose. Meanwhile, this catalyst can also promote the conversion of other polysaccharides, such as starch and cellobiose (Sun et al., 2015). The (C_16_TA)H_5_P_2_W_18_O_62_ (*n* = 1) catalyst exhibited higher catalytic activity than ordinary Keggin heteropoly acid catalyst H_3_PW_12_O_40_ (Table 14).

**Table 14:** Conversion of cellulose over different catalysts (Sun et al., 2015).

**Catalyst**	**Conversion/%**	**TRS yield/%**	**Glucose yield (%)**	**TOF/g mmol^**−1**^**
H_6_P_2_W_18_O_62_	93.5	52.6	45.6	12.9 × 10^−2^
(C_16_TA)H_5_P_2_W_18_O_62_	87.2	72.4	69.1	12.1 × 10^−2^
(C_16_TA)_2_H_4_P_2_W_18_O_62_	75.5	64.4	61.7	10.5 × 10^−2^
(C_16_TA)_3_H_3_P_2_W_18_O_62_	63.8	58.7	53.4	8.9 × 10^−2^
(C_16_TA)_4_H_2_P_2_W_18_O_62_	58.3	53.0	49.4	8.1 × 10^−2^
(C_16_TA)_5_HP_2_W_18_O_62_	43.5	40.3	37.8	6.0 × 10^−2^
(C_16_TA)_6_P_2_W_18_O_62_	30.6	28.5	25.7	4.3 × 10^−2^
(C_16_TA)H_2_PW_12_O_40_	81.6	75.3	63.3	11.3 × 10^−2^
H_3_PW_12_O_40_	55.7	46.3	44.2	7.7 × 10^−2^

*Reaction conditions: 100 mg cellulose, 0.08 mmol catalyst, 5 mL H_2_O, 160°C, 9 h. Reproduced with permission from Sun et al. (2015)*.

**Figure 22:**
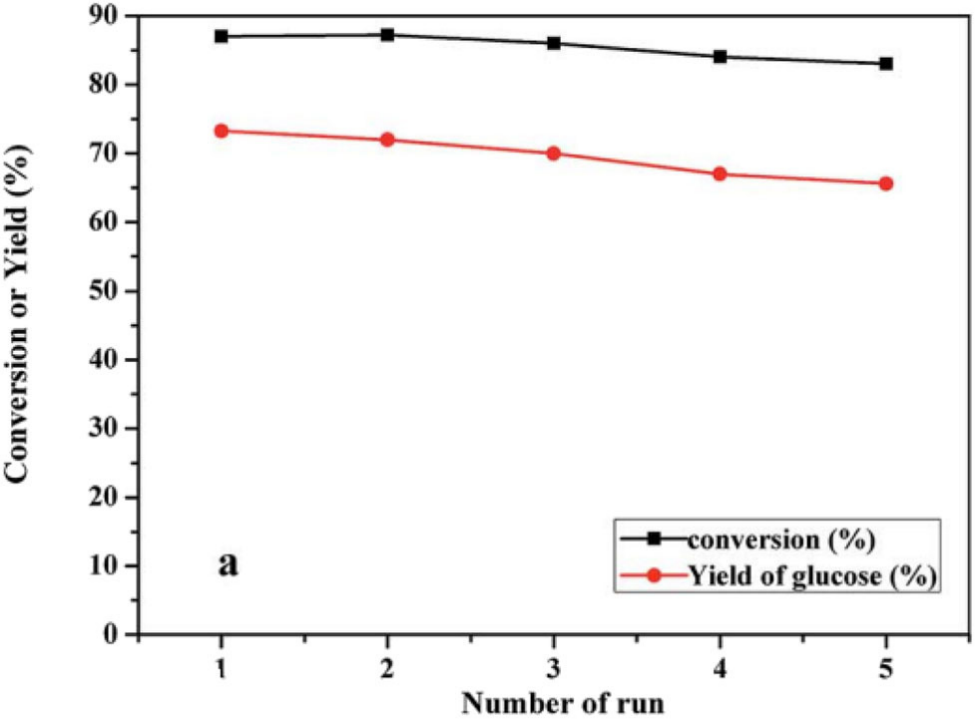
The circulation reaction test of the [C_16_H_33_N(CH_3_)_3_]H_5_P_2_W_18_O_62_ catalyst in conversion of cellulose to glucose. Reaction conditions: 100 mg cellulose, 0.08 mmol catalyst, 5 mL H_2_O, 160°C for 9 h. Reproduced with permission from Sun et al. (2015).

Sun et al. found that the high catalytic activity of the catalyst was due to the interaction of a high concentration of acid sites, which provided more opportunities for the substrate to enter the catalytic sites and the oxidation ability of (C_16_TA)_*n*_H_6−n_P_2_W_18_O_62_ (Sun et al., 2015). Moreover, this micellar assembled HPA catalyst was used as a heterogeneous catalyst and recycled by simple centrifuge (Figure 22).

**Figure 23:**
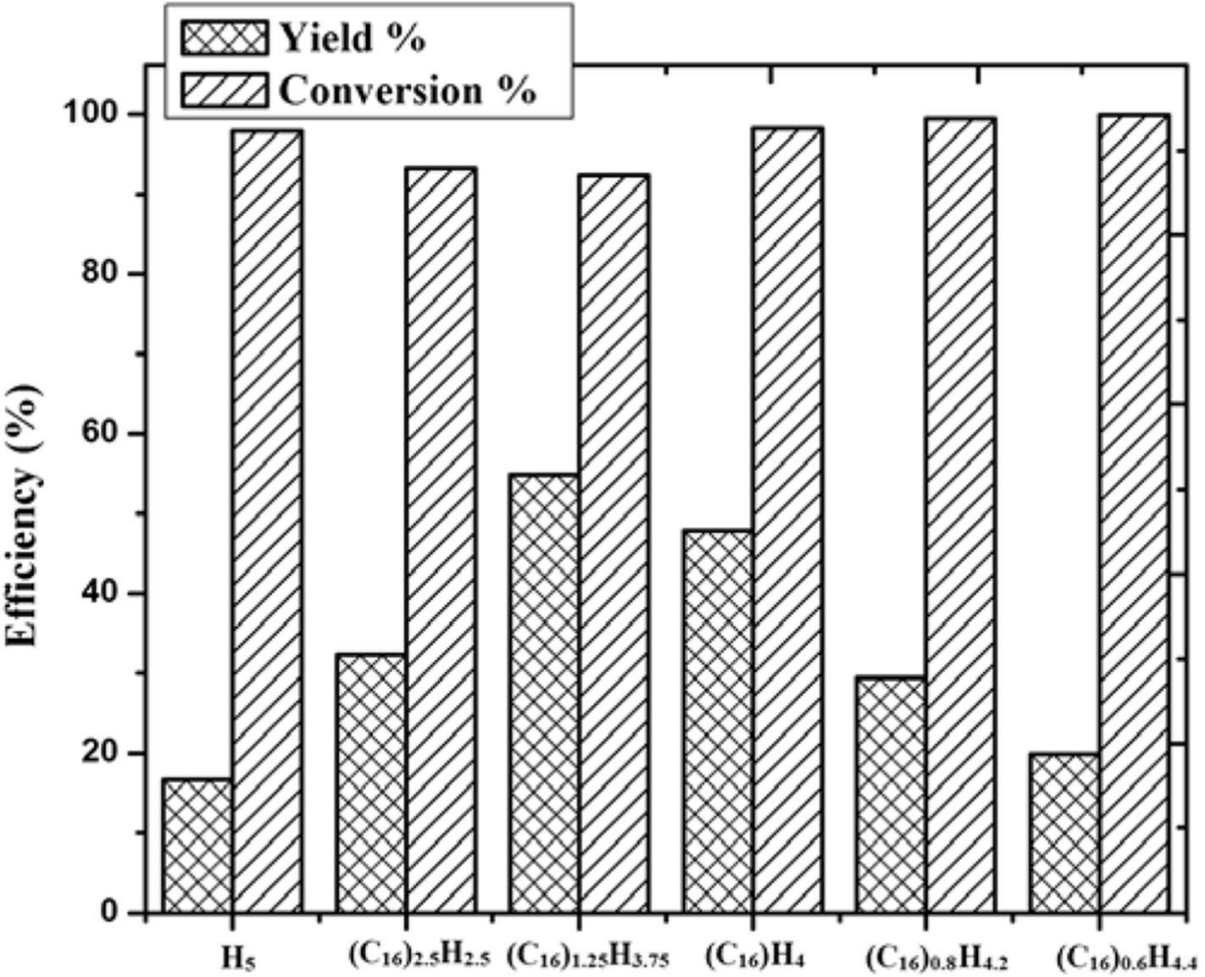
The efficiency of different catalysts for fructose transformation. Reaction conditions: 0.6 g fructose, 0.01 mmol catalyst, 2 mL H_2_O, 130°C for 1.5 h. Reproduced with permission from Lu et al. (2015).

**Figure 24:**
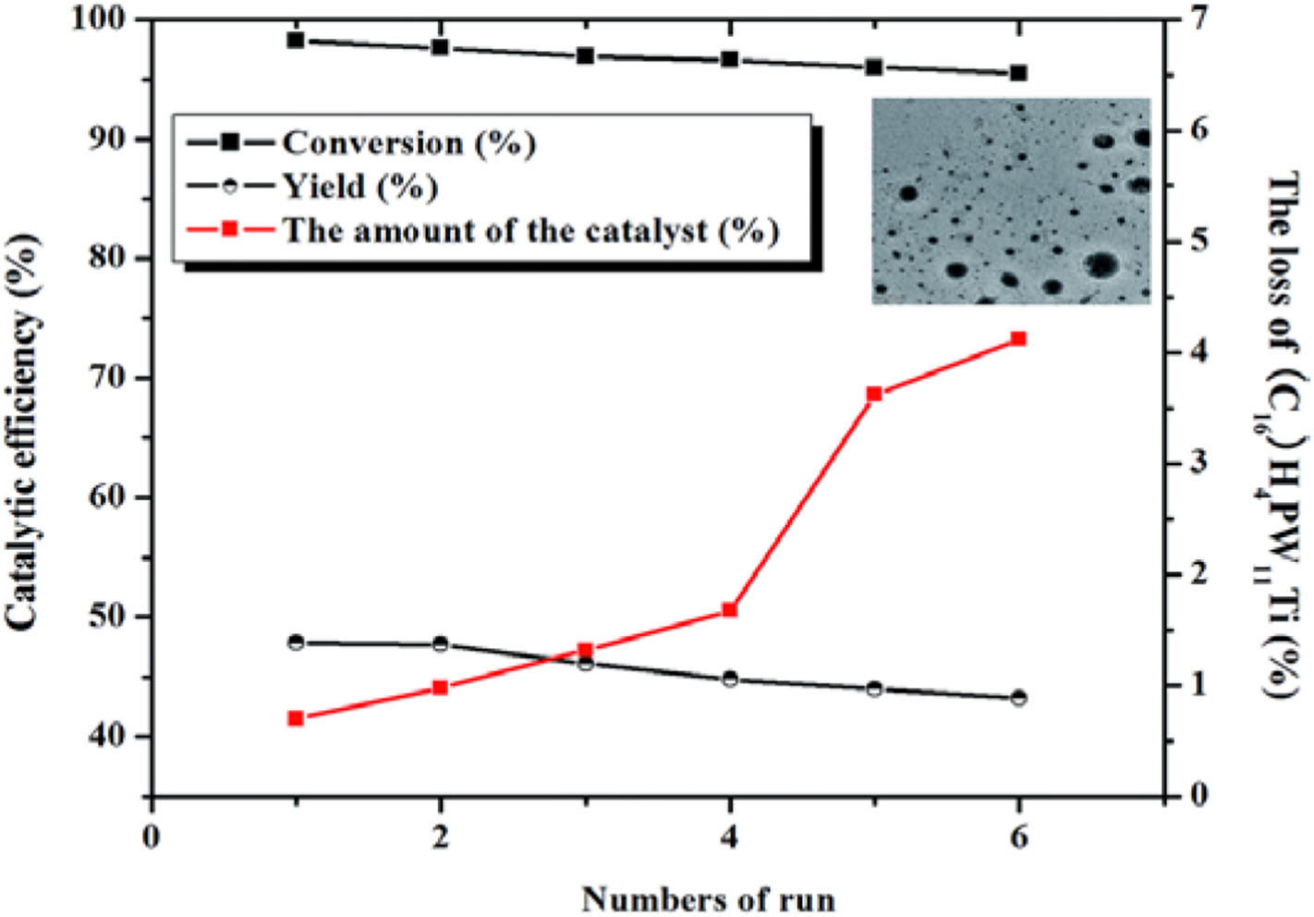
Recycling test of the (C_16_H_33_N(CH_3_)_3_)H_4_PW_11_TiO_40_ catalyst in the conversion of fructose to HMF. Reaction conditions: 0.6 g fructose, 0.01 mmol catalyst, 2 mL H_2_O, 130°C for 1.5 h. Reproduced with permission from Lu et al. (2015).

Lu et al. prepared heterogeneous catalysts (C_*n*_H_2n+1_N(CH_3_)_3_)H_4_PW_11_TiO_40_ (*n* = 4, 8, 12, 14, 16, and 18) composed of HPAs and amphiphilic quaternary ammonium salt by self-assembly method (Lu et al., 2015). The results showed that the heterogeneous catalyst (C_16_H_33_N(CH_3_)_3_)H_4_PW_11_TiO_40_ had the strongest acidity and exhibited excellent activity in the conversion of saccharides to other useful substances, with HMF yield of 53.7 and 50.8% from fructose and glucose, respectively (Figure 23). Meanwhile, the self-assembled structure of (C_16_H_33_N(CH_3_)_3_)H_4_PW_11_TiO_40_ in water could make cellulose and other polysaccharides gather to the catalytic sites, thus improving the catalytic efficiency. Also, the catalyst can be easily separated from the reactant, and the activity was almost no loss after recycling for many times (Figure 24).

It can be seen that the assembled HPA catalysts can convert cellulose into glucose or other substances. Their unique structure can make cellulose aggregate to the catalytic sites, thus improving the catalytic efficiency. And almost all of them can be recycled by simple instruments. These advantages make their research more meaningful. However, it can not be ignored that some supported catalysts have low selectivity and yield for glucose, such as (C_16_TA)H_5_P_2_W_18_O_62_ and [MIMPSH]H_2_PW. Moreover, the stability of some HPA catalysts decreased after reuse, which should be improved by the exploration of novel preparation methods.

### Other HPA Catalysts

In addition to the HPA catalysts mentioned above, many other HPAcatalysts have also been developed and utilized for cellulose degradation. For example, HPAs can be combined with other catalysts to facilitate cellulose conversion. Palkovits et al. proved that the combination of HPAs and supported Ru catalysts can directly convert cellulose into glycols with a yield of 81% (Palkovits et al., 2011). And the catalyst is very easy to recycle. In 2014, Xie et al. used HPA to synthesize a new catalyst Ru/[Bmim]_3_PW_12_O_40_, which can selectively convert microcrystalline cellulose to hexanol under mild conditions (Xie et al., 2014). The catalyst was synthesized by supporting Ru on ionic liquid (BmimPF_6_)-heteropoly acid (H_3_PW_12_O_40_·nH_2_O), which resulted in the formation of Brønsted acid sites. It improves the performance of the catalyst and is an important reason for the remarkable activity of the catalyst in the conversion of cellulose. Su et al. used HPA and ZrO_2_ to synthesize a kind of dual-functional heterogeneous catalyst (H_3_PW_12_O_40_/ZrO_2_-Et-HNS), which was composed of organosilicon hollow nanospheres and can be used in esterification (Su et al., 2014). The unique hollow nanospherical morphology of the catalyst increases its surface acidity and thus improves its catalytic activity. Basahel et al. (2016) synthesized a heterogeneous catalyst Al_*x*_H_3x_PW_12_O_40_ by ion-exchange method. The HPA catalysts formed by the exchange of hydrogen and aluminum ions showed higher activity because of the increase of Lewis acid sites on the surface of the catalysts. These catalysts proved to be able to synthesize bioactive pyrido[1,2-a]pyrimidines with yields above 90%. In 2017, Wu et al. synthesized a new luryamine-modified

phosphotungstic acid catalyst. They used several characterization instruments to characterize the catalyst and found that the modified heteropoly acid was still the Keggin structure (Wu et al., 2017). As compared with conventional phosphotungstic acid, the luryamine-modified HPA catalyst showed more stable and higher catalytic activity. It's worth mentioning that the yield of glucose reached 100% by hydrolysis of sucrose with the modified catalyst which can provide a way to study cellulose hydrolysis in the future. All the HPA catalysts reported above are heterogeneous. They have a common feature that they are easy to separate from reactants and can be reused with almost no loss of catalytic activity.

### Comparison of Different HPA-Based Catalysts

As discussed above, it can be concluded that the Keggin HPA-based catalysts are one of the most common cellulose degradation catalysts. Due to their dual functional properties, the HPA-based catalysts can further promote the oxidation of hydrolysates into valuable compounds. However, their specific surface areas are relatively low, which is not conducive to the catalytic activity. To solve this deficiency, some substituted and supported heteropoly acid catalysts were developed, which are demonstrated to have higher specific surface areas, higher reactivity, and better thermal stability, and are more conducive to the separation because they are commonly used in the heterogeneous systems. It is worth noting that the preparation of these catalysts is complicated and the synthesis cycle is long. In order to further improve the catalytic activity of HPAs, the assembled HPAs and other modified HPA catalysts have been developed. With unique structures, these catalysts exhibit high catalytic performance in cellulose conversion. More importantly, they are all heterogeneous catalysts, providing convenience for the catalyst separation and recycling processes. Therefore, different HPA catalysts have their own characteristics, advantages and disadvantages, and the catalytic upgrading of cellulosic biomass can be effectively enabled with these HPA-based catalysts as long as their properties are properly switched.

## Conclusions and Outlook

With the rapid development and utilization of chemical fuels, depolymerization of cellulosic biomass has become one of the ways to provide organic carbon sources. It is reported that cellulose can be converted into a variety of useful platform molecules that can be applied in various fields. In this review, the focus is placed on finding homogeneous and heterogeneous HPA catalysts for cellulose degradation.

HPAs, as a king of efficient, environmentally friendly, and safe catalysts, show high catalytic activity for cellulose degradation. HPA catalysts have higher catalytic activity than conventional solid acid catalysts, which are more conducive to the degradation of cellulose into valuable platform molecules, such as glucose. Meanwhile, due to its unique physical and chemical properties, HPAs can be used in homogeneous and heterogeneous systems. And they can all be reused after separation in the reactor with almost no loss of catalytic activity.

Several methods have been developed to synthesize HPA catalysts. (1) The acidification process coupled with an ether extraction is the most commonly used method, which is mainly used for the synthesis of common HPAs (e.g., H_3_PW_12_O_40_, H_3_SiW_12_O_40_), but its safety is low due to the use of some toxic substances. (2) The ion-exchange method has high safety, but it has the disadvantage of a long production cycle. (3) The impregnation method is simple and safe but suffers the disadvantage of the loss of catalyst activity and limited recycling ability. (4) The sol-gel method can prepare HPA catalysts at low temperatures or mild conditions, which is widely used in the synthesis of HPA-based nanomaterial catalysts. However, this method has the disadvantages of low security and long cycle. Therefore, it is very important and urgent to develop more efficient methods for the preparation of HPA-based catalysts.

As depicted in previous studies, typical Keggin-type HPA catalysts are the most common ones for cellulose degradation, and they can further catalyze the oxidation of the hydrolysates to valuable substances due to their dual functional properties. Then, the supported and substituted HPA catalysts always exist in heterogeneous form. After supporting and substituting disposal, the acid site number, the acid strength, and the specific surface area of the HPA catalysts increase, which improves the catalytic properties of the catalysts. In addition, the assembled HPA catalysts and other modified HPA catalysts show good catalytic performance in cellulose conversion because of their unique structure. Therefore, HPA-based catalysts provide convenience for cellulose degradation and more possibilities for the development of chemical fuels in the future. But it cannot be neglected that some of HPAs have the disadvantages of high catalyst dosage and relatively low catalytic activity with respect to the reaction conversion, product selectivity, and catalyst stability. For example, the selectivity of substituted and supported HPAs toward target products is lower than that of Keggin-type HPAs. Also, the stability of the supported HPAs is lower than that of common HPAs.

Although HPAs-based catalysts in either homogeneous or heterogeneous state have made great progress in the degradation of biomass cellulose, there are still many deficiencies to be improved:

(1) Study on the relationship between the properties of HPA catalysts and the degradation of cellulose to further improve the catalytic activity.

(2) Design of a cleaner, safer, and more effective HPA catalyst with simple preparation methods to promote cellulose degradation.

(3) Development of more new HPA catalysts with special structure, and ensure no or fewer side reactions in the reaction process, with the focus on the improvement of the HPA catalyst disadvantages especially unsatisfactory conversion, selectivity, and stability.

(4) Further understanding the structural characteristics of HPAs, study of the reaction mechanism catalyzed by HPAs, and promotional completion of the target reaction by linking the relationship between them.

(5) Extension of HPA catalysts to other biomass feedstocks, providing more help for the future development of fuels and HPAs in the field of catalysis.

In summary, this review mainly summarizes some examples of the degradation of cellulose by some kinds of HPA catalysts. The HPA catalysts are considered to have great application prospects in the degradation of cellulose and the conversion of other relevant research fields.

## Author Contributions

XL and HL initiated the project. XL, HW, CL, ZL, HL, HZ, YL, YS, and SY searched the data and wrote, revised, and completed the manuscript. All authors contributed to the article and approved the submitted version.

## Conflict of Interest

The authors declare that the research was conducted in the absence of any commercial or financial relationships that could be construed as a potential conflict of interest.

## References

[B1] Ahmed A. I. El-Hakam S. A. Elghany M. A. A. El-Yazeed W. S. A. (2011). Synthesis and characterization of new solid acid catalysts, H_3_PW_12_O_40_ supported on nanoparticle tin oxide: An efficient catalyst for the preparation of 7-hydroxy-4-methylcoumarin. Appl. Catal. A Gen. 407, 40–48. 10.1016/j.apcata.2011.08.020

[B2] Akiyama G. Matsuda R. Sato H. Takata M. Kitagawaet S. (2011). Cellulose hydrolysis by a new porous coordination polymer decorated with sulfonic acid functional groups. Adv. Mater. 23, 3294–3297. 10.1002/adma.201101356 21661069

[B3] Albert J. Luders D. Bosmann A. Guldi D. M. Wasserscheid P. (2014). Spectroscopic and electrochemical characterization of heteropoly acids for their optimized application in selective biomass oxidation to formic acid. Green Chem. 16, 226–237. 10.1039/C3GC41320A

[B4] Albert J. Wolfel R. Bosmann A. Wasserscheid P. (2012). Selective oxidation of complex, water-insoluble biomass to formic acid using additives as reaction accelerators. Energy Environ. Sci. 5, 7956–7962. 10.1039/c2ee21428h

[B5] An D. Ye A. Deng W. Zhang Q. Wang Y. (2012). Selective conversion of cellobiose and cellulose into gluconic acid in water in the presence of oxygen, catalyzed by polyoxometalate-supported gold nanoparticles. Chem. Eur. J. 18, 2938–2947. 10.1002/chem.201103262 22298297

[B6] Bachillerbaeza B. Anderson J. (2004). FTIR and reaction studies of the acylation of anisole with acetic anhydride over supported hpa catalysts. J. Catal. 228, 225–233. 10.1016/j.jcat.2004.08.010

[B7] Bamoharram F. F. Heravi M. M. Heravi H. M. Dehghan M. (2004). Photocatalytic oxidation of benzyl alcohols in the presence of H_14_[NaP_5_W_30_O_110_] as a green and reusable catalyst. Synth. React. Inorg. Metal Org. Nano Metal Chem. 39, 394–399. 10.1080/15533170903129745

[B8] Basahel S. N. Ahmed N. S. Narasimharao K. Mokhtar M. (2016). Simple and efficient protocol for synthesis of pyrido [1, 2-a] pyrimidin-4-one derivatives over solid heteropolyacid catalysts. RSC Adv. 6, 11921–11932. 10.1039/C5RA22180C

[B9] Bechtold M. F. Square K. (1950). Method of Preparing Heteropolyacids Containing a Heavy Metal. U.S. Patent No. 2503991.

[B10] Bennardi D. Romanelli G. Autino J. Pizzio L. Vázquez P. Cáceres C. (2010). Comparative study of the catalytic preparation of flavones using keggin heteropolyacids under homogeneous, heterogeneous and solvent free conditions. React. Kinet. Mech. Catal. 100, 165–174. 10.1007/s11144-010-0172-4

[B11] Cardoso L. A. M. Alves W. Gonzaga A. R. E. Aguiar L. G. Andrade H. M. C. (2004). Friedel-crafts acylation of anisole with acetic anhydride over silica-supported heteropolyphosphotungstic acid (HPW/SiO_2_). J. Mol. Catal. A Chem. 209, 189–197. 10.1016/j.molcata.2003.08.022

[B12] Chang F. Hanna M. A. Zhang D. Li H. Zhou Q. Song B.. (2013). Production of biodiesel from non-edible herbaceous vegetable oil: *Xanthium sibiricum* Patr. Bioresour. Technol. 140, 435–438. 10.1016/j.biortech.2013.04.111 23714693

[B13] Chen J. Fang X. Duan X. Ye L. Lin H. Yuan Y. (2013). PVP-stabilized heteropolyacids as reusable self-assembling catalysts for alcoholysis of cellulosic saccharides. Green Chem. 16, 294–302. 10.1039/C3GC40994E

[B14] Cheng M. Tian S. Guan H. Wang S. Wang X. Jiang Z. J. (2011). Clean production of glucose from polysaccharides using a micellar heteropolyacid as a heterogeneous catalyst. Appl. Catal. B Environ. 107, 104–109. 10.1016/j.apcatb.2011.07.002

[B15] Chheda J. N. Huber G. W. Dumesic J. A. (2007). Liquid-phase catalytic processing of biomass-derived oxygenated hydrocarbons to fuels and chemicals. Angew. Chem. Int. Ed. 46, 7164–7183. 10.1002/anie.200604274 17659519

[B16] Chiola V. Lawrence J. G. (1969). Preparation of Heteropoly Molybdic Acids. U.S. Patent No. 3425794. Washington, DC: U.S. Patent and Trademark Office.

[B17] Chiola V. vanderpool C. D. Towanda P. (1969). Method of Preparing Heteropoly Compounds. U.S. Patent No. 3446575. Washington, DC: U.S. Patent and Trademark Office.

[B18] Climent M. J. Corma A. Iborra S. (2014). Conversion of biomass platform molecules into fuel additives and liquid hydrocarbon fuels. Green Chem. 16, 516–547. 10.1039/c3gc41492b

[B19] de Op Beeck B. Geboers J. van de Vyver S. (2013). Conversion of (ligno)cellulose feeds to isosorbide with heteropoly acids and Ru on carbon. ChemSusChem 6, 199–208. 10.1002/cssc.201200610 23307750

[B20] Deng W. Zhang Q. Wang Y. (2012). Polyoxometalates as efficient catalysts for transformations of cellulose into platform chemicals. Dalton Trans. 41, 9817–9831. 10.1039/c2dt30637a 22653050

[B21] Dhepe P. L. Fukuoka A. (2008). Cellulose conversion under heterogeneous catalysis. ChemSusChem 1, 969–975. 10.1002/cssc.200800129 19021143

[B22] Duan X. Sun G. Sun Z. Li J. Wang S. Wang X. (2013). A heteropolyacid-based ionic liquid as a thermoregulated and environmentally friendly catalyst in esterification reaction under microwave assistance. Catal. Commun. 42, 125–128. 10.1016/j.catcom.2013.08.014

[B23] Dwars T. Paetzold E. Oehme G. (2005). Reactions in micellar systems. Angew. Chem. Int. Ed. 44, 7174–7199. 10.1002/anie.200501365 16276555

[B24] Feng J. Zhu Q. Ding M. Liu X. Han X. (2011). Direct conversion and nmr observation of cellulose to glucose and 5-hydroxymethylfurfural (HMF) catalyzed by the acidic ionic liquids. J Mol. Catal. A Chem. 334, 8–12. 10.1016/j.molcata.2010.10.006

[B25] Furukawa H. Kobayashi N. Itaya Y. Satsuma A. Shimizu K. (2009). Effects of brøNsted and lewis acidities on activity and selectivity of heteropolyacid-based catalysts for hydrolysis of cellobiose and cellulose. Green Chem. 11, 1627–1632. 10.1039/b913737h

[B26] Geboers J. van de Vyver S. Carpentier K. de Blochouse K. Jacobs P. Sels B. (2010). Efficient catalytic conversion of concentrated cellulose feeds to hexitols with heteropoly acids and Ru on carbon. Chem. Commun. 46, 3577–3580. 10.1039/c001096k 20376382

[B27] Geboers J. van de Vyver S. Carpentier K. Jacobs P. Sels B. (2011). Hydrolytic hydrogenation of cellulose with hydrotreated caesium salts of heteropoly acids and Ru/C. Green Chem. 13, 2167–2174. 10.1039/c1gc15350a

[B28] Gromov N. V. Taran O. P. Delidovich I. V. Pestunov A. V. Rodikova Y. A. Yatsenko D. A. (2016). Hydrolytic oxidation of cellulose to formic acid in the presence of Mo-V-P heteropoly acid catalysts. Catal. Today 278, 74–81. 10.1016/j.cattod.2016.03.030

[B29] Guo Y. Li K. Clark J. H. (2007). The synthesis of diphenolic acid using the periodic mesoporous H_3_PW_12_O_40_-silica composite catalysed reaction of levulinic acid. Green Chem. 9, 839–841 10.1039/b702739g

[B30] Hafizi A. Ahmadpour A. Heravi M. M. Bamoharram F. F. (2014). The application of silica-supported preyssler hpa as a heterogeneous and green catalyst for the alkylation of benzene. Petrol. Sci. Technol. 32, 1022–1027. 10.1080/10916466.2011.637534

[B31] Harada T. Tokai Y. Kimura A. Ikeda S. Matsumura M. (2014). Hydrolysis of crystalline cellulose to glucose in an autoclave containing both gaseous and liquid water. RSC Adv. 4, 26838–26842. 10.1039/c4ra02396j

[B32] He J. Li H. Riisager A. Yang S. (2018). Catalytic transfer hydrogenation of furfural to furfuryl alcohol with recyclable Al–Zr@Fe mixed oxides. ChemCatChem 10, 430–438. 10.1002/cctc.201701266

[B33] Heravi M. M. Jani B. A. Derikvand F. Bamoharram F. F. Oskooie H. A. (2008). Three component, one-pot synthesis of dihydropyrano[3,2-c]chromene derivatives in the presence of H_6_P_2_W_18_O_62_·18H_2_O as a green and recyclable catalyst. Catal. Commun. 10, 272–275. 10.1016/j.catcom.2008.08.02318780153

[B34] Heravi M. M. Khorasani M. Derikvand F. Oskooie H. A. Bamoharram F. F. (2007). Highly efficient synthesis of coumarin derivatives in the presence of H_14_[NaP_5_W_30_O_110_] as a green and reusable catalyst. Catal. Commun. 8, 1886–1890. 10.1016/j.catcom.2007.02.030

[B35] Hu S. Jiang F. Hsieh Y. L. (2015). 1D lignin-based solid acid catalysts for cellulose hydrolysis to glucose and nanocellulose. ACS Sustain. Chem. Eng. 3, 2566–2574. 10.1021/acssuschemeng.5b00780

[B36] Huber G. W. Iborra S. Corma A. (2006). Synthesis of transportation fuels from biomass: chemistry, catalysts, and engineering. Chem. Rev. 106, 4044–4098. 10.1021/cr068360d 16967928

[B37] Izumi Y. Matsuo K. Urabe K. (1983). Efficient homogeneous acid catalysis of heteropoly acid and its characterization through ether cleavage reactions. J. Mol. Catal. 18, 299–314. 10.1016/S0304-5102(83)80004-2

[B38] Izumi Y. Ono M. Kitagawa M. Yoshida M. Urabe K. (1995). Silica-included heteropoly compounds as solid acid catalysts. Microporous Mater. 5, 255–262. 10.1016/0927-6513(95)00059-3

[B39] Kamm B. Kamm M. (2004). Principles of biorefineries. Appl. Microbiol. Biotechnol. 64, 137–145. 10.1007/s00253-003-1537-7 10.1007/s00253-003-1537-7 14749903

[B40] Kaur J. Kozhevnikov I. V. (2002). Efficient acylation of toluene and anisole with aliphatic carboxylic acids catalysed by heteropoly salt Cs_2.5_H_0.5_PW_12_O_40_. Chem. Commun. 8, 2508–2509. 10.1039/b207915c

[B41] Klein M. Pulidindi I. N. Perkas N. Gedanken A. (2015). Heteropoly acid catalyzed hydrolysis of glycogen to glucose. Biomass Bioenergy 76, 61–68. 10.1016/j.biombioe.2015.02.036

[B42] Klein M. Pulidindi I. N. Perkas N. Meltzer-Mats E. Gruzman A. Gedanken A. (2012). Direct production of glucose from glycogen under microwave irradiation. RSC Adv. 2, 7262–7267. 10.1039/c2ra21066e

[B43] Klemm D. Heublein B. Fink H. P. Bohn A. (2005). Cellulose: fascinating biopolymer and sustainable raw material. Angew. Chem. Int. Ed. 44, 3358–3393. 10.1002/anie.200460587 15861454

[B44] Kobayashi H. Komanoya T. Hara K. Fukuoka A. (2010). Water-tolerant mesoporous-carbon-supported ruthenium catalysts for the hydrolysis of cellulose to glucose. ChemSusChem 3, 440–443. 10.1002/cssc.200900296 20198680

[B45] Komanoya T. Kobayashi H. Hara K. Chun W. J. Fukuoka A. (2011). Catalysis and characterization of carbon-supported ruthenium for cellulose hydrolysis. Appl. Catal. A Gen. 407, 188–194. 10.1016/j.apcata.2011.08.039

[B46] Kozhevnikov I. V. (1987). Advances in catalysis by heteropolyacids. Russ. Chem. Rev. 56, 811–825. 10.1070/RC1987v056n09ABEH003304

[B47] Kozhevnikov I. V. (1998). Catalysis by heteropoly acids and multicomponent polyoxometalates in liquid-phase reactions. Chem. Rev. 98, 171–198. 10.1021/cr960400y 11851502

[B48] Laferty J. M. John M. (1966). Method of Preparing Phosphotungstic Acid. U.S. Patent No. 3288562. Washington, DC: U.S. Patent and Trademark Office.

[B49] Lai D. M. Deng L. Guo Q. X. Fu Y. (2011). Hydrolysis of biomass by magnetic solid acid. Energy. Environ. Sci. 4, 3552–3557. 10.1039/c1ee01526e

[B50] Lanzafame P. Temi D. M. Perathoner S. Spadaro A. N. Centi G. (2012). Direct conversion of cellulose to glucose and valuable intermediates in mild reaction conditions over solid acid catalysts. Catal. Today 179, 178–184. 10.1016/j.cattod.2011.07.018

[B51] Leng Y. Wang J. Zhu D. Ren X. Ge H. Shen L. (2010). Heteropolyanion-based ionic liquids: reaction-induced self-separation catalysts for esterification. Angew. Chem. 48, 168–171. 10.1002/anie.200803567 19053112

[B52] Li H. Fang Z. Yang S. (2016). Direct catalytic transformation of biomass derivatives into biofuel component γ-valerolactone with magnetic nickel-zirconium nanoparticles. ChemPlusChem 81, 135–142. 10.1002/cplu.201500492 31968733

[B53] Li H. He X. Zhang Q. Chang F. Xue W. Zhang Y. (2013). Polymeric ionic hybrid as solid acid catalyst for the selective conversion of fructose and glucose to 5-hydroxymethylfurfural. Energy Technol. 1, 151–156. 10.1002/ente.201200041

[B54] Li H. Liu X. Yang T. Zhao W. Saravanamurugan S. Yang S. (2015). Porous zirconium–furandicarboxylate microspheres for efficient redox conversion of biofuranics. ChemSusChem 10, 1761–1770. 10.1002/cssc.201601898 28164471

[B55] Li H. Zhang Q. Bhadury P. S. Yang S. (2014). Furan-type compounds from carbohydrates via heterogeneous catalysis. Curr. Org. Chem. 18, 547–597. 10.2174/13852728113176660138

[B56] Liu B. Zhang Z. H. (2015). Catalytic conversion of biomass into chemicals and fuels over magnetic catalysts. ACS Catal. 6, 326–338. 10.1021/acscatal.5b02094

[B57] Liu Q. Y. Wu W. L. Wang J. Ren X. Q. Wang Y. R. (2004). Characterization of 12-tungstophosphoric acid impregnated on mesoporous silica SBA-15 and its catalytic performance in isopropylation of naphthalene with isopropanol. Micropor. Mesopor. Mater. 76, 51–60. 10.1016/j.micromeso.2004.08.001

[B58] Liu X. Li H. Pan H. Zhang H. Huang S. Yang K. (2016). Efficient catalytic conversion of carbohydrates into 5-ethoxymethylfurfural over MIL-101-based sulfated porous coordination polymers. J. Energy Chem. 25, 523–530. 10.1016/j.jechem.2016.01.015

[B59] Lu T. Niu M. Hou Y. Wu W. Ren S. Yang F. (2016). Catalytic oxidation of cellulose to formic acid in H_5_PV_2_Mo_10_O_40_ + H_2_SO_4_ aqueous solution with molecular oxygen. Green Chem. 18, 4725–4732. 10.1039/C6GC01271J

[B60] Lu Y. Sun Z. Huo M. (2015). Fabrication of a micellar heteropolyacid with lewis–brønsted acid sites and application for the production of 5-hydroxymethylfurfural from saccharides in water. RSC Adv. 5, 30869–30876. 10.1039/C4RA16952B

[B61] Macht J. Janik M. J. Neurock M. Iglesia E. (2008). Mechanistic consequences of composition in acid catalysis by polyoxometalate keggin clusters. J. Am. Chem. Sci. 130, 10369–71039. 10.1021/ja803114r 18613662

[B62] Mastikhin V. M. Kulikov S. M. Nosov A. V. Kozhevnikov I. V. Timofeeva M. N. (1990). 1H and 31P mas nmr studies of solid heteropolyacids and H_3_PW_12_O_40_ supported on SiO_2_. J. Mol. Catal. A Chem. 60, 65–70. 10.1016/0304-5102(90)85068-S

[B63] Mizuno N. Misono M. (1994). Heteropolyanions in catalysis. J. Mol. Catal. 86, 319–342. 10.1016/0304-5102(93)E0155-A

[B64] Mrowiec-Białoń J. Turek W. A. Jarzebski B. (2002). Preparation of highly active heteropolyacid-silica composite catalysts using the sol-gel method. React. Kinet. Catal. Lett. 76, 213–219. 10.1023/A:1016515407161

[B65] Nowinska K. Dudko D. Golon R. (1996). Pd_2_+Mn_2_+HPA: a heterogeneous wacker system catalyst. Chem. Commun. 2, 277–297. 10.1039/CC9960000277

[B66] Odyakov V. F. Zhizhina E. G. (2009). New process for preparing aqueous solutions of mo-v-phosphoric heteropoly acids. Russ. J. Inorg. Chem. 54, 361–367. 10.1134/S003602360903005X

[B67] Ogasawara Y. Itagaki S. Yamaguchi K. Mizuno N. (2011). Saccharification of natural lignocellulose biomass and polysaccharides by highly negatively charged heteropolyacids in concentrated aqueous solution. ChemSusChem 4, 519–525. 10.1002/cssc.201100025 21404445

[B68] Okuhara T. (2002). Water-tolerant solid acid catalysts. Chem Rev. 102, 3641–3665. 10.1021/cr0103569 12371897

[B69] Okuhara T. Nishimura T. Misono M. (1996). Novel microporous solid superacids: Cs_x_H_3−x_PW_12_O_40_ (2 < =x < =3). Stud. Surf. Sci. Catal. 101, 581–590. 10.1016/S0167-2991(96)80269-2

[B70] Okuhara T. Nishimura T. Watanabe H. Misono M. (1992). Insoluble heteropoly compounds as highly active catalysts for liquid-phase reactions. J. Mol. Catal. 74, 247–256. 10.1016/0304-5102(92)80242-9

[B71] Okuhara T. Watanabe H. Nishimura T. Inumaru K. Misono M. (2000). Microstructure of cesium hydrogen salts of 12-tungstophosphoric acid relevant to novel acid catalysis. Chem. Mater. 12, 2230–2238. 10.1021/cm9907561

[B72] Palkovits R. Tajvidi K. Ruppert A. Procelewska J. (2011). Heteropoly acids as efficient acid catalysts in the one-step conversion of cellulose to sugar alcohols. Chem. Commun. 47, 576–578. 10.1039/C0CC02263B 21103493

[B73] Pan T. Deng J. Xu Q. Zuo Y. Guo Q. X. Fu Y. (2013). Catalytic conversion of furfural into a 2,5-furandicarboxylic acid-based polyester with total carbon utilization. ChemSusChem 6, 47–50. 10.1002/cssc.201200652 23239596

[B74] Pang J. Wang A. Zheng M. Zhang T. (2010). Hydrolysis of cellulose into glucose over carbons sulfonated at elevated temperatures. Chem.Commun. 46, 6935–6937. 10.1039/c0cc02014a 20730212

[B75] Parghi K. D. Satam J. R. Jayaram R. V. (2011). Silica supported heteropolyacid catalyzed dehydration of aldoximes to nitriles and alcohols to alkenes. Green Chem. Lett. Rev. 4, 143–149. 10.1080/17518253.2010.523015

[B76] Reddy B. S. Narasimhulu G. Lakshumma P. S. Reddy Y. V. Yadav J. S. (2012). Phosphomolybdic acid: a highly efficient solid acid catalyst for the synthesis of trans-4, 5-disubstituted cyclopentenones. Tetrahedron Lett. 53, 1776–1779. 10.1016/j.tetlet.2012.01.115

[B77] Ren H. Girisuta B. Zhou Y. Liu L. (2015). Selective and recyclable depolymerization of cellulose to levulinic acid catalyzed by acidic ionic liquid. Carbohydr. Polym. 117, 569–576. 10.1016/j.carbpol.2014.09.091 25498672

[B78] Rinaldi R. Palkovits R. Schüth F. (2010). Depolymerization of cellulose using solid catalysts in ionic liquids. Angew. Chem. Int. Ed. 47, 8047–8050. 10.1002/anie.200802879 18814164

[B79] Shatalov A. A. (2019). Highly efficient hydrolysis of plant hemicelluloses by mixed-addenda keggin-type (Mo-V-P)-heteropolyacids in diluted aqueous solution. Carbohydr. Polym. 206, 80–85. 10.1016/j.carbpol.2018.10.106 30553386

[B80] Sheldon R. A. (2014). Green and sustainable manufacture of chemicals from biomass: state of the art. Green Chem. 16, 950–963. 10.1039/C3GC41935E

[B81] Shimizu K. Satsuma A. (2011). Toward a rational control of solid acid catalysis for green synthesis and biomass conversion. Energy Environ. Sci. 4, 3140–3153. 10.1039/c1ee01458g

[B82] Song I. K. Lee W. Y. (2004). Heteropolyacid (HPA)-polymer composite films as heterogeneous catalysts and catalytic membranes. Appl. Catal. A Gen. 256, 77–98. 10.1016/S0926-860X(03)00390-9

[B83] Su F. An S. Song D. Zhang X. Lu B. Guo Y. (2014). Heteropoly acid and ZrO_2_ bifunctionalized organosilica hollow nanospheres for esterification and transesterification. J. Mater. Chem. 2, 14127–14138. 10.1039/C4TA02257B

[B84] Sun Z. Cheng M. Li H. Shi T. Yuan M. Wang X. (2012). One-pot depolymerization of cellulose into glucose and levulinic acid by heteropolyacid ionic liquid catalysis. RSC Adv. 2, 9058–9065. 10.1039/c2ra01328b

[B85] Sun Z. Zhang X. Wang S. Li X. Wang X. Shi J. (2015). Hydrolysis and alcoholysis of polysaccharides with high efficiency catalyzed by a (C_16_TA)_x_H_6−x_P_2_W_18_O_62_ nanoassembly. RSC Adv. 5, 94155–94163. 10.1039/C5RA15047G

[B86] Tian J. Fang C. Cheng M. Wang X. (2011). Hydrolysis of cellulose over Cs_*x*_H_3−x_PW_12_O_40_ (x = 1–3) heteropoly acid catalysts. Chem. Eng. Technol. 34, 482–486. 10.1002/ceat.201000409

[B87] Tian J. Wang J. H. Zhao S. Jiang C. Y. Zhang X. Wang X. H. (2010). Hydrolysis of cellulose by the heteropoly acid H_3_PW_12_O_40_. Cellulose 17, 587–594. 10.1007/s10570-009-9391-0

[B88] To A. T. Chung P. W. Katz A. (2015). Weak-acid sites catalyze the hydrolysis of crystalline cellulose to glucose in water: importance of post synthetic functionalization of the carbon surface. Angew. Chem. Int. Ed. 127, 11202–11205. 10.1002/ange.201504865 26276901

[B89] Udayakumar S. Ajaikumar S. Pandurangan A. (2007). Electrophilic substitution reaction of phenols with aldehydes: enhance the yield of bisphenols by hpa and supported HPA. Catal. Commun. 8, 366–374. 10.1016/j.catcom.2006.05.054

[B90] Vázquez P. Pizzio L. Cáceres C. Blanco M. Thomas H. Alesso E. (2000). Silica-supported heteropolyacids as catalysts in alcohol dehydration reactions. J. Mol. Catal. A Chem. 16, 223–232. 10.1016/S1381-1169(00)00346-0

[B91] Wang H. Gurau G. Rogers R. D. (2012). Ionic liquid processing of cellulose. Chem. Soc. Rev. 41, 1519–1537. 10.1039/c2cs15311d 22266483

[B92] Wee L. H. Bajpe S. R. Janssens N. Hermans I. Martens J. A. (2010). Convenient synthesis of Cu_3_(BTC)_2_ encapsulated keggin heteropolyacid nanomaterial for application in catalysis. Chem. Commun. 46, 8186–8188. 10.1039/c0cc01447h 20927469

[B93] Wölfel R. Taccardi N. Bösmann A. Wasserscheid P. (2011). Selective catalytic conversion of biobased carbohydrates to formic acid using molecular oxygen. Green Chem. 13, 2759–2763. 10.1039/c1gc15434f

[B94] Wu L. Zhu B. Wu Y. (2017). Hydrophobic lurylamine modified heteropoly acid as an efficient and recyclable catalyst for the hydrolysis reaction in aqueous solution under microwave, in The 4th Annual International Conference on Information Technology and Applications (ITA 2017) (Guangzhou), 12:04026 10.1051/itmconf/20171204026

[B95] Wu Y. Fu Z. Yin D. Xu Q. Liu F. Lu C. (2010). Microwave-assisted hydrolysis of crystalline cellulose catalyzed by biomass char sulfonic acids. Green Chem. 12, 696–700. 10.1039/b917807d

[B96] Xie X. Han J. Wang H. Zhu X. Liu X. Niu Y. (2014). Selective conversion of microcrystalline cellulose into hexitols over a Ru/[Bmim]_3_PW_12_O_40_ catalyst under mild conditions. Catal. Today 233, 70–76. 10.1016/j.cattod.2013.09.061

[B97] Yabushita M. Kobayashi H. Fukuoka A. (2014). Catalytic transformation of cellulose into platform chemicals. Appl. Catal. B Environ. 145, 1–9. 10.1016/j.apcatb.2013.01.052

[B98] Yan L. Greenwood A. A. Hossain A. Yang B. (2014). A comprehensive mechanistic kinetic model for dilute acid hydrolysis of switchgrass cellulose to glucose, 5-HMF and levulinic acid. RSC Adv. 4, 23492–23478. 10.1039/c4ra01631a

[B99] Yang L. Qi Y. Yuan X. Shen J. Kim J. (2005). Direct synthesis, characterization and catalytic application of SBA-15 containing heteropolyacid H_3_PW_12_O_40_. J. Mol. Catal. A Chem. 22, 199–205. 10.1016/j.molcata.2004.11.024

[B100] Yang P. Kobayashi H. Fukuoka A. (2011). Recent developments in the catalytic conversion of cellulose into valuable chemicals. Chin. J. Catal. 32, 716–722. 10.1016/S1872-2067(10)60232-X

[B101] Yu F. Kong X. J. Zheng Y. Y. Ren Y. P. Long L. S. Huang R. B.. (2009a). Ph-dependent assembly of 0D to 3D keggin-based coordination polymers: structures and catalytic properties. Dalton Trans. 43, 9503–9509 10.1039/b911606k 19859606

[B102] Yu S. Brown H. M. Huang X. Zhou X.-,d. Amonette J. E. Zhang Z. (2009b). Single-step conversion of cellulose to 5-hydroxymethylfurfural (HMF), a versatile platform chemical. Appl. Catal. A Gen. 361, 117–122. 10.1016/j.apcata.2009.04.002

[B103] Yusuke K. Urabe K. (1981). Catalysis of heteropoly acids entrapped in activated carbon. Chem. Lett. 10, 663–666,. 10.1246/cl.1981.663

[B104] Zhang F. Deng X. Fang Z. Zeng H. Tian X. Kozinski J. (2011). Hydrolysis of microcrystalline cellulose over Zn-Ca-Fe oxide catalyst. Petrochem. Technol. 40, 43–48. 10.1016/S1872-5805(11)60067-X

[B105] Zhang H. Li H. Pan H. Wang A. Souzanchi S. Xu C. (2018). Magnetically recyclable acidic polymeric ionic liquids decorated with hydrophobic regulators as highly efficient and stable catalysts for biodiesel production. Appl. Energ. 223, 416–429. 10.1016/j.apenergy.2018.04.061

[B106] Zhang H. Zhou Q. Chang F. Pan H. Liu X. Li H. (2015). Production and fuel properties of biodiesel from Firmiana platanifolia L.f. as a potential non-food oil source. Ind. Crop. Prod. 76, 768–771. 10.1016/j.indcrop.2015.08.002

[B107] Zhang J. Liu X. Sun M. Ma X. Han Y. (2012). Direct conversion of cellulose to glycolic acid with a phosphomolybdic acid catalyst in a water medium. ACS Catal. 2, 1698–1702. 10.1021/cs300342k

[B108] Zhang J. Sun M. Liu X. Han Y. (2014). Catalytic oxidative conversion of cellulosic biomass to formic acid and acetic acid with exceptionally high yields. Catal. Today 233, 77–82. 10.1016/j.cattod.2013.12.010

[B109] Zhang X. Zhang D. Sun Z. Xue L. Wang X. Jiang Z. (2016). Highly efficient preparation of HMF from cellulose using temperature-responsive heteropolyacid catalysts in cascade reaction. Appl. Catal. B Environ. 196, 50–56. 10.1016/j.apcatb.2016.05.019

[B110] Zhang Y. Zhao M. Wang H. Hu H. Liu R. Huang Z.. (2019). Damaged starch derived carbon foam-supported heteropolyacid for catalytic conversion of cellulose: improved catalytic performance and efficient reusability. Bioresour. Technol. 288:121532. 10.1016/j.biortech.2019.121532 31146077

[B111] Zhao Q. Wang H. Zheng H. Sun Z. Shi W. Wang S. (2013). Acid–base bifunctional hpa nanocatalysts promoting heterogeneous transesterification and esterification reactions. Catal. Sci. Tech. 3, 2204–2209. 10.1039/c3cy20868k

[B112] Zheng W. Cui Y. Xu Z. Zhao L. Sun W. (2018). Cellulose transformation into methyl glucosides catalyzed by H_3_PW_12_O_40_: enhancement of ionic liquid pretreatment. Can. J. Chem. Eng. 96, 1250–1255. 10.1002/cjce.23057

[B113] Zhizhina E. G. Odyakov V. F. (2008). Alteration of the physicochemical properties of catalysts based on aqueous solutions of mo-v-p heteropoly acids in redox processes. Reac. Kinet. Catal. Lett. 95, 301–312. 10.1007/s11144-008-5423-2

